# Global research priorities for intrauterine suction and sponge tools for postpartum haemorrhage management in low-income and middle-income countries: a modified Delphi approach

**DOI:** 10.1136/bmjph-2023-000113

**Published:** 2024-05-30

**Authors:** Dilys Walker, Linda Shin, Nicole Santos, Elizabeth Butrick, Jill Durocher, Suellen Miller, Ribka Amsalu, Anthony Wanyoro

**Affiliations:** 1 Bixby Center for Global Reproductive Health, Department of Obstetrics, Gynecology & Reproductive Sciences, University of California, San Francisco, California, USA; 2 Institute for Global Health Sciences, University of California San Francisco, San Francisco, California, USA; 3 School of Medicine, California University of Science and Medicine, Colton, California, USA; 4 Gynuity Health Projects, New York, New York, USA; 5 Department of Obstetrics, Gynecology & Reproductive Sciences, UCSF, San Francisco, California, USA; 6 Department of OB/GYN, Kenyatta University, Nairobi, Kenya

**Keywords:** Public Health Practice, Patient Harm, Consumer Product Safety, Community Health

## Abstract

**Introduction:**

Postpartum haemorrhage (PPH) remains the leading cause of maternal mortality and morbidity globally. Innovative PPH management tools have emerged using suction and sponge tamponade but currently lack substantial evidence. Broader understanding and collaborative research prioritisation are needed, especially in low-income and middle-income countries (LMICs), where the burden of PPH-related mortality is highest. We aimed to describe the current state of evidence and to solicit stakeholder input to identify research priorities related to emerging tools for PPH management.

**Methods:**

We used a four-phase modified Delphi approach to identify research priorities for emerging suction and sponge tools. In phase 1, we conducted a literature review and key informant interviews (KIIs) with 19 stakeholders. In phase 2, we distributed an online survey, receiving 66 responses. In phase 3, we virtually convened an expert panel of stakeholders (n=24) and a separate midwife-only focus group to discuss preliminary results and draft research questions. In phase 4, we surveyed our expert panel (n=37) for prioritisation of research questions. Surveys were disseminated via Research Electronic Data Capture while KIIs and the expert convening were held virtually.

**Results:**

Participants included clinicians, researchers, policy-makers, funders and tool developers from high-income and LMIC settings. The prioritisation process narrowed our focus from six tools to four, all of which were top-ranked priorities in phase 4. Stakeholders emphasised efficacy research in comprehensive emergency obstetric and newborn care facilities. Stakeholders stressed the importance of understanding adverse event risks. The urgency in conducting research on cost, provider ease of use and acceptability and patient experience differed between individuals from high-income versus LMIC settings.

**Conclusion:**

All four tools prioritised in this process have the potential to improve PPH management in LMICs. A coordinated research agenda is necessary to confirm safety and efficacy and to determine which tools are most appropriate for specific LMIC settings.

**WHAT IS ALREADY KNOWN ON THIS TOPIC**

**WHAT THIS STUDY ADDS**

- Stakeholders prioritised efficacy research, research conducted in comprehensive emergency obstetric and newborn care facilities, and a better understanding of adverse event risks. Perspectives on the urgency of conducting research on cost, provider ease of use and acceptability, and patient experience differed between those from high-income versus LMIC settings.

**HOW THIS STUDY MIGHT AFFECT RESEARCH, PRACTICE OR POLICY**

- Four devices emerged as high priorities for research to improve PPH management in LMICs. A coordinated research agenda is essential to confirm device safety, efficacy and appropriate tools for LMIC settings.

## Introduction

 The WHO’s 2023 publication reporting on trends in global maternal mortality reveals disappointingly little movement in the past 5 years.^1^ Postpartum haemorrhage (PPH) remains the leading cause of maternal mortality globally. However, there is a great disparity in the prevalence, where in high-income countries (HICs), it ranges between 7% and 12% whereas the prevalence reaches 26% in sub-Saharan Africa.^2 3^ In 2017, PPH caused over 38 000 deaths, of which more than 90% occurred in low-income and middle-income countries (LMICs).^4 5^ Survivors of severe PPH can also face severe morbidity, including organ failure, disseminated intravascular coagulation, intensive care unit admission and hysterectomy.^6^

Current literature shows that existing management approaches have limited efficacy, as PPH continues to be the leading contributor to maternal mortality.^7^ Further, access to critical interventions is often lacking in low-resource settings, contributing to high morbidity and mortality.^7^,^9^ Uterine balloon tamponade (UBT) is the most recent addition to guidelines on PPH management and is seen as a last step to control refractory haemorrhage prior to more invasive strategies.^10 11^ However, questions remain about the efficacy of UBT due to conflicting data from randomised and non-randomised studies.^12^ Inconsistent data have led to conflicting recommendations, with the WHO recommending UBT use in the context of availability of higher level care and consistent monitoring, whereas the Federation of International Gynecologists and Obstetricians (FIGO) recommends more general use.^10 13^

The high burden of PPH continues to generate interest in developing new tools to improve PPH management and outcomes. New suction and sponge tools are at the forefront of innovation for haemorrhage uncontrolled by first-line interventions. Vacuum-induced uterine tamponade tools, such as the FDA-approved Jada system^14 15^ apply low-level intrauterine suction to evacuate blood and facilitate physiological uterine contraction in patients with PPH due to atony. There is a long history of uterine packing control for PPH with variable success.^16^ Newer innovations include the use of rapidly expanding sponge technology (XSTAT)^17^ and gauze treated with a chemical coagulant (Celox).^18^

As the number and variety of PPH management tools emerge, there is a growing need to systematically better understand and inform research efforts that are contextually appropriate for a given setting. These tools necessitate varying degrees of resources, provider skills and infrastructure for their effective implementation and may lead to differences in research priorities in high-resource versus resourced-constrained settings. Given the continued high global burden of PPH-related maternal morbidity and mortality, particularly in LMICs, it is necessary to review the existing evidence for these tools and establish a common research agenda to inform providers, health systems and policy-makers as management guidelines evolve. The agenda should acknowledge the important voice of remote end-users, specifically midwives.

The objective of this study was to describe priority research questions for these emerging suction and sponge tools for use in LMIC settings. We aimed to integrate input from a broad range of stakeholders and strengthen collaborative research efforts that can inform and accelerate WHO guideline development relevant to LMICs.

## Methods

### Study design

**Figure 1:**
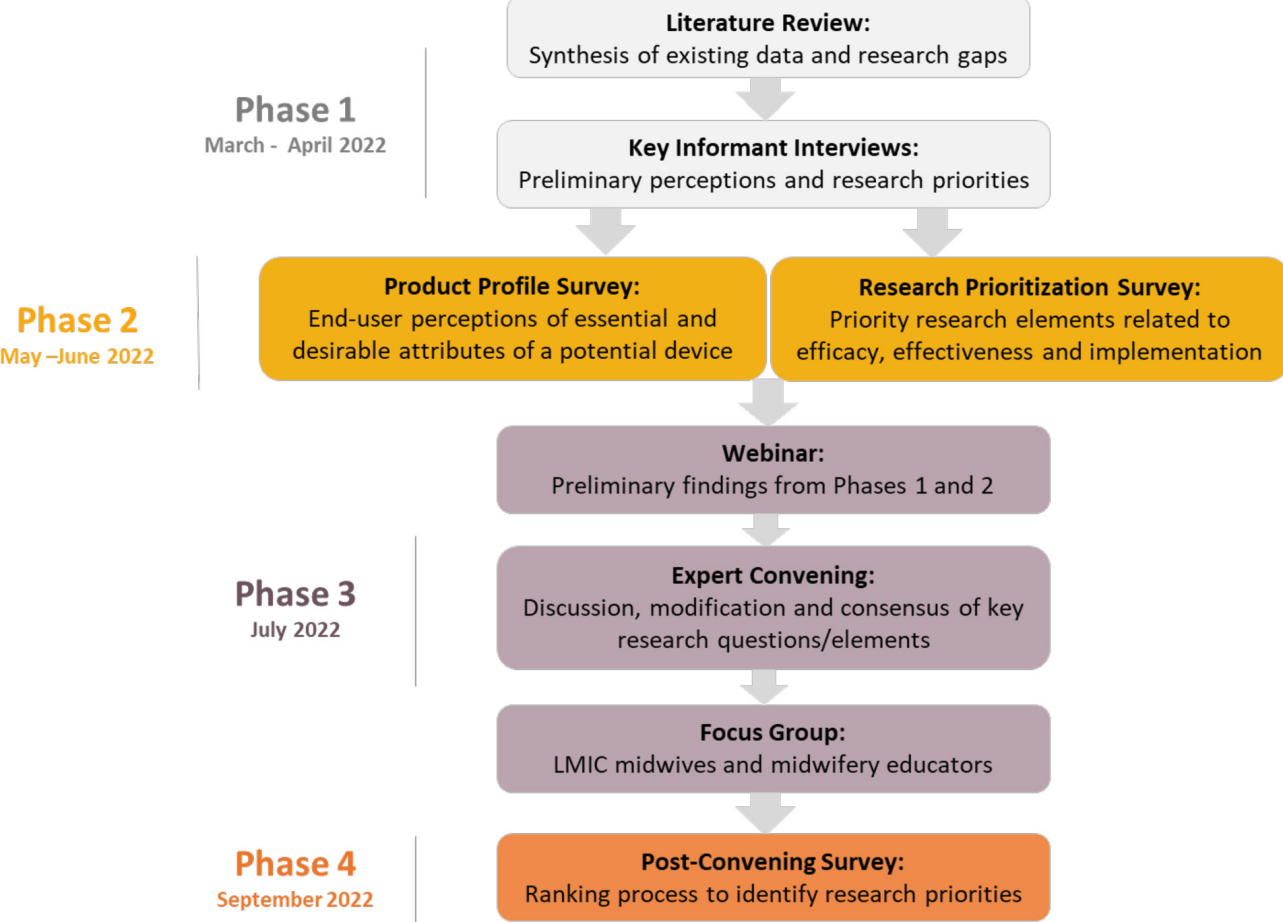
Study flow chart. Figure 1 outlines the methodology used in this four-phase study conducted from March to September 2022. Phase 1 begins with a literature review that synthesises existing data and identifies research gaps. This review is followed by key informant interviews that explore preliminary perceptions and research priorities. Phase 2 involves two surveys: a Product Profile Survey that gathers end-user perceptions of essential and desirable attributes of a potential device and a Research Prioritisation Survey that collects priority research elements related to efficacy, effectiveness and implementation. Phase 3 starts with a webinar that disseminates preliminary findings from phases 1 and 2 and follows with an expert convening aimed at discussion, modification and consensus of key research questions and elements. Phase 3 concludes with a focus group of LMIC midwives and midwifery educations. Phase 4 involves a Post-Convening Survey that includes a ranking process to identify research priorities. LMIC, low-income and middle-income country.

We used a modified Delphi method to establish expert consensus regarding research priorities for emerging PPH suction and sponge tools. The Delphi method is a well-established, interactive technique used to achieve consensus among experts. By eliciting input from individuals in an iterative and anonymous process, a preliminary list is reduced to a shortlist of priorities.^19^ Our modified approach included four phases, described below and in figure 1. We sought directed input from both LMIC and HIC practitioners and stakeholders working in the PPH field drawing from academic, implementation, and policy perspectives. To address missing data in survey responses, we reported the proportion of participants who provided a response for each question and conducted a thorough review of all questions to identify any high non-response rates.

### Phase 1: information synthesis

Phase 1 of the study focused on information synthesis through literature review and key informant interviews (KIIs) to identify candidate tools and to understand existing evidence and research gaps to inform the development of the first survey. A summary of published literature was shared with key informants and Delphi participants to ensure base knowledge about tools.

The literature review consisted of (1) WHO and FIGO guidelines and recommendations, (2) clinical trial searches (eg, ClinicalTrials.gov; WHO ICTRP) and (3) a synthesis of peer-reviewed literature (PubMed) and grey literature (Google research). The synthesis included literature related to PPH management published in the last 10 years. The following search terms were used: postpartum haemorrhage, suction, vacuum, sponge and tamponade. We excluded conference abstracts and studies focused on UBT. We used snowball referencing, including information gained from KIIs.

For the KIIs, we identified individuals and thought leaders in the field from WHO, funding agencies, academia, tool developers/champions, and midwifery and medical educators, considering the institutional role, publication history, and experience in LMIC settings. 22 individuals were sent an email invitation that outlined the study. If they agreed to participate, we conducted a Zoom interview using one of two interview guides depending on their background. Tool developers/champions (individuals working on tool development or pilot research) were questioned about prior PPH research, next steps and perceived advantages. Clinicians, funders, implementers, educators and policy-makers were questioned about prior knowledge about these tools, perceptions and research priorities. Efforts were made to include representation from both high-resource and low-resource settings, particularly clinicians and educators.

Each interview was conducted for approximately 1 hour via Zoom and facilitated by the research team. Interviews were recorded with accompanying note-taking. Data synthesis included matrix mapping and drawing quotes from interviews representative of the different professional perspectives.

### Phase 2: first Delphi survey

In phase 2, a research prioritisation survey about identified tools was disseminated broadly to stakeholders in the PPH field (online supplemental file 1). Recruitment involved snowball dissemination to over 500 individuals globally including outreach to professional networks, authors who have published in relevant PPH tool literature, PPH Community of Practice, FIGO subcommittee and obstetrics and gynaecology associations. Questions elicited input on critical areas of research spanning efficacy (defined as assessment of clinical outcomes/safety in highly controlled research environments), effectiveness (defined as assessment of clinical outcomes in practice/real-world settings) and implementation research (defined as research-related to factors influencing scale, such as cost, training/skill retention and supply chain). While knowledge, perceptions and general research priority questions were not specific to individual tools, questions related to designing an efficacy study asked respondents to select a preferred or priority tool. Feasibility and acceptability were also captured, exploring provider experience (eg, ease of use) and patient experience (eg, pain, discomfort).

Given that our target recruitment included LMIC practitioners who might have limited knowledge or exposure to the tools being discussed, we included an alternate survey version that focused on the ideal characteristics of any new product from the user perspective (online supplemental file 2). Participants could choose to answer either one or both surveys. We used the product profile survey to refine our ideas about research priorities, and the results are available on our website.^20^

Prior to deployment, the surveys were piloted with three content experts, and revisions were made based on their feedback. Surveys were created on UCSF Research Electronic Data Capture and shared through a public link.^21 22^ Surveys were open from 11 May 2022 to 16 June 2022 and accompanied by weekly reminders. The default design preserved anonymity to ensure independence of judgement and confidentiality. Participants could submit contact information voluntarily.

Data were analysed by using SPSS V.26 and visualisations were created in Excel. At the conclusion of phase 2, we held a dissemination webinar to share preliminary results and to invite reflections from selected LMIC and WHO stakeholders. The webinar was open to the public with 81 participants.

### Phase 3: expert convening

Phase 3 included an expert convening with the following objectives: (1) to share existing information about intrauterine, non-balloon tools that are emerging for PPH management, (2) to agree on a set of guiding principles for research regarding these emerging tools, (3) to identify key questions that should be addressed in future research for selected tools and (4) to agree on a mechanism to prioritise and share conclusions to maximise impact.

A virtual convening was held for 2 hours on 21 July 2022, with a target of 25 participants. Invitees (N=37) included WHO, funders, prominent researchers, clinicians and other key stakeholders in the field of PPH, with the intention of ensuring diverse perspectives. We identified leaders in PPH based on our landscaping, all with extensive LMIC PPH experience and sent individual invitations by email. These individuals were identified as influential stakeholders through our landscaping interviews. We excluded any stakeholders or industry representatives with biases towards one device or another. To include diverse opinions and priorities, we targeted active clinicians (obstetricians and midwives), investigators, policy-makers and funders.

The convening included a review of survey results, live polls, a research mapping exercise, the creation of a word cloud and breakout sessions for research question development. Overall, the convening aimed to facilitate open dialogue used to inform the second Delphi survey.

Following the phase 3 convening, we convened a focus group of an important end-user group for these devices, LMIC midwives and midwifery educators. While midwives participated in the convening, we felt the LMIC midwife perspective was under-represented. After filing an IRB amendment, we invited 10 midwives and educators to a Zoom focus group where we discussed many of the same issues in the convening.

### Phase 4: second Delphi survey

Following the convening, we sent a second research prioritisation survey to all convening invitees including those who were unable to participate in the convening (N=37). The second survey focused on the four tools identified as a higher priority from phases 2 and 3. Questions related to Jada assumed a modified Jada device appropriate for an LMIC setting (eg, lower cost, reusable).

Survey questions were refined from preliminary research questions generated by convening participants and organised across the continuum of research stages, from pretrial, efficacy and effectiveness, to implementation. The questions were structured according to the level of care provided, either basic or comprehensive emergency obstetric and newborn care (BEmONC/CEmONC). We asked 24 questions about the efficacy and effectiveness of the four prioritised tools (online supplemental file 3).

For each tool, respondents were asked to indicate whether the questions should be kept, modified or removed and to rank priority from high to low; priority scores were based on the proportion of respondents ranking a question as high priority, with tied rankings resolved by the proportion that rated an item as medium priority. No a priori threshold was set. For each tool, respondents were also asked to prioritise research on its use as the next line of action when bleeding continues after administering uterotonics, tranexamic acid and giving fluids intravenously (referred to as the PPH first response treatment bundle) or after all available measures have failed (after using all available uterotonics or compression techniques) as the last step prior to referral or surgery.^23^ There was an additional set of 27 questions related to other priorities (online supplemental file 3), such as provider ease of use and cost, that were individually ranked as high, medium or low priority.

Priority designations were used to create an overall ranking of research questions stratified by tool and whether respondents were from LMICs or HICs. Descriptive statistics were used to describe characteristics of survey respondents while priority ranking was analysed using weighted scores which combined whether a question should be kept (or kept with modifications) and its priority ranking.

### Patient and public involvement

While this study does not directly involve patients or the public, we recognise the importance of incorporating their experiences and perspectives to ensure that our research is meaningful and relevant to those affected by PPH. In the development of our research questions and survey instruments, we consulted with patient representatives to ensure that the questions being studied were relevant to patients’ needs in both LMICs and HICs.

## Results

Table 1 synthesises the key outputs and findings from the first three phases of this work. Phase 1 identified 14 published papers related to 6 tools (online supplemental file 4). Three of these PPH management tools are purpose-built, including the FDA-approved Jada, the Panicker suction tool and the XSTAT sponge while the others are improvised or repurposed (eg, Celox gauze, modified Bakri and the Levin gastric tube). Celox and the modified Bakri had no available published studies conducted in an LMIC setting, and only the Levin gastric tube had a reported randomised controlled trial in process.^24^ There was no standardised primary outcome in the 14 studies reviewed.

**Table 1:** Key findings from phases 1 to 3

	Participants*	Key outputs/findings	Detailed information
**Phase 1**			
Literature review	Not applicable	4 purpose-built tools and 2 improvised/repurposed tools identified 14 published papers 2 tools with no LMIC studies; only one RCT; no standardised primary outcome	Online supplemental file 4
Key informant interviews	19(21% LMIC residents)	Opinion split on purpose-built vs improvised tools Agreement that efficacy has not been demonstrated, but effectiveness and scale-up are important to consider Need for target product profile that aligns with context/setting Need for coordination around research efforts	Online supplemental file 5 https://www.youtube.com/watch?v=DyHX9dFrmgo
**Phase 2**			
First Delphi research prioritisation survey	66(52% LMIC residents)	Tools hold a lot of potential to overcome PPH practice gaps Purpose-built tools were of higher interest than improvised ones Effectiveness research was a top priority, as shown by the desire to test tools across different facility types and to examine ease of use by different provider cadres and women’s experience	Online supplemental files 1; 6
Alternate device characteristics survey	89(57% LMIC residents)	Strong interest in tools that could be used in multiple settings by varying level of providers Strong preferences for tools that can be used in less than 2 min by a single provider, stored at room temperature and are low cost	Online supplemental files 2; 7
**Phase 3**			
Expert convening	23(39% LMIC residents)	Efficacy research in CEmONC facilities was a top prioritiy	Not applicable

* The majority of participants who identified as non-LMIC residents had extensive knowledge of both PPH and LMIC contexts.

CEmONC, comprehensive emergency obstetric and newborn care; LMIC, low-income and middle-income country; PPH, postpartum haemorrhage; RCT, randomised controlled trial.

Among the 22 individuals invited to the KIIs, 19 in-depth interviews were completed (3 did not respond to multiple email attempts) with 21% of the interviewees from LMICs. This included six developers/champions, seven researchers and six funders. Table 1 lists the key findings from interviews with notable divergence in opinions around the value of purpose-built (eg, Jada, XSTAT) versus improvised tools (eg, Levin gastric tube) and the appropriate settings for these tools. There was convergence on the need for efficacy studies, studies in LMIC settings, standardised outcomes and coordinated research efforts. Themes from these KIIs are summarised in online supplemental file 5, including divergent opinions regarding purpose-built ‘bespoke’ devices versus improvised tools.

In phase 2, we invited over 500 individuals to participate in the first Delphi study. We received 66 completed surveys with 52% of respondents residing in LMICs. The 66 total respondents included 36 clinicians, 7 researchers, 2 policy-makers, 3 from the private sector/industry, 2 implementors and 16 with other/mixed roles. Overall, participants reported that these tools hold great promise for managing PPH. Among these respondents, effectiveness research was considered a high priority with a need to test tools in different facility settings, including non-CEmONC settings. Purpose-built tools were prioritised over improvised tools. Results from this survey, as well as the product profile survey, are provided in onlinesupplemental files 6^7^.

In phase 3, we invited 37 individuals (17 from LMICs and 20 from HICs—90% of whom had extensive LMIC experience, and 10% who were funders with more limited LMIC experience) to the expert convening. Of the invitees, 24 attended, including 14 obstetricians, 6 midwives, 3 researchers and 1 funder (some identified multiple roles, eg, 17 clinicians were also researchers). Experts reviewed the results, voted on priority areas for each tool and developed draft research questions to inform the final Delphi survey. Based on the results of the previous phases, we focused on four specific tools that had shown the most promise: Jada, Levin gastric tube, XSTAT and Celox. The majority of respondents emphasised the need for efficacy research in CEmONC settings with research and clinical safeguards in place.

Of the 16 LMIC midwives invited to our phase 3 focus group, 9 accepted the invite and 6 attended. The information gathered during the focus group largely aligned with the findings from the other workstreams. However, frustration was expressed at the general failure to include midwives as primary users in early research and introduction efforts, instead opting to start with physicians and specialists. Participants described their perception that midwives would be more comfortable with tools inserted manually with their hands (eg, Celox gauze) and less comfortable with applicators or devices. The most notable insight from this focus group, reinforced by the results of the product characteristics survey, is the importance of including midwives as primary users during any efficacy and effectiveness research.

In phase 4 of our Delphi approach, the survey was distributed to the 37 convening invitees, and we received a total of 29 responses, for a response rate of 78%. The response rate was 85% (17/20) for HIC respondents and 71% (12/17) for LMIC respondents. Most respondents were doctors or researchers, with a higher proportion of researchers in the HIC group and a higher proportion of doctors in the LMIC group. Most respondents have over 10 years of experience in the field of PPH (table 2).

**Table 2:** Demographics of phase 4 Delphi survey respondents

	Total	HIC residents	LMIC residents
**Reside in an LMIC?**	**29**	**17** (**59%**)	**12** (**41%**)
Profession			
Doctor	11 (38%)	3 (18%)	8 (67%)
Nurse/midwife	3 (10%	1 (6%)	2 (17%)
Researcher	9 (31%)	8 (47%)	1 (8%)
Private sector/industry	2 (7%)	2 (12%)	–
Policy/government	3 (10%)	1 (6%)	2 (17%)
Philanthropy	2 (7%)	2 (12%)	–
Other	1 (3%)	1 (6%)	–
Years of experience			
0–5	1 (3%)	1 (6%)	–
6–10	2 (7%)	1 (6%)	1 (8%)
11–20	6 (21%)	5 (30%)	1 (8%)
20+	19 (66%)	10 (59%)	9 (75%)

HIC, high-income country; LMIC, low-income and middle-income country.

We compiled the top 10 questions about the efficacy and effectiveness of the four tools in BEmONC and CEmONC settings by overall ranking in table 3. For each question, 76%–86% of respondents voted to keep or modify the question, and 31%–69% of these are ranked as high priority. Each of the four tools is represented among the top 10 research questions; 8 of the top 10 questions focus on efficacy while the remaining two focus on effectiveness for the Jada device. In general, HIC respondents prioritise the Jada device while LMIC respondents prefer the other tools.

**Table 3:** Top 10 efficacy and effectiveness questions based on phase 4 Delphi survey responses

Question	Overall rank	HIC rank	LMIC rank
In an adequately resourced CEmONC setting in an LMIC, what is the efficacy and safety of the modified Jada device compared with standard of care in the reduction of PPH-related maternal morbidity and mortality among women who experience refractory PPH due to uterine atony?	1	2	3
In adequately resourced CEmONC settings with ability to respond to PPH, what is the efficacy and safety of using Celox gauze compared with standard of care in the reduction of PPH-related maternal morbidity and mortality among women who experience PPH due to atony who do not respond to the first response bundle?	2	6	4
In an adequately resourced CEmONC setting with ability to respond to PPH, what is the efficacy and safety of using Celox gauze compared with standard of care in the reduction of PPH-related maternal morbidity and mortality among women who experience refractory PPH due to uterine atony?	3	3	2
In an adequately resourced CEmONC setting in an LMIC, what is the efficacy and safety of the modified Jada device compared with standard of care in the reduction of PPH-related maternal morbidity and mortality among women who experience PPH due to atony who don't respond to the first response bundle?	4	1	12
In an adequately resourced CEmONC setting in an LMIC, what is the efficacy and safety of using the XSTAT in the reduction of PPH-related maternal morbidity and mortality among women who experience PPH due to atony who do not respond to the first response bundle?	5	10	1
In adequately resourced CEmONC setting in an LMIC, what is the effectiveness of using the modified Jada device compared with standard of care in the reduction of PPH-related maternal morbidity and mortality among women who experience PPH due to atony who do not respond to the first response bundle?	6	4	7
In an adequately resourced CEmONC setting in an LMIC, what is the efficacy and safety of using XSTAT compared with standard of care in the reduction of PPH-related maternal morbidity and mortality among women with refractory PPH due to uterine atony?	7	6	5
In an adequately resourced CEmONC setting in an LMIC, what is the efficacy and safety of the Levin gastric tube compared with standard of care in the reduction of PPH-related maternal morbidity and mortality among women who experience PPH due to atony who do not respond to the first response bundle?	8	8	6
In an adequately resourced CEmONC setting in an LMIC, what is the efficacy and safety of the Levin gastric tube compared with standard of care in the reduction of PPH-related maternal morbidity and mortality among women who experience refractory PPH due to uterine atony?	9	9	9
In adequately resourced CEmONC setting in an LMIC, what is the effectiveness of using the modified Jada device compared with standard of care in the reduction of PPH-related maternal morbidity and mortality among women who experience refractory PPH due to uterine atony?	10	7	8

HIC, high-income country; LMIC, low-income and middle-income country.

**Figure 2:**
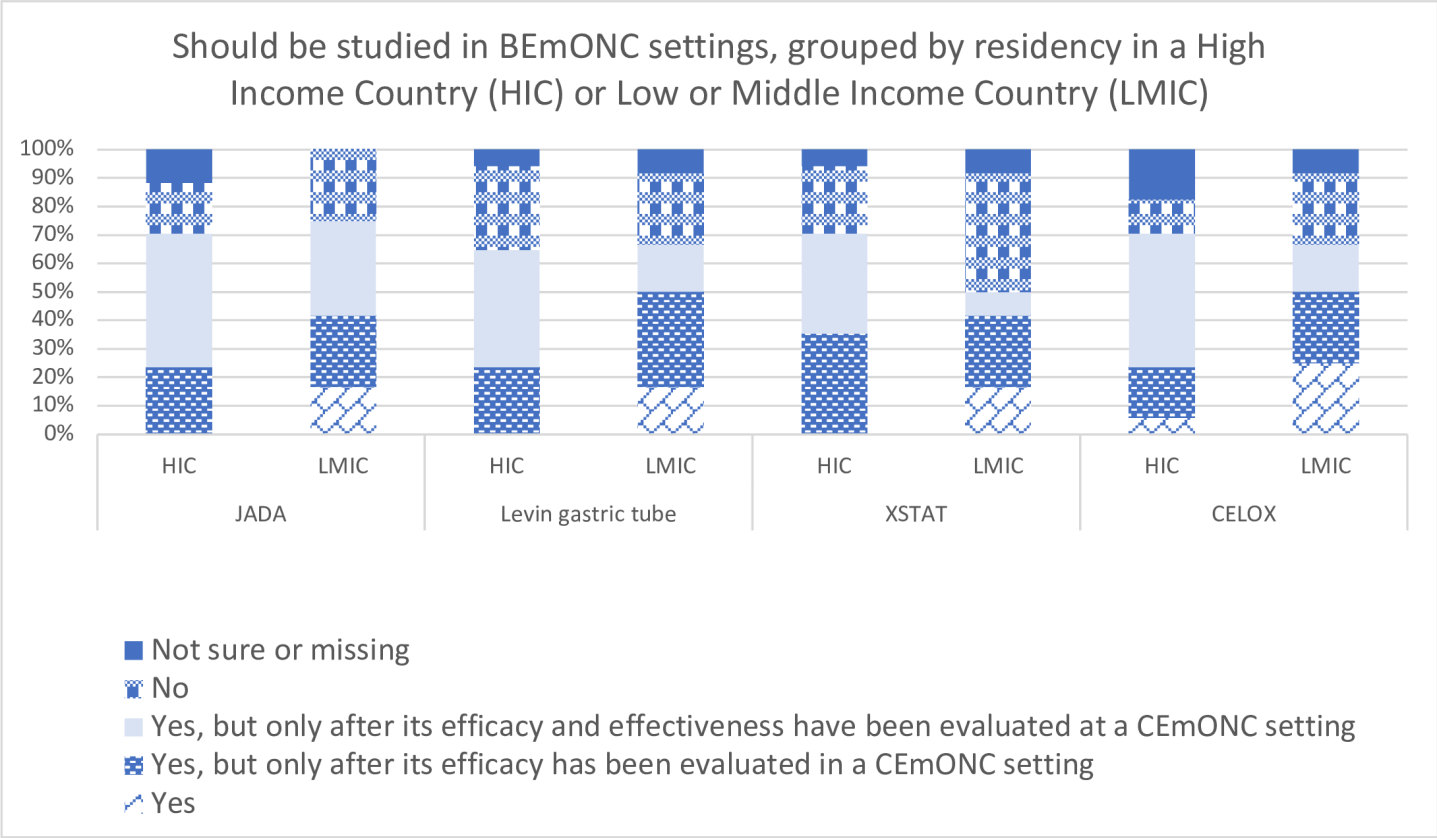
Perceptions related to research for tools in basic emergency obstetric and newborn care (BEmONC) settings This figure displays a bar chart that shows the distribution of responses to the question, ‘Should be studied in Basic Emergency Obststric and Newborn Care (BEmONC settings?’ by residency in a high-income country (HIC) or low-income or middle-income country (LMIC) for four medical devices: JADA, Levin gastric tube, XSTAT and CELOX. The responses consist of five options: ‘not sure or missing’, ‘no’, ‘yes, but only after its efficacy and effectiveness have been evaluated at a CEmONC setting’, ‘yes, but only after its efficacy has been evaluated in a CEmONC setting’ and ‘yes’. For each device, there are two bars representing respondents residing in HICs and LMICs, respectively. The length of each bar segment corresponds to the percentage of respondents within the country income group for a specified device who selected the associated response option. The figure visualises the varying research priorities for medical devices in BEmONC settings across income groups. CEmONC, comprehensive emergency obstetric and newborn care.

Questions related to the timing of tool use, either immediately after the initial response or later in the treatment algorithm just before transfer, emerge in the top 10 list. There was greater support for conducting research at the CEmONC level, with 31% of all respondents and 41% of LMIC respondents expressing that research on these tools should not be conducted in BEmONC settings (figure 2).

The top 10 questions related to other research priorities are listed in table 4. These questions were ranked as high priority by 77%–94% of respondents and included research pertaining to each of the four tools. Questions related to better understanding risks are in the top ten list. Usability and acceptability questions are important for Jada and Celox, cost is important for Jada, and patient experience is important for both suction tools. Celox is the only tool for which there was significant prioritisation of exploring use for non-atony-related causes of PPH. For additional research priorities, there are differences between HIC and LMIC respondents, with LMIC respondents placing higher priority on the Levin gastric tube and usability, acceptability and cost ranked lower across all tools.

**Table 4:** Top 10 other research priorities based on phase 4 Delphi survey responses

Question	Overall rank	HIC rank	LMIC rank
What are the risks of adverse events, such as perforation, infection, inadequate placement (placement in the vagina, instead of uterus) for the Jada device?	1	1	1
What are the risks of adverse events, such as infection, inadequate placement (placement in the vagina, instead of uterus) for the Celox gauze?	2	2	3
What are the risks of adverse events, such as perforation, infection, inadequate placement (placement in the vagina, instead of uterus) for the Levin gastric tube?	3	9	4
What is the usability and acceptability (eg, ease and speed of use) of the Jada device from the provider perspective?	4	3	10
What are the costs associated with use of the Jada device for PPH management compared with standard of care?	5	4	11
What is the usability and acceptability (eg, ease and speed of use) of the Celox gauze from the provider perspective?	6	5	13
What are the risks of adverse events, such as infection, inadequate placement (placement in the vagina, instead of uterus) for the XSTAT sponge?	7	6	12
To inform acceptability in LMICs where many women do not receive regional anaesthesia, what is the patient experience (eg, pain, discomfort) if the Levin gastric tube is used?	8	17	2
Can the Celox gauze be used for any cause of PPH (eg, placenta bed abnormality, vaginal/cervical tears)?	9	13	15
To inform acceptability in LMICs where many women do not receive regional anaesthesia, what is the patient experience (eg, pain, discomfort) if the Jada device is used?	10	15	5

HIC, high-income country; LMIC, low-income and middle-income country.

To better understand the highest priority for each tool, we compiled the top three priorities by tool (table 5). The top three efficacy questions are the same across all tools, with a slight variation for Jada in that participants prioritise exploring its use in cases as a last intervention prior to surgery rather than after failed first response.

**Table 5:** Top three research priorities for each tool based on phase 4 Delphi survey responses

	Jada suction device	Celox gauze	XSTAT sponge	Levin gastric tube
Efficacy and effectiveness	Efficacy for refractory PPH in CEmONC Efficacy after first response in CEmONC Effectiveness after first response in CEmoNC	Efficacy after first response in CEmONC Efficacy for refractory PPH in CEmONC Effectiveness for refractory PPH in CEmONC	Efficacy after first response in CEmONC Efficacy for refractory PPH in CEmONC Effectiveness for refractory PPH in CEmONC	Efficacy after first response in CEmONC Efficacy for refractory PPH in CEmONC Effectiveness for refractory PPH in CEmONC
Other	Risk of adverse events Usability and acceptability Cost	Risk of adverse events Usability and acceptability Usability for other causes of PPH	Risk of adverse events Usability and acceptability Patient experience	Risk of adverse events Patient experience Usability and acceptability

CEmONC, comprehensive emergency obstetric and newborn care; PPH, postpartum haemorrhage.

## Discussion

With the variety of sponge and suction tools and devices on the horizon for the management of PPH, there is reason to be optimistic about bending the curve on PPH-associated morbidity and mortality globally. This research prioritisation exercise highlights the importance of caution and the urgent need for evidence to better understand the efficacy and safety of each tool and their effectiveness when used for PPH management in different settings. The results from the Delphi survey highlighted the need to generate knowledge and evidence on other important contextual factors (eg, risk of adverse events, usability, patient experience, among others) for each tool.

After the first Delphi survey, there were two tools that were excluded from this in-depth exploration. The Panicker suction^25^ was excluded because the published researchers on the device did not respond to multiple requests for information, and KII respondents expressed a general hesitancy to use relatively high pressures to create suction. The modified Bakri approach^26^ was excluded because it did not elicit much interest among phase 1 and 2 respondents, perhaps because it appeared less suitable for LMICs given the need for both a Bakri balloon and suction.

Across the different phases of this work, several interesting insights emerged. First, there was interest in exploring each of the four tools, with no clear front-runner. All four tools ranked within the top ten priority lists for further research in the second Delphi survey, underscoring the idea that the solution may not be a single tool.

Second, among the respondents in phase 2, there was greater interest in effectiveness research, and, in particular, interest in BEmONC settings. In contrast, phase 3 respondents placed greater priority on efficacy studies in CEmONC settings. The difference in participant compositions across phases may have contributed to this variation; in phase 2, the respondents represented a broader group of stakeholders while phase 3 respondents were primarily composed of OBGYNs and researchers. In phase 4, despite an overall lack of consensus on which tool may be most promising (though each tool warrants further exploration), there is overall agreement that research should begin in CEmONC settings where patient safeguards to ensure timely access to surgery and blood transfusion are in place.

We made a significant effort to include voices from stakeholders residing in LMICs and observed notable differences in opinion based on HIC vs LMIC settings. Participants in LMICs were more likely to prioritise effectiveness research while those from HICs prioritised efficacy and safety research. This may reflect the urgency felt by LMIC respondents, or a greater appreciation for contextual factors when moving from efficacy and safety to effectiveness in these settings. Alternatively, it may indicate a preference for pragmatic studies that reflect the real-world constraints of clinical practice.

It is also worth noting that there was greater interest in Jada by HIC respondents as compared with LMIC respondents. This may be a reflection of Jada’s FDA approval or greater awareness of the device in HIC settings, or perceptions among LMIC respondents that insertion processes may be challenging. XSTAT and Celox are both tools that require uterine packing, which has been regarded as controversial and not consistently effective, which may explain the relative hesitancy expressed about them. However, among participants in our phase 3 midwives-only focus group, there was an expressed preference for tools that could be inserted manually with the hands, such as CELOX. In phase 4 results, LMIC respondents tended to consistently rank Celox-related research questions higher than HIC respondents, perhaps suggesting a higher level of comfort and familiarity with uterine packing than device insertion, particularly in the hands of midwives.

For each of these tools, a need to examine patient experiences was identified, as well as provider usability and acceptability. This is particularly important if the goal is integration into WHO guidelines. Proactive efforts should be made to avoid divergent results or challenges with uptake, particularly when tools are rolled out in different settings and by different physician or midwife-led provider teams. Along these lines, determining whether these tools are acceptable, feasible and usable in the hands of midwives in LMICs is critical, highlighting the need for multidisciplinary research and training that brings together physicians and midwives in these contexts.

A strength of this study was that we were able to engage a wide range of stakeholders in the prioritisation process with a concerted effort to distinguish the views of LMIC and HIC professionals, as well as to understand the differing views by profession and cadre. However, there are important limitations to consider when interpreting the study results. First, the initial survey was sent to over 500 participants and had a low response rate of 13%. This was not surprising given our purposefully broad distribution, but our intent was to invite a wide range of respondents who might not usually be surveyed or provide input. Given the high proportion of respondents who were healthcare providers from LMICs, we think this achieved that goal (see onlinesupplemental files 5^6^). Second, the wording and discussion around Jada assumed a modified Jada device for an LMIC setting, which the company has indicated is in development and would be lower cost and reusable although the timeline is unclear for this prototype. Third, the final survey information was collected from a relatively small and purposive sample which may introduce bias in the results. While this approach has been used previously in similar research prioritisation studies and is generally accepted, it warrants caution when interpreting the results.^27^ Additionally, we received limited participation from LMIC midwives in our expert convening, and those who did participate may have been hesitant to openly express beliefs due to well-entrenched hierarchies in clinical practice and global health. Similarly, the response to both Delphi surveys may have been dominated by individuals working in high-resourced settings (academic hospitals) in both HIC and LMICs, which could have influenced their opinions and perspectives. We acknowledge that our inability to offer compensation for participation may have impeded broad participation, particularly from LMIC healthcare workers, where a donation of time is not possible for a host of reasons. Future research related to emerging PPH tools should include diverse provider cadres in order to build a coordinated and collaborative research agenda that engages both obstetricians and midwives. Finally, this work highlights the need to align our language when describing the timing of use for these tools, which we acknowledge created some confusion. Specifically, we differentiated use for refractory PPH (when all other modalities have been exhausted, prior to transfer to a higher level of care or the operating theatre) versus use after failed first response bundle (IV, TXA, massage, uterotonic) in an attempt to differentiate between an early or later use in the care pathway.^23^ We recognise that the field has not reached full consensus on this nomenclature, and thus the terminology may have been subject to interpretation which may have influenced results. The ongoing E-MOTIVE study may provide further insights into the complexities of this issue.^28^

## Conclusion

There are several promising suction and sponge tools for managing PPH that are on the horizon which may have the potential to reduce the global burden of this potentially deadly obstetric complication. With no single preferred tool among stakeholders, there is a need to better understand efficacy and safety, effectiveness and contextual implementation factors that are critical to the success of each of the emerging tools. A coordinated research agenda and collaborative research community that prioritises the needs of the women and intended end-user providers most impacted by PPH is urgently needed. This report aims to provide the inputs needed to accelerate this process and coordinate research on priority tools, topics and settings.

## Supplementary material

10.1136/bmjph-2023-000113 online supplemental file 1

10.1136/bmjph-2023-000113 online supplemental file 2

10.1136/bmjph-2023-000113 online supplemental file 3

10.1136/bmjph-2023-000113 online supplemental file 4

10.1136/bmjph-2023-000113 online supplemental file 5

10.1136/bmjph-2023-000113 online supplemental file 6

10.1136/bmjph-2023-000113 online supplemental file 7

## Data Availability

Data are available on reasonable request.
