# Supplementary material for: Global research priorities for intrauterine suction and sponge tools for postpartum haemorrhage management in low-income and middle-income countries: a modified Delphi approach
Source: BMJ Public Health. 2024 May 30;2(1):e000113. doi: 10.1136/bmjph-2023-000113 (PMC11812741; doi:10.1136/bmjph-2023-000113)
Supplement: online supplemental file 1 [file bmjph-2-1-s001.pdf]

Emerging PPH tools | Research Prioritization

There are a number of new non-balloon intrauterine tools currently being used and investigated for PPH management, such as suction and sponge products. With the overall goal of developing a research prioritization map that will enhance collaborative research regarding these emerging tools in low- and middle-income countries (LMICs), this survey aims to elicit your input on the potential for such products, what research data you would want to see generated, and what key considerations need to be taken into account in order to move the field forward.

Participation in this survey is voluntary. I acknowledge that I have received information about the purpose of this research and the study team. I understand that the answers I provide will remain anonymous and confidential. By providing my consent here, I agree to participate.	◯ Yes, I consent to participate.<br>◯ No, I do not consent to participate.

Thank you.
If you wish to complete the survey, please go back and provide consent.

SECTION A. DEMOGRAPHIC INFORMATION

Before we start, we would like to gather some of your information. We will summarize these data to describe our respondent population in our survey synthesis.

What is your profession? Please check all that apply.	☐ Healthcare provider<br>☐ Researcher<br>☐ Private sector/Industry<br>☐ Policy/Government<br>☐ Philanthropy/Foundation<br>☐ Implementor<br>☐ Other
If you are a healthcare provider, please specify:	◯ OB/GYN<br>◯ Other physician/clinical officer<br>◯ Midwife<br>◯ Nurse<br>◯ Other
If you are a healthcare provider, are you involved in teaching other clinicians either in pre-service or in-service (i.e., a clinician educator)?	◯ Yes<br>◯ No
If other profession, please specify:	______________________________
How many years of experience do you have in obstetrics, maternal health, and/or PPH (e.g., years of clinical experience and/or PPH research, etc.)?	☐ 0-5 years<br>☐ 6-10 years<br>☐ 11-20 years<br>☐ 20+ years
If you are a healthcare provider, approximately how many deliveries were you directly involved in over the last year?	◯ Less than 20<br>◯ Less than 100<br>◯ More than 100<br>◯ More than 300

If you are a healthcare provider, at what type of health facility do you primarily provide care?	◯ Primary health center<br>◯ District level hospital<br>◯ Tertiary referral hospital<br>◯ Private clinic or hospital<br>◯ Not-for-profit private/missionary hospital<br>◯ Academic center or teaching hospital<br>◯ Other
If other type of facility, please specify:	__________________________________
Do you reside in a low- or middle-income country (LMIC)?	◯ Yes<br>◯ No
If yes, where?	__________________________________
If no, do you conduct any PPH-related work in a LMIC?	◯ Yes<br>◯ No
In which LMICs do you primarily work with regards to obstetrics/PPH?	__________________________________

SECTION B. GENERAL QUESTIONS ABOUT PPH

We would like to explore your general perceptions about PPH management, as well as your initial perceptions about these emerging suction/sponge tools.

The figure below provides an overview of these products and our working synthesis of existing literature/research studies can be found HERE.

Some of these tools are built-for-purpose (meaning it's a tool designed specifically for PPH management), whereas other tools re-purpose existing supplies and equipment.

JADA device by Organon, intrauterine tubing loop with holes and inflatable cervical seal connected to wall suction (Purwosunu 2016, D'Alton 2020)

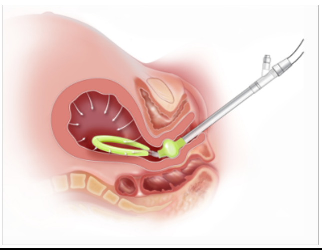

Modified XStat™ Mini **Sponge Tamponade** by Obstetrx, Inc. (Rodriguez 2017 & 2020)

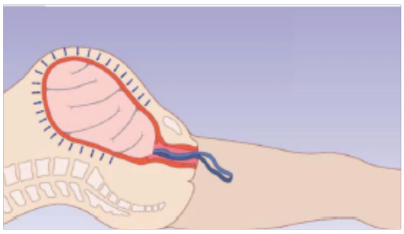

Celox™ by Medtrade, uterine packing with a chitosan-covered gauze tamponade (Schmid 2013, Dueckelmann 2019)

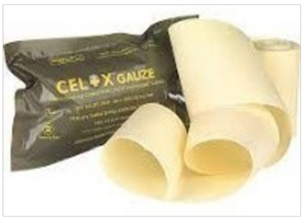

Stainless steel or plastic **cannula** with thick walled suction tube connected to vacuum device or pump (Panicker 2014 & 2017, Meena 2018)

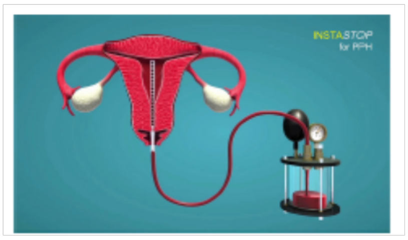

Levin suction tube connected to suction tubing and wall suction or MVA syringe (Hofmeyr 2019 & 2020, Cebekuhu 2021)

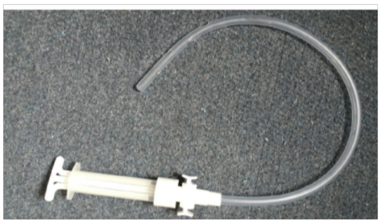

Vacuum-induced tamponade using a **modified balloon system** connected to vacuum device (Haslinger 2021)

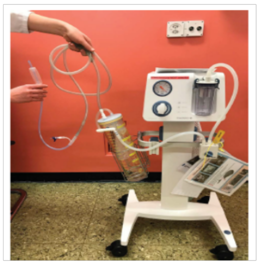

Have you heard about any of these intrauterine suction tools? This includes purpose-built devices such as Jada, and non-purpose built ones, such as Levin suction tube. This does *not* include uterine balloon tamponades (UBT).	◯ Yes<br>◯ No
Have you heard about sponge tamponades (xStat) or haemostatic gauze (Celox) for PPH management?	◯ Yes<br>◯ No
Have you heard of any other products based on the same principles?	◯ Yes<br>◯ No
If yes, please describe.	__________________________________
Where did you hear about these emerging tools? Please check all that apply.	☐ Used one clinically<br>☐ Used one in a clinical research context<br>☐ Read about a tool in a study, clinical trial, website, etc.<br>☐ Heard about a tool from an ongoing study from your professional networks<br>☐ Other
What tool or device did you use? Please check all that apply.	☐ Jada device<br>☐ xStat sponge<br>☐ Celox gauze<br>☐ Other suction device (e.g., suction tube) or non-balloon tamponade
How were you trained to use the Jada device? Please check all that apply.	☐ Trained by a peer or mentor clinician<br>☐ Trained as part of a research protocol<br>☐ In person formal training (e.g., device rep/developer)<br>☐ Video or online module<br>☐ Package insert<br>☐ Other training method
How were you trained to use xStat? Please check all that apply.	☐ Trained by a peer or mentor clinician<br>☐ Trained as part of a research protocol<br>☐ In person formal training (e.g., device rep/developer)<br>☐ Video or online module<br>☐ Package insert<br>☐ Other training method
How were you trained to use Celox? Please check all that apply.	☐ Trained by a peer or mentor clinician<br>☐ Trained as part of a research protocol<br>☐ In person formal training (e.g., product rep/developer)<br>☐ Video or online module<br>☐ Package insert<br>☐ Other training method

How were you trained to use this other tool? Please check all that apply.	☐ Trained by a peer or mentor clinician<br>☐ Trained as part of a research protocol<br>☐ In person formal training (e.g., product rep/developer)<br>☐ Video or online module<br>☐ Package insert<br>☐ Other training method
If other training method, please describe:	______________________________
How many times did you use the Jada device?	◯ less than 5 times<br>◯ 5-20 times<br>◯ more than 20 times
How many times did you use the xStat?	◯ less than 5 times<br>◯ 5-20 times<br>◯ more than 20 times
How many times did you use Celox?	◯ less than 5 times<br>◯ 5-20 times<br>◯ more than 20 times
How many times did you use this other device/tool?	◯ less than 5 times<br>◯ 5-20 times<br>◯ more than 20 times

If you have used a suction or sponge product clinically (in either practice or a research context), please rate your thoughts on feasibility, acceptability and usability.

	Strongly disagree	Disagree	Neutral	Agree	Strongly agree
I have the skills needed to use Jada.	◯	◯	◯	◯	◯
I have the other supplies and materials needed to use Jada.	◯	◯	◯	◯	◯
I felt confident using Jada.	◯	◯	◯	◯	◯
I found Jada cumbersome to use.	◯	◯	◯	◯	◯
I think I would use Jada in management of PPH.	◯	◯	◯	◯	◯
I have discussed with colleagues how well Jada works.	◯	◯	◯	◯	◯
I have the skills needed to use xStat.	◯	◯	◯	◯	◯
I have the other supplies and materials needed to use xStat.	◯	◯	◯	◯	◯
I felt confident using xStat.	◯	◯	◯	◯	◯
I found xStat cumbersome to use.	◯	◯	◯	◯	◯

I think I would use xStat in management of PPH.	○	○	○	○	○
I have discussed with colleagues how well xStat works.	○	○	○	○	○
I have the skills needed to use Celox.	○	○	○	○	○
I have the other supplies and materials needed to use Celox.	○	○	○	○	○
I felt confident using Celox.	○	○	○	○	○
I found Celox cumbersome to use.	○	○	○	○	○
I think I would use Celox in management of PPH.	○	○	○	○	○
I have discussed with colleagues how well Celox works.	○	○	○	○	○
I have the skills needed to use this other device/tool.	○	○	○	○	○
I have the other supplies and materials needed to use this other device/tool.	○	○	○	○	○
I felt confident using this other device/tool.	○	○	○	○	○
I found this other device/tool cumbersome to use.	○	○	○	○	○
I think I would use this other device/tool in management of PPH.	○	○	○	○	○
I have discussed with colleagues how well this other device/tool works.	○	○	○	○	○

Given the existing tools available for PPH management in LMICs such as uterotonics and the UBT, how important do you think additional non-balloon intrauterine devices or novel tools are needed?

- ○ Not important at all
- ○ A little important
- ○ Neutral
- ○ Somewhat important
- ○ Very important

What are the reasons why you think these novel tools are NOT important to pursue?

- ☐ Other existing tools, such as UBT and second-line uterotonics, are efficacious but require scale-up
- ☐ New tools will complicate the PPH treatment algorithm
- ☐ Concerns about these new tools' cost, scalability and sustainability in LMICs are significant (e.g., supply chain issues)
- ☐ Other reason

(Please check all that apply.)

What are the reasons why you think these novel tools are important to pursue?

- ☐ Conflicting data regarding efficacy of existing tools, such as UBT
- ☐ Lack of effectiveness of other treatment options in certain settings
- ☐ Lack of effectiveness in certain case presentations/patients
- ☐ Current tools/devices don't meet my needs (e.g., challenging to use, inadequate training)
- ☐ Convinced by early data that these tools have potential to save lives
- ☐ Other reason

(Please check all that apply.)

Please summarize any additional reasons for why you feel these emerging tools are or are not needed.

____

When you think about PPH treatment in your setting, what is the biggest challenge that may be overcome with a new tool like this? Please select the most important challenge to you.

- ◯ Inability to manage PPH alone or with limited personnel
- ◯ Difficulty with other available options (e.g., UBT)
- ◯ Lack of effective or accessible pharmacologic treatments
- ◯ Inability to control bleeding while operating theater is mobilized
- ◯ Inability to stabilize patient prior to referral
- ◯ Inability to monitor blood loss
- ◯ Other challenge

If other challenge, please describe:

____

When making a decision about introducing such a new tool in a LMIC setting, please rank the questions that must be answered in order of importance, with 1 being the highest priority,

	1 (highest priority)	2	3	4	5 (lowest priority)
How does it impact PPH clinical outcomes in a highly controlled research environment (efficacy & safety)?	◯	◯	◯	◯	◯
How does it impact PPH clinical outcomes in practice/real-world settings (effectiveness)?	◯	◯	◯	◯	◯
What is the patient experience, including pain, discomfort (feasibility/acceptability)?	◯	◯	◯	◯	◯
What is the provider experience, including ease of use (feasibility/acceptability)?	◯	◯	◯	◯	◯

What other factors should be considered, such as cost, training/skill retention, supply chain, scalability (implementation research)? ○ ○ ○ ○ ○

SECTION C. EFFICACY RESEARCH

This next section will explore your thoughts on critical research questions related to the tool's ability to produce expected results under ideal, highly controlled circumstances (efficacy).

At what LMIC health system level should this research be prioritized?

- ○ Basic Emergency Obstetric & Neonatal Care (BEmONC) facilities only
- ○ Comprehensive Emergency Obstetric & Neonatal Care (CEmONC) facilities only
- ○ Either BEmONC or CEmONC facilities
- ○ Academic center or teaching hospital
- ○ Facilities with intensive care only
- ○ Other

If other, please describe. __________________________________

What are essential health system pre-conditions that should be met for any setting conducting research trials including these new tools? Check all that apply.

- ☐ Primary PPH treatment protocol (use of uterotonics, IV, uterotonics, TXA, massage) is offered as standard of care
- ☐ Other causes of PPH can be excluded (retained/abnormal placenta, trauma)
- ☐ Maternal monitoring and prompt identification of deterioration
- ☐ Availability of wall suction/electricity
- ☐ 24-hour availability of anesthesia and surgical services
- ☐ Capacity for blood transfusion
- ☐ Ability to initiate PPH treatment and transfer patient to higher level of care if needed
- ☐ Other

If other, please describe. __________________________________

You are tasked with designing a study to see if one of these novel tools improves clinical outcome among women with PPH due to uterine atony after vaginal birth in a LMIC setting compared to a control group (efficacy study).

What primary device/tool would you prioritize testing?	◯ A purpose-built suction device, such as the Jada device<br>◯ A purpose-built sponge tamponade, such as the modified xStat sponge tamponade<br>◯ A non-purpose built tool, such as a suction Levin tube (Hofmyer)<br>◯ A non-purpose built tool, such as a vacuum-induced Bakri (Haslinger)<br>◯ A non-purpose built sponge device, such as Celox gauze<br>◯ Other
If other, please describe.	__________________________________
What would you compare it to?	☐ Uterine balloon tamponade<br>☐ Existing standard of care/PPH algorithm<br>☐ Other (e.g., another non-balloon device)
If other, please describe.	__________________________________
Where would you conduct this study (i.e., health system setting)?	◯ Primary health center<br>◯ District-level hospital<br>◯ Tertiary referral hospital<br>◯ Private clinic or hospital<br>◯ Not-for-profit private/missionary hospital<br>◯ Academic center or teaching hospital
In order to conduct this research safely in your selected LMIC setting, what PPH management elements would need to be strengthened or reinforced prior to beginning this study? Please check all that apply.	☐ Uterotonics for prevention<br>☐ Uterotonics for treatment<br>☐ Tranexamic acid (TXA) for treatment<br>☐ First response bundle (uterine massage, oxytocic drugs, TXA, IV fluids and examination & escalation)<br>☐ Second line uterotonics (e.g., ergometrine, misoprostol)<br>☐ Non-pneumatic anti-shock garment (NASG)<br>☐ Refractory bundle (compressive measures, NASG, uterine balloon tamponade)<br>☐ Functional referral system<br>☐ Respectful maternity care<br>☐ Teamwork and communication<br>☐ Not applicable, setting is already equipped/suitable<br>☐ Other
If other, please describe.	__________________________________
What provider cadre should be trained to insert the tool in this study? Please check all that apply.	☐ OBGYN<br>☐ Other physician/clinical officer<br>☐ Midwife<br>☐ Nurse<br>☐ Community health worker<br>☐ Emergency medical technician<br>☐ Traditional birth attendant

What is the primary (most important) clinical outcome you would measure? Select only one primary outcome.	◯ Measured blood loss >500ml<br>◯ Measured blood loss >1000ml<br>◯ Measured blood loss >1500ml<br>◯ Shock<br>◯ Coagulopathy<br>◯ Any organ dysfunction<br>◯ Maternal death<br>◯ Need for blood transfusion<br>◯ Laparotomy (sutures, hysterectomy)<br>◯ Use of any additional haemostatic intervention<br>◯ Transfer to a higher level of care<br>◯ Breastfeeding<br>◯ Adverse effects on mother (and baby if relevant)<br>◯ Time to bleeding cessation<br>◯ Post-procedural complication, e.g., infection<br>◯ Admission to intensive care unit<br>◯ Other not listed here<br>◯ A composite measure of several outcomes listed above<br>(List of outcomes adapted from Meher 2019 BJOG.)
If other outcome not listed, please describe.	__________________________________
What outcomes would be included in this composite primary outcome? Check all that you would include in a composite measure.	☐ Measured blood loss >500ml<br>☐ Measured blood loss >1000ml<br>☐ Measured blood loss >1500ml<br>☐ Shock<br>☐ Coagulopathy<br>☐ Any organ dysfunction<br>☐ Maternal death<br>☐ Need for blood transfusion<br>☐ Laparotomy (sutures, hysterectomy)<br>☐ Use of any additional haemostatic intervention<br>☐ Transfer to a higher level of care<br>☐ Breastfeeding<br>☐ Adverse effects on mother (and baby if relevant)<br>☐ Time to bleeding cessation<br>☐ Post-procedural complication, e.g., infection<br>☐ Admission to intensive care unit<br>☐ Other not listed here<br>☐ A composite measure of several outcomes listed above

What are up to 10 secondary outcomes you would measure?

- ☐ Measured blood loss >500ml
- ☐ Measured blood loss >1000ml
- ☐ Measured blood loss >1500ml
- ☐ Shock
- ☐ Coagulopathy
- ☐ Any organ dysfunction
- ☐ Maternal death
- ☐ Need for blood transfusion
- ☐ Laparotomy (sutures, hysterectomy)
- ☐ Use of any additional haemostatic intervention
- ☐ Transfer to a higher level of care
- ☐ Breastfeeding
- ☐ Adverse effects on mother (and baby if relevant)
- ☐ Time to bleeding cessation
- ☐ Post-procedural complication, e.g., infection
- ☐ Admission to intensive care unit
- ☐ Completed referral
- ☐ Provider perceptions (ease of use)
- ☐ Women's perceptions (pain, comfort)
- ☐ Other not listed here

(Select only ten.)

If other secondary outcome not listed, please describe.

____

Given what you know about these novel tools, AFTER which step in PPH management would you insert the device/tool?

- ◯ First dose oxytocin
- ◯ IV fluids
- ◯ TXA
- ◯ Uterine massage
- ◯ Manual sweep/check for retained tissue
- ◯ First check for tears
- ◯ Maximize oxytocin dose
- ◯ Second-line uterotonic for treatment
- ◯ Second check for tears
- ◯ Failed placement of other device, e.g., UBT
- ◯ Decision to refer or move to operating theater
- ◯ I don't know

(Please select the clinical task after which you would use the device.)

SECTION D. RESEARCH BEYOND EFFICACY

This next section will explore your thoughts on critical research questions related to tool characteristics, effectiveness in real-world settings, and the optimal conditions for implementation. This includes, for example, questions related to feasibility, acceptability and usability from the perspectives of providers, women and the health system.

What elements or characteristics of intrauterine devices/tools should be further explored in order to optimize or better understand their use? Please rank the following topics with 1 being the most important.

	1 (most important)	2	3	4	5	6 (least important)

Adverse clinical outcomes (infections, perforation, misplacement)	○	○	○	○	○	○
Length of time the device remains in place for treatment	○	○	○	○	○	○
One-time use vs. reusability	○	○	○	○	○	○
Minimum suction required for effect	○	○	○	○	○	○
Standard procedures recommended with use (e.g., prophylactic antibiotic prescription, ultrasound to guide placement)	○	○	○	○	○	○
Prophylactic placement to prevent PPH among women with elevated risk (e.g., multigravida, history of PPH)	○	○	○	○	○	○

Please add any other tool-related considerations you feel are important to consider that are not listed above. ____

From providers' perspectives, what are priority topics to explore regarding introduction of these novel tools? Please rank the following topics with 1 being the most important.

	1 (most important)	2	3	4 (least important)
Feasibility, ease of insertion and/or use	○	○	○	○
Acceptability, resistance/acceptance to change clinical practice	○	○	○	○
Suitability of use by non-physician providers (e.g., usability by midwives or other frontline workers)	○	○	○	○
Effective training modalities to ensure and maintain competency (e.g., effective training standards, guidelines, certification and skills maintenance program)	○	○	○	○

Please add any other provider-related considerations you feel are important to consider that are not listed above. ____

From women's perspectives, what are priority topics to explore regarding introduction of these novel tools? Please rank the following topics with 1 being the most important.

	1 (most important)	2	3	4	5	6 (least important)
Level of pain with placement and removal	○	○	○	○	○	○
Level of pain throughout duration of treatment	○	○	○	○	○	○
Length of insertion/treatment	○	○	○	○	○	○
Acceptability, resistance/acceptance to device use	○	○	○	○	○	○
Type of provider who should insert the device	○	○	○	○	○	○
Overall experience of care, including respectful care and communication	○	○	○	○	○	○

Please add any other patient-related considerations you feel are important to consider that are not listed above. ______________________________

What health system factors should be further explored prior to implementation in order to optimize or better understand their adoption and uptake (scalability)? Please rank the following topics with 1 being the most important.

	1 (most important)	2	3	4	5	6 (least important)
Product cost and implementation cost	○	○	○	○	○	○
Cost-effectiveness, including cost savings for averting subsequent care (e.g., surgery, transfusion, length of ICU stay)	○	○	○	○	○	○
Options for use in settings which lack electricity	○	○	○	○	○	○
Options for use during referral/transport to higher levels of care	○	○	○	○	○	○
Modifications of current PPH management algorithms to optimize integration and introduction	○	○	○	○	○	○

Requirements for supply chain and device maintenance (e.g., storage conditions, cleaning/reuse) ◯ ◯ ◯ ◯ ◯ ◯

Please add any other system-related considerations you feel are important to consider that are not listed above. __________________________________

Please share any ethical considerations you would like to highlight related to conducting this type of research. __________________________________

In your opinion, what cadre of provider could safely insert each of these products (if proven effective)?

	Jada device	xStat sponge	Celox gauze	Other suction device (e.g., suction tube) or non-balloon tamponade
OBGYN	☐	☐	☐	☐
Other physician/clinical officer	☐	☐	☐	☐
Midwife	☐	☐	☐	☐
Nurse	☐	☐	☐	☐
Community health worker	☐	☐	☐	☐
Emergency medical technician	☐	☐	☐	☐
Traditional birth attendant	☐	☐	☐	☐

Based on what you know about these emerging suction/sponge tools, if you had access to them in your facility, would you use one?

◯ Yes
◯ No

Which product would you use? Check only one.

◯ Jada device
◯ xStat sponge
◯ Other suction device (e.g., suction tube)
◯ Celox gauze
◯ Other non-balloon tamponade

Why did you select [consider_use]? __________________________________

If you are a policy maker or implementer, what evidence would you need to recommend introduction of one of these new intrauterine tools to control PPH? __________________________________

We appreciate you completing this survey! We are seeking input from various PPH experts and stakeholders representing academia, clinical practice, industry/private sector, policy and philanthropy and are committed to ensuring dissemination to you and the broader PPH community. We hope this work can inform collaborative and coordinated efforts to move the field forward.

If you would like to receive a synthesis of our study findings (e.g., invitation to webinar or written report), please fill out the optional information below.

Name (optional)

____
(Please note that we will keep your answers confidential and will not share your personal information with anyone outside the research team.)

Email address (optional)

____
(Please note that we will keep your answers confidential and will not share your personal information with anyone outside the research team.)

Can study staff reach out to you if we would like to further discuss your answers or this project?

◯ Yes
◯ No

Any other comments or considerations you would like to share that was not covered in the sections above?

____

End of survey.

THANK YOU!
