# Supplementary material for: Global research priorities for intrauterine suction and sponge tools for postpartum haemorrhage management in low-income and middle-income countries: a modified Delphi approach
Source: BMJ Public Health. 2024 May 30;2(1):e000113. doi: 10.1136/bmjph-2023-000113 (PMC11812741; doi:10.1136/bmjph-2023-000113)
Supplement: online supplemental file 2 [file bmjph-2-1-s002.pdf]

Emerging PPH tools| Product Profile Survey

One of the ways device and drug manufacturers formulate customer or end-user requirements is by defining a minimally viable product, as well as a desired product profile.

In this survey, we will ask you to choose whether the following characteristics would be essential or desirable when using this envisioned product to treat PPH in your setting.

Participation in this survey is voluntary. I acknowledge that I have received information about the purpose of this research and the study team. I understand that the answers I provide will remain anonymous and confidential. By providing my consent here, I agree to participate.

- ◯ Yes, I consent to participate.
- ◯ No, I do not consent to participate.

Thank you.
If you wish to complete the survey, please provide consent.

Before we start, please answer some basic demographic information.

What is your primary profession or work environment? Select only one.

- ◯ Healthcare provider
- ◯ Hospital administrator
- ◯ Researcher
- ◯ Private sector/Industry
- ◯ Policy/Government
- ◯ Philanthropy/Foundation
- ◯ Program implementor
- ◯ Other

If you are a healthcare provider, please specify.

- ◯ OB/GYN
- ◯ Other physician/clinical officer
- ◯ Midwife
- ◯ Nurse
- ◯ Other

If you are a healthcare provider or hospital administrator, at what type of health facility do you primarily provide care?

- ◯ Primary health center
- ◯ District level hospital
- ◯ Tertiary referral hospital
- ◯ Private clinic or hospital
- ◯ Not-for-profit private/missionary hospital
- ◯ Academic center or teaching hospital
- ◯ Other

In what type of setting/context do you primarily work with regards to obstetrics/PPH?

- ◯ High-resourced setting where supplies, personnel and supply chain are adequate
- ◯ Low-resourced setting where supplies, personnel and supply chain limitations are key considerations

---

PLEASE READ INSTRUCTIONS CAREFULLY.

For the questions below, please choose whether you think the proposed characteristic is:

absolutely essential for use in your setting, meaning that at the very minimum, the envisioned product must have this characteristic desirable in an ideal scenario, meaning that it would be nice to have, but is not essential not important for use in your setting   Please select no opinion if you are neutral.

Example: An essential characteristic of a novel drug would be that it can be stored at ambient temperature, but a desirable characteristic would be that it would be able to withstand high temperatures without any effect on stability. The essential characteristic would be necessary for adoption, while the desirable characteristic would make adoption more likely.

CLINICAL INDICATION: In your setting, when treating a woman with PPH, what patient profile must this product respond to (essential) and what would be desirable?

	Essential	Desirable	Not important	No opinion
PPH due to uterine atony following vaginal birth	◯	◯	◯	◯
PPH due to uterine atony following Cesarean delivery	◯	◯	◯	◯
Trauma to genital tract	◯	◯	◯	◯
Placental implantation abnormality	◯	◯	◯	◯
Coagulopathy	◯	◯	◯	◯

TIMING OF USE: In your setting, when treating a woman with PPH due to atony, when would use of such a product be essential and when would it be desirable?

	Essential	Desirable	Not important	No opinion
When heavy bleeding is first noted (e.g., before administration of first-line uterotonic)	◯	◯	◯	◯
When bleeding continues after initial response (e.g., after use of first response bundle)	◯	◯	◯	◯
When all other options to stop the bleeding have been exhausted	◯	◯	◯	◯

EFFICACY/RISK TO PREVENT SURGICAL INTERVENTION: For you to consider adopting one of these products in your setting, how should its efficacy to prevent surgical intervention compare to your current standard of care to make adoption more likely?

	Essential	Desirable	Not important	No opinion
Works at least as well as current standard of care with no increased risk	◯	◯	◯	◯
Works at least as well as current standard of care with minimal increased risk	◯	◯	◯	◯
Works at least as well as current standard of care with moderate increased risk	◯	◯	◯	◯
Works better than current standard of care with no increased risk	◯	◯	◯	◯

EFFICACY/RISK TO PREVENT BLOOD LOSS: For you to consider adopting one of these products in your setting, how should its efficacy to prevent blood loss >1000ml compare to your current standard of care to make adoption more likely?

	Essential	Desirable	Not important	No opinion
Works at least as well as current standard of care with no increased risk	○	○	○	○
Works at least as well as current standard of care with minimal increased risk	○	○	○	○
Works at least as well as current standard of care with moderate increased risk	○	○	○	○
Works better than current standard of care with no increased risk	○	○	○	○

FACILITY TYPE: For you to consider adopting one of these products in your setting, in what type of facility must it be useable in (essential), and in what type of facility would it be ideally useable in (desirable)?

	Essential	Desirable	Not important	No opinion
Can be used in a primary health center	○	○	○	○
Can be used during transport or referral as a temporizing measure	○	○	○	○
Can be used in a district hospital	○	○	○	○
Can be used in a tertiary hospital	○	○	○	○
Can be used in a private clinic or hospital	○	○	○	○

PROVIDER CADRE: For you to consider adopting one of these products in your setting, what is the provider type that must be able to use it (essential), and what is the cadre you would like to see it used by (desirable)?

	Essential	Desirable	Not important	No opinion
Can be inserted by an OBGYN	○	○	○	○
Can be inserted by a medical officer or clinical officer	○	○	○	○
Can be inserted by a midwife	○	○	○	○
Can be inserted by a nurse	○	○	○	○
Can be inserted by a community health worker	○	○	○	○

	Essential	Desirable	Not important	No opinion
Can be used by an emergency care technician (e.g., before/during transfer)	○	○	○	○
Can be inserted by a traditional birth attendant	○	○	○	○

TRAINING MECHANISM: For you to consider adopting one of these products in your setting, what training mechanism would be essential or desirable to ensure provider competency?

	Essential	Desirable	Not important	No opinion
Live online training	○	○	○	○
Asynchronous online training (e.g., pre-recorded webinar/video)	○	○	○	○
In person training with simulation/model training	○	○	○	○
In person training with ongoing proctoring or mentoring	○	○	○	○

PROVIDER COMPETENCY: For you to consider adopting one of these products in your setting, what is the number of successful insertions that would be essential or desirable to ensure provider competency (e.g., able to perform independently)?

	Essential	Desirable	Not important	No opinion
Competent after completing at least 1 insertion	○	○	○	○
Competent after completing 2 to 5 insertions	○	○	○	○
Competent after completing more than 5 insertions	○	○	○	○

EASE OF USE: For you to consider adopting one of these products in your settings, what ease of use characteristics would be essential or desirable?

	Essential	Desirable	Not important	No opinion
Ability to insert with one hand	○	○	○	○
Ability for a single provider to use/insert	○	○	○	○
Ability to quickly insert (i.e., less than 2 minutes)	○	○	○	○
Little to no assembly required	○	○	○	○

TREATMENT DURATION: For you to consider adopting one of these products in your setting, what is the essential or desirable treatment duration that would make adoption more appealing?

	Essential	Desirable	Not important	No opinion

Duration of treatment is no longer than 24 hours	○	○	○	○
Duration of treatment is no longer than 6 hours	○	○	○	○
Duration of treatment is comparable to current standard of care	○	○	○	○
Duration of treatment is shorter than current standard of care	○	○	○	○

PATIENT EXPERIENCE: For you to consider adopting one of these products in your setting, what should the essential and desirable/acceptable patient experience be (assuming the product is effective)?

	Essential	Desirable/Acceptable	Not important	No opinion
Experience is similar to standard care, with no added discomforts	○	○	○	○
Experience is in some way better than standard care	○	○	○	○
Experience is in some way worse than standard care	○	○	○	○

REUSABILITY: For you to consider adopting one of these products in your setting, what characteristics related to reusability would be essential or desirable?

	Essential	Desirable	Not important	No opinion
Entire product can be sterilized and reused	○	○	○	○
Parts of the product can be sterilized and reused	○	○	○	○
Product cannot be reused	○	○	○	○

STORAGE CHARACTERISTICS: For you to consider adopting one of these products in your setting, what characteristics related to storage/handling would be essential or desirable?

	Essential	Desirable	Not important	No opinion
Storage at ambient temperature	○	○	○	○
Storage below ambient temperature (i.e., requires a cold chain)	○	○	○	○
Ability to withstand high ambient temperatures (e.g., hot, humid climates)	○	○	○	○

PRODUCT COST: For you to consider adopting one of these products in your setting, what are essential and desirable cost considerations?

	Essential	Desirable	Not important	No opinion
Less than $10 USD/patient	◯	◯	◯	◯
Less than $50 USD/ patient	◯	◯	◯	◯
Less than $100 USD/patient	◯	◯	◯	◯
Less than $500 USD/patient	◯	◯	◯	◯

REGULATORY CONSIDERATIONS: For you to consider adopting one of these products in your setting, what regulatory considerations and approvals must be in place (essential) or would ideally be in place (desirable) before use?

	Essential	Desirable	Not important	No opinion
Has regulatory approval for the indication of PPH	◯	◯	◯	◯
Has regulatory approval for other clinical indications/uses; can be used "off-label" for PPH	◯	◯	◯	◯
No regulatory approvals are needed at all	◯	◯	◯	◯

In the questions above, you told us what you would like to see as essential and desirable product characteristics across several categories. Since a product is unlikely to address every need, we would like to know which you feel are most important.

Please select the top 6 MOST important considerations in product optimization/development.

- ☐ Clinical indication (type of PPH)
- ☐ Timing of use during PPH management
- ☐ Efficacy/risk - prevention of surgical intervention
- ☐ Efficacy/risk - prevention of blood loss >1000ml
- ☐ Facility type
- ☐ Provider cadre
- ☐ Training mechanism
- ☐ Provider proficiency
- ☐ Treatment duration
- ☐ Patient experience
- ☐ Reusability
- ☐ Storage characteristics
- ☐ Product cost
- ☐ Regulatory considerations

If there are other considerations that are not included above but that you feel are essential characteristics of this type of product, please describe.

____

Now, please select up to 6 considerations that are LEAST important in the development/optimization of these products.

- ☐ Clinical indication (type of PPH)
- ☐ Timing of use during PPH management
- ☐ Efficacy/risk - prevention of surgical intervention
- ☐ Efficacy/risk - prevention of blood loss >1000ml
- ☐ Facility type
- ☐ Provider cadre
- ☐ Training mechanism
- ☐ Provider proficiency
- ☐ Treatment duration
- ☐ Patient experience
- ☐ Reusability
- ☐ Storage characteristics
- ☐ Product cost
- ☐ Regulatory considerations

Please share any ethical considerations you would like to highlight related to these types of products.

____

We appreciate you completing this survey! We are seeking input from various PPH experts and stakeholders representing academia, clinical practice, industry/private sector, policy and philanthropy and are committed to ensuring dissemination to you and the broader PPH community. We hope this work can inform collaborative and coordinated efforts to move the field forward.

If you would like to receive a synthesis of our study findings (e.g., invitation to webinar or written report), please fill out the optional information below.

Name (optional)

____
(Please note that we will keep your answers confidential and will not share your personal information with anyone outside the research team.)

Email address (optional)

____
(Please note that we will keep your answers confidential and will not share your personal information with anyone outside the research team.)

Are there any other additional comments or considerations you would like to share?

____

End of survey.
THANK YOU!
