# Supplementary material for: Global research priorities for intrauterine suction and sponge tools for postpartum haemorrhage management in low-income and middle-income countries: a modified Delphi approach
Source: BMJ Public Health. 2024 May 30;2(1):e000113. doi: 10.1136/bmjph-2023-000113 (PMC11812741; doi:10.1136/bmjph-2023-000113)
Supplement: online supplemental file 3 [file bmjph-2-1-s003.pdf]

# Emerging suction & sponge tools for PPH | Post-convening survey

Thank you again for participating in our expert convening meeting on Thursday, July 21st to further discuss emerging suction and sponge tools for PPH management in LMICs. As discussed, our goal is to develop a research roadmap/priority list that will enhance coordinated and collaborative research. This survey synthesizes key research areas identified during this process and seeks your input to refine and prioritize specific research questions.

If you are interested in continued collaboration as we develop the manuscript related to this process, please let us know at the end of the survey (Section D), and we will reach out to you with next steps.

| | |
|---|---|
| Participation in this survey is voluntary. I acknowledge that I have received information about the purpose of this research and the study team. I understand that the answers I provide will remain anonymous. By providing my consent here, I agree to participate. | ◯ Yes, I consent to participate.<br>◯ No, I do not consent to participate. |

If you wish to complete the survey, please go back and provide consent.

## SECTION A. DEMOGRAPHIC INFORMATION

| | |
|---|---|
| What is your primary profession? | ☐ Doctor/specialist provider<br>☐ Midwife/nurse<br>☐ Researcher<br>☐ Private sector/Industry<br>☐ Policy/Government<br>☐ Philanthropy/Foundation<br>☐ Implementor<br>☐ Other<br>(Please check only one.) |
| Do you reside in a low- or middle-income country (LMIC)? | ◯ Yes<br>◯ No |
| If no, do you conduct any PPH-related work in a LMIC? | ◯ Yes<br>◯ No |
| How many years of experience do you have in obstetrics, maternal health, and/or PPH (e.g., years of clinical experience and/or PPH research, etc.)? | ◯ 0-5 years<br>◯ 6-10 years<br>◯ 11-20 years<br>◯ 20+ years |
| Have you previously been involved in research related to any emergency obstetric condition? | ◯ Yes, in both high- and low-resourced settings<br>◯ Yes, in high-resourced settings only<br>◯ Yes, in low-resourced settings only<br>◯ No |

## SECTION B. RESEARCH PRIORITY AREAS BY DEVICE.

During the meeting, we focused on four tools - the Jada device, Levin gastric tube, XSTAT sponge, and Celox gauze (see figure). For each section below, we will share the summary of the group's collective responses regarding the research setting where priority research should be conducted for each of the four devices. We will then present a list of potential research questions that were identified in the small breakout groups. For each draft research question, you will be asked to keep, modify or remove it - followed by a series of follow-up questions depending on your answer.

**JADA device** by Organon, intrauterine tubing loop with holes and inflatable cervical seal connected to wall suction (Purwosunu 2016, D'Alton 2020)

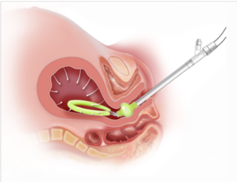

Modified XStat™ Mini **Sponge Tamponade** by Obstetrx, Inc. (Rodriguez 2017 & 2020)

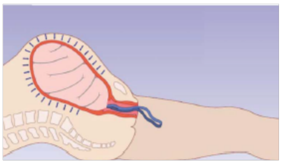

**Celox™** by Medtrade, uterine packing with a chitosan-covered gauze (Schmid 2013, Carles 2017 Dueckelmann 2019, Biele 2022)

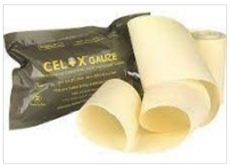

**Levin gastric tube,** wide-bore suction tube connected by suction tubing to wall suction, pump or MVA syringe (Hofmeyr 2019 & 2020, Cebekuhu 2021)

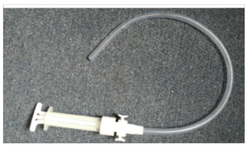

Before we start, this is how we define some of terms we use:

1. Adequately resourced CEmONC setting: a facility with access to refractory PPH care and surgical management
2. Adequately resourced BEmONC setting: a facility with access to first reponse bundle and ability to refer out
3. Standard of care: existing response to PPH management which may or may not include provision of UBT (in other words, training to introduce UBT would not be necessary to conduct the proposed study)
4. First response bundle: consists of uterine massage, oxytocic drugs, TXA, IV fluids and examination & escalation per the E-MOTIVE project
5. Refractory PPH: bleeding not responsive to the first response bundle and other available measures, such as uterine massage, maximal uterotonic dosage, repair of tears, and removal of retained tissue
6. Efficacy research: a study that is executed in a well-controlled research setting (e.g., individually randomized controlled trial)
7. Effectiveness research: a study that is executed in a real-world setting (e.g., cluster randomized controlled trial)

## JADA DEVICE

When asked what type of priority research should be conducted for the Jada device, here is a summary of your collective responses.

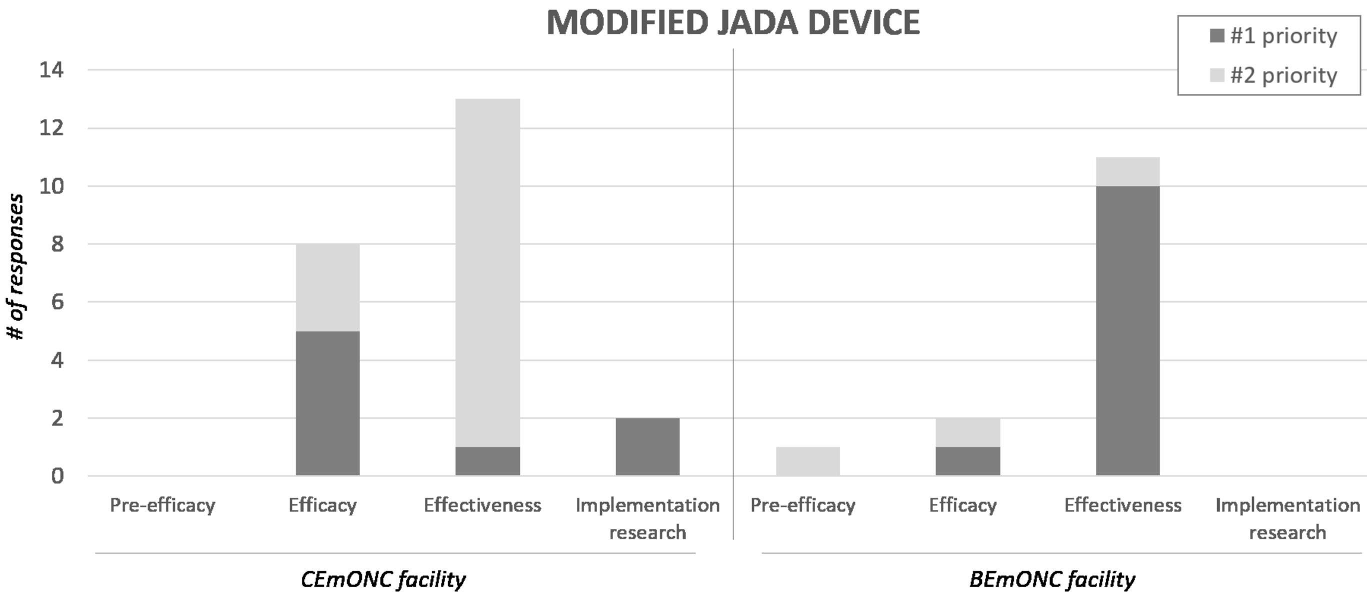

Within the small group that discussed Jada, the following research questions were identified, under the condition that a more affordable modified device was available in LMIC settings. For each proposed question, please select whether you would keep, modify or remove it.

Please "keep" questions that you feel are important to address in the next 5 years.

## EFFICACY & SAFETY | CEmONC SETTING
## Modified Jada device

| | |
|---|---|
| In an adequately resourced CEmONC setting in an LMIC, what is the efficacy and safety of the modified Jada device compared to standard of care in the reduction of PPH-related maternal morbidity and mortality among women who experience refractory PPH due to uterine atony?<br><br>This study would be conducted in a highly controlled research LMIC setting to test whether Jada works to control refractory PPH due to atony. | ◯ Keep<br>◯ Keep with modifications<br>◯ Remove |
| Please enter your suggested modification. | ______________________________ |
| What types of births/deliveries should this research include? | ◯ All births (vaginal or CS)<br>◯ Vaginal delivery only<br>◯ Vaginal delivery with no prior CS<br>◯ Only CS<br>(Select only one.) |
| Please rate the priority of this research question (as written or your suggested modification). | ◯ High priority<br>◯ Moderate priority<br>◯ Low priority |
| In an adequately resourced CEmONC setting in an LMIC, what is the efficacy and safety of the modified Jada device compared to standard of care in the reduction of PPH-related maternal morbidity and mortality among women who experience PPH due to atony who do not respond to the first response bundle?<br><br>This study would be conducted in a highly controlled research LMIC setting to test whether Jada works to control PPH after the first response bundle, including uterine massage, oxytocic drugs, TXA, IV fluids and examination & escalation. | ◯ Keep<br>◯ Keep with modifications<br>◯ Remove |
| Please enter your suggested modification. | ______________________________ |
| What types of births/deliveries should this research include? | ◯ All births (vaginal or CS)<br>◯ Vaginal delivery only<br>◯ Vaginal delivery with no prior CS<br>◯ Only CS<br>(Select only one.) |

| | |
|---|---|
| Please rate the priority of this research question (as written or your suggested modification). | ◯ High priority<br>◯ Moderate priority<br>◯ Low priority |
| Any additional research questions, comments or feedback you would like to add regarding research related to efficacy of the modified Jada device in CEmONC settings? | __________________________________ |

**EFFECTIVENESS | CEmONC SETTING**

**Modified Jada device**

| | |
|---|---|
| In adequately resourced CEmONC setting in an LMIC, what is the effectiveness of using the modified Jada device compared to standard of care in the reduction of PPH-related maternal morbidity and mortality among women who experience refractory PPH due to uterine atony?<br><br>This study would be conducted in a real-world LMIC setting to test whether Jada works to control refractory PPH due to atony. | ◯ Keep<br>◯ Keep with modifications<br>◯ Remove |
| Please enter your suggested modification. | __________________________________ |
| What types of births/deliveries should this research include? | ◯ All births (vaginal or CS)<br>◯ Vaginal delivery only<br>◯ Vaginal delivery with no prior CS<br>◯ Only CS<br>(Select only one.) |
| Please rate the priority of this research question (as written or your suggested modification). | ◯ High priority<br>◯ Moderate priority<br>◯ Low priority |
| In adequately resourced CEmONC setting in an LMIC, what is the effectiveness of using the modified Jada device compared to standard of care in the reduction of PPH-related maternal morbidity and mortality among women who experience PPH due to atony who do not respond to the first response bundle?<br><br>This study would be conducted in a real-world LMIC setting to test whether Jada works to control PPH after the first response bundle, including uterine massage, oxytocic drugs, TXA, IV fluids and examination & escalation. | ◯ Keep<br>◯ Keep with modifications<br>◯ Remove |
| Please enter your suggested modification. | __________________________________ |
| What types of births/deliveries should this research include? | ◯ All births (vaginal or CS)<br>◯ Vaginal delivery only<br>◯ Vaginal delivery with no prior CS<br>◯ Only CS<br>(Select only one.) |

| | |
|---|---|
| Please rate the priority of this research question (as written or your suggested modification). | ◯ High priority<br>◯ Moderate priority<br>◯ Low priority |
| Any additional research questions, comments or feedback you would like to add regarding research related to effectiveness of the modified Jada device in CEmONC settings? | __________________________________ |

## RESEARCH at BEmONC SETTINGS

## Modified Jada device

| | |
|---|---|
| Do you believe that any efficacy or effectiveness studies related to the modified Jada device should be conducted at BEmONC settings in an LMIC? | ◯ Yes<br>◯ Yes, but only after its efficacy has been evaluated in a CEmONC setting<br>◯ Yes, but only after its efficacy and effectiveness have been evaluated at a CEmONC setting<br>◯ No<br>◯ Not sure |
| In adequately resourced BEmONC settings in an LMIC, what is the effectiveness of using the modified Jada device compared to standard of care in the reduction of PPH-related maternal morbidity and mortality among women who experience refractory PPH prior to referral?<br><br>This study would be conducted in a real-world LMIC setting to test whether Jada (administered by a nurse or midwife) works to improve PPH outcomes, such as stabilize a woman who requires transfer to a higher level of care for PPH. | ◯ Keep<br>◯ Keep with modifications<br>◯ Remove |
| Please enter your suggested modification. | __________________________________ |
| Please rate the priority of this research question (as written or your suggested modification). | ◯ High priority<br>◯ Moderate priority<br>◯ Low priority |
| In an adequately resourced BEmONC settings in an LMIC, what is the effectiveness of using the modified Jada device compared to standard of care in the reduction of PPH-related maternal morbidity and mortality among women who experience PPH due to atony who do not respond to the first response bundle?<br><br>This study would be conducted in a real-world LMIC setting to test whether Jada (administered by a nurse or midwife) works to improve PPH outcomes, such as control PPH so that referral can be avoided. | ◯ Keep<br>◯ Keep with modifications<br>◯ Remove |
| Please enter your suggested modification. | __________________________________ |
| Please rate the priority of this research question (as written or your suggested modification). | ◯ High priority<br>◯ Moderate priority<br>◯ Low priority |

Any additional research questions, comments or feedback you would like to add regarding research related to the modified Jada device in BEmONC settings?

________________________________

If there are other research questions not included above that you feel are priority areas for the modified Jada device in either CEmONC or BEmONC settings, please include them here.

________________________________

## LEVIN GASTRIC TUBE

When asked what type of priority research should be conducted for the improvised Levin gastric tube, here is a summary of your collective responses.

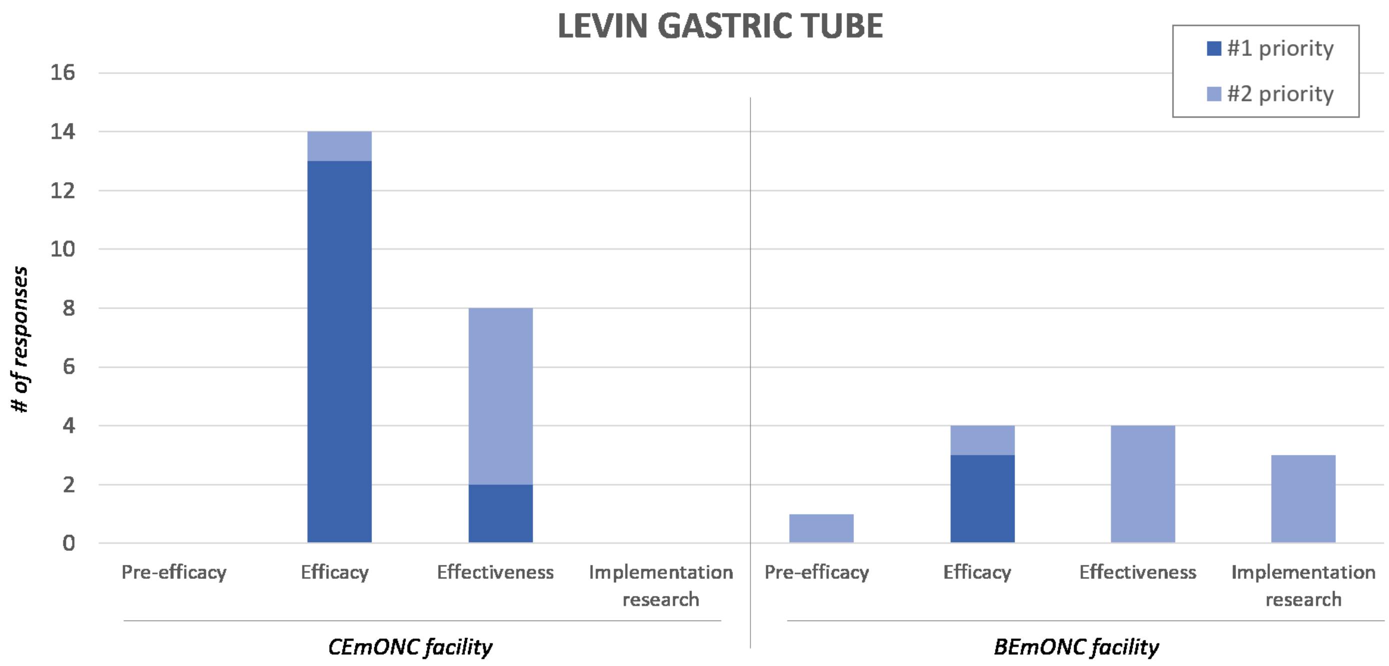


Within the small group that discussed the Levin gastric tube, the following research questions were identified for LMIC settings. For each proposed question, please select whether you would keep, modify or remove it.

Please "keep" questions that you feel are important to address in the next 5 years.

## EFFICACY & SAFETY | CEmONC SETTING
## Levin Gastric Tube

In an adequately resourced CEmONC setting in an LMIC, what is the efficacy and safety of the Levin gastric tube compared to standard of care in the reduction of PPH-related maternal morbidity and mortality among women who experience refractory PPH due to uterine atony?

This study would be conducted in a highly controlled research LMIC setting to test whether the Levin gastric tube works to control refractory PPH due to atony.

- ◯ Keep
- ◯ Keep with modifications
- ◯ Remove

Please enter your suggested modification. ______________________________________

What types of births/deliveries should this research include?
- ◯ All births (vaginal or CS)
- ◯ Vaginal delivery only
- ◯ Vaginal delivery with no prior CS
- ◯ Only CS

(Select only one.)

Please rate the priority of this research question (as written or your suggested modification).
- ◯ High priority
- ◯ Moderate priority
- ◯ Low priority

In an adequately resourced CEmONC setting in an LMIC, what is the efficacy and safety of the Levin gastric tube compared to standard of care in the reduction of PPH-related maternal morbidity and mortality among women who experience PPH due to atony who do not respond to the first response bundle?

This study would be conducted in a highly controlled research LMIC setting to test whether the Levin gastric tube works to control PPH after the first response bundle, including uterine massage, oxytocic drugs, TXA, IV fluids and examination & escalation.
- ◯ Keep
- ◯ Keep with modifications
- ◯ Remove

Please enter your suggested modification. ______________________________________

What types of births/deliveries should this research include?
- ◯ All births (vaginal or CS)
- ◯ Vaginal delivery only
- ◯ Vaginal delivery with no prior CS
- ◯ Only CS

(Select only one.)

Please rate the priority of this research question (as written or your suggested modification).
- ◯ High priority
- ◯ Moderate priority
- ◯ Low priority

Any additional research questions, comments or feedback you would like to add regarding research related to efficacy of the Levin gastric tube in CEmONC settings? ______________________________________

## EFFECTIVENESS| CEmONC SETTING
## Levin Gastric Tube

| | |
|---|---|
| In adequately resourced CEmONC settings in an LMIC, what is the effectiveness of using the Levin gastric tube compared to standard of care in the reduction of PPH-related maternal morbidity and mortality among women who experience refractory PPH due to uterine atony?<br><br>This study would be conducted in a real-world LMIC CEmONC setting to test whether the Levin gastric tube works to control refractory PPH due to atony. | ◯ Keep<br>◯ Keep with modifications<br>◯ Remove |
| Please enter your suggested modification. | ____________________________________ |
| What types of births/deliveries should this research include? | ◯ All births (vaginal or CS)<br>◯ Vaginal delivery only<br>◯ Vaginal delivery with no prior CS<br>◯ Only CS<br>(Select only one.) |
| Please rate the priority of this research question (as written or your suggested modification). | ◯ High priority<br>◯ Moderate priority<br>◯ Low priority |
| In adequately resourced CEmONC setting in an LMIC, what is the effectiveness of using the Levin gastric tube compared to standard of care in the reduction of PPH-related maternal morbidity and mortality among women who experience PPH due to atony who do not respond to the first response bundle?<br><br>This study would be conducted in a real-world LMIC setting to test whether the Levin gastric tube works to control PPH after the first response bundle, including uterine massage, oxytocic drugs, TXA, IV fluids and examination & escalation. | ◯ Keep<br>◯ Keep with modifications<br>◯ Remove |
| Please enter your suggested modification. | ____________________________________ |
| What types of births/deliveries should this research include? | ◯ All births (vaginal or CS)<br>◯ Vaginal delivery only<br>◯ Vaginal delivery with no prior CS<br>◯ Only CS<br>(Select only one.) |
| Please rate the priority of this research question (as written or your suggested modification). | ◯ High priority<br>◯ Moderate priority<br>◯ Low priority |
| Any additional research questions, comments or feedback you would like to add regarding research related to effectiveness of the Levin gastric tube in CEmONC settings? | ____________________________________ |

## RESEARCH at BEmONC SETTINGS
### Levin gastric tube

| | |
|---|---|
| Do you believe that any efficacy or effectiveness studies related to the Levin gastric tube for PPH management should be conducted at BEmONC settings in an LMIC? | ◯ Yes<br>◯ Yes, but only after its efficacy has been evaluated in a CEmONC setting<br>◯ Yes, but only after its efficacy and effectiveness have been evaluated at a CEmONC setting<br>◯ No<br>◯ Not sure |
| In adequately resourced BEmONC settings in an LMIC, what is the efficacy and safety of using the Levin gastric tube compared to standard of care in the reduction of PPH-related maternal morbidity and mortality among women who experience refractory PPH prior to referral?<br><br>This study would be conducted in a highly controlled research LMIC BEmONC setting to test whether the Levin gastric tube (administered by a nurse or midwife) works to improve PPH outcomes, such as stabilize a woman who requires transfer to a higher level of care for PPH. | ◯ Keep<br>◯ Keep with modifications<br>◯ Remove |
| Please enter your suggested modification. | ______________________________ |
| Please rate the priority of this research question (as written or your suggested modification). | ◯ High priority<br>◯ Moderate priority<br>◯ Low priority |
| In an adequately resourced BEmONC setting in an LMIC, what is the efficacy and safety of using the Levin gastric tube compared to standard of care in the reduction of PPH-related maternal morbidity and mortality among women with PPH due to uterine atony who do not respond to the first response bundle?<br><br>This study would be conducted in a highly controlled research LMIC BEmONC setting to test whether the Levin gastric tube (administered by a nurse or midwife) works to improve PPH outcomes, such as control PPH so that referral can be avoided. | ◯ Keep<br>◯ Keep with modifications<br>◯ Remove |
| Please enter your suggested modification. | ______________________________ |
| Please rate the priority of this research question (as written or your suggested modification). | ◯ High priority<br>◯ Moderate priority<br>◯ Low priority |
| Any additional research questions, comments or feedback you would like to add regarding research related to the Levin gastric tube in BEmONC settings? | ______________________________ |

If there are other research questions not included above that you feel are priority areas for the Levin gastric tube in either CEmONC or BEmONC settings, please include them here. __________________________________

## XSTAT SPONGE

When asked what type of priority research should be conducted for the XSTAT sponge, here is a summary of your collective responses.

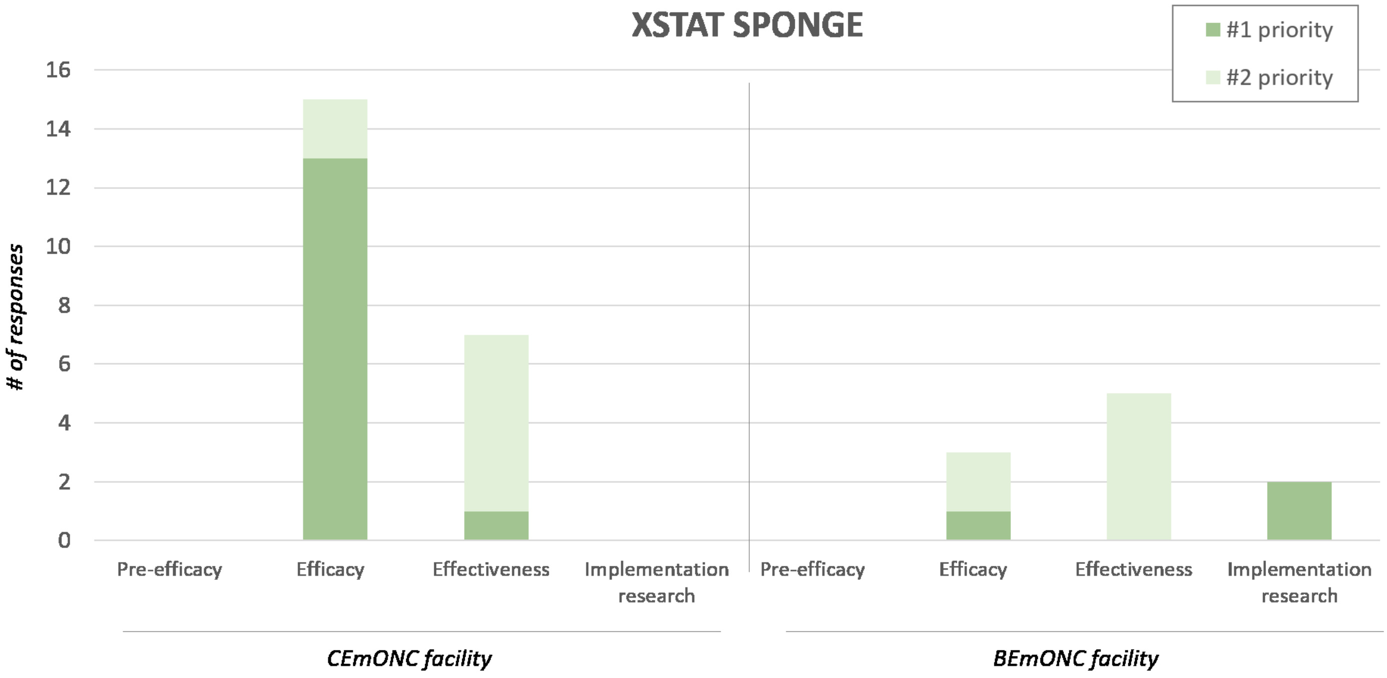


Within the small group that discussed the XSTAT, the following research questions were identified for LMIC settings. For each proposed question, please select whether you would keep, modify or remove it.

Please "keep" questions that you feel are important to address in the next 5 years.

## EFFICACY & SAFETY | CEmONC SETTING
## XSTAT sponge

In an adequately resourced CEmONC setting in an LMIC, what is the efficacy and safety of using XSTAT compared to standard of care in the reduction of PPH-related maternal morbidity and mortality among women with refractory PPH due to uterine atony?

This study would be conducted in a highly controlled research LMIC CEmONC setting to test whether XSTAT works to control refractory PPH due to atony.

- ◯ Keep
- ◯ Keep with modifications
- ◯ Remove

Please enter your suggested modification. __________________________________

| | |
|---|---|
| What types of births/deliveries should this research include? | ◯ All births (vaginal or CS)<br>◯ Vaginal delivery only<br>◯ Vaginal delivery with no prior CS<br>◯ Only CS<br>(Select only one.) |
| Please rate the priority of this research question (as written or your suggested modification). | ◯ High priority<br>◯ Moderate priority<br>◯ Low priority |
| In an adequately resourced CEmONC setting in an LMIC, what is the efficacy and safety of using the XSTAT in the reduction of PPH-related maternal morbidity and mortality among women who experience PPH due to atony who do not respond to the first response bundle?<br><br>This study would be conducted in a highly controlled research LMIC CEmONC setting to test whether XSTAT works to control PPH after the first response bundle, including uterine massage, oxytocic drugs, TXA, IV fluids and examination & escalation. | ◯ Keep<br>◯ Keep with modifications<br>◯ Remove |
| Please enter your suggested modification. | __________________________________ |
| What types of births/deliveries should this research include? | ◯ All births (vaginal or CS)<br>◯ Vaginal delivery only<br>◯ Vaginal delivery with no prior CS<br>◯ Only CS<br>(Select only one.) |
| Please rate the priority of this research question (as written or your suggested modification). | ◯ High priority<br>◯ Moderate priority<br>◯ Low priority |
| Any additional research questions, comments or feedback you would like to add regarding research related to efficacy of the XSTAT sponge in CEmONC settings? | __________________________________ |

## EFFECTIVENESS | CEmONC SETTING

## XSTAT sponge

| | |
|---|---|
| In adequately resourced CEmONC settings in an LMIC, what is the effectiveness of using the XSTAT compared to standard of care in the reduction of PPH-related maternal morbidity and mortality among women who experience refractory PPH due to uterine atony?<br><br>This study would be conducted in a real-world LMIC CEmONC setting to test whether the XSTAT works to control refractory PPH due to atony. | ◯ Keep<br>◯ Keep with modifications<br>◯ Remove |
| Please enter your suggested modification. | __________________________________ |

| | |
|---|---|
| What types of births/deliveries should this research include? | ◯ All births (vaginal or CS)<br>◯ Vaginal delivery only<br>◯ Vaginal delivery with no prior CS<br>◯ Only CS<br>(Select only one.) |
| Please rate the priority of this research question (as written or your suggested modification). | ◯ High priority<br>◯ Moderate priority<br>◯ Low priority |
| In adequately resourced CEmONC settings in an LMIC, what is the effectiveness of using the XSTAT compared to standard of care in the reduction of PPH-related maternal morbidity and mortality among women who experience PPH due to atony who do not respond to the first response bundle?<br><br>This study would be conducted in a real-world LMIC CEmONC setting to test whether the XSTAT works to control PPH after the first response bundle, including uterine massage, oxytocic drugs, TXA, IV fluids and examination & escalation. | ◯ Keep<br>◯ Keep with modifications<br>◯ Remove |
| Please enter your suggested modification. | __________________________________ |
| What types of births/deliveries should this research include? | ◯ All births (vaginal or CS)<br>◯ Vaginal delivery only<br>◯ Vaginal delivery with no prior CS<br>◯ Only CS<br>(Select only one.) |
| Please rate the priority of this research question (as written or your suggested modification). | ◯ High priority<br>◯ Moderate priority<br>◯ Low priority |
| Any additional research questions, comments or feedback you would like to add regarding research related to effectiveness of the XSTAT sponge in CEmONC settings? | __________________________________ |

## RESEARCH at BEmONC SETTINGS

## XSTAT sponge

| | |
|---|---|
| Do you believe that any efficacy or effectiveness studies related to the XSTAT sponge for PPH management should be conducted at BEmONC settings in an LMIC? | ◯ Yes<br>◯ Yes, but only after its efficacy has been evaluated in a CEmONC setting<br>◯ Yes, but only after its efficacy and effectiveness have been evaluated at a CEmONC setting<br>◯ No<br>◯ Not sure |

| | |
|---|---|
| In an adequately resourced BEmONC setting in an LMIC, what is the efficacy and safety of using XSTAT compared to standard of care in the reduction of PPH-related maternal morbidity and mortality among women with refractory PPH prior to referral?<br><br>This study would be conducted in a highly controlled research LMIC BEmONC setting to test whether XSTAT (administered by a nurse or midwife) works to improve PPH outcomes, such as stabilize a woman who requires transfer to a higher level of care for PPH. | ◯ Keep<br>◯ Keep with modifications<br>◯ Remove |
| Please enter your suggested modification. | __________________________________ |
| Please rate the priority of this research question (as written or your suggested modification). | ◯ High priority<br>◯ Moderate priority<br>◯ Low priority |
| In an adequately resourced BEmONC setting in an LMIC, what is the efficacy and safety of using XSTAT compared to standard of care in the reduction of PPH-related maternal morbidity and mortality among women with PPH due to uterine atony who do not respond to the first response bundle?<br><br>This study would be conducted in a highly controlled research LMIC BEmONC setting to test whether XSTAT (administered by a nurse or midwife) works to improve PPH outcomes, such as control PPH so that referral can be avoided. | ◯ Keep<br>◯ Keep with modifications<br>◯ Remove |
| Please enter your suggested modification. | __________________________________ |
| Please rate the priority of this research question (as written or your suggested modification). | ◯ High priority<br>◯ Moderate priority<br>◯ Low priority |
| Any additional research questions, comments or feedback  you would like to add regarding research related to the XSTAT sponge in BEmONC settings? | __________________________________ |
| If there are other research questions not included above that you feel are priority areas for the XSTAT sponge in either CEmONC or BEmONC settings, please include them here. | __________________________________ |

## CELOX GAUZE

When asked what type of priority research should be conducted for the Celox gauze, here is a summary of your collective responses.

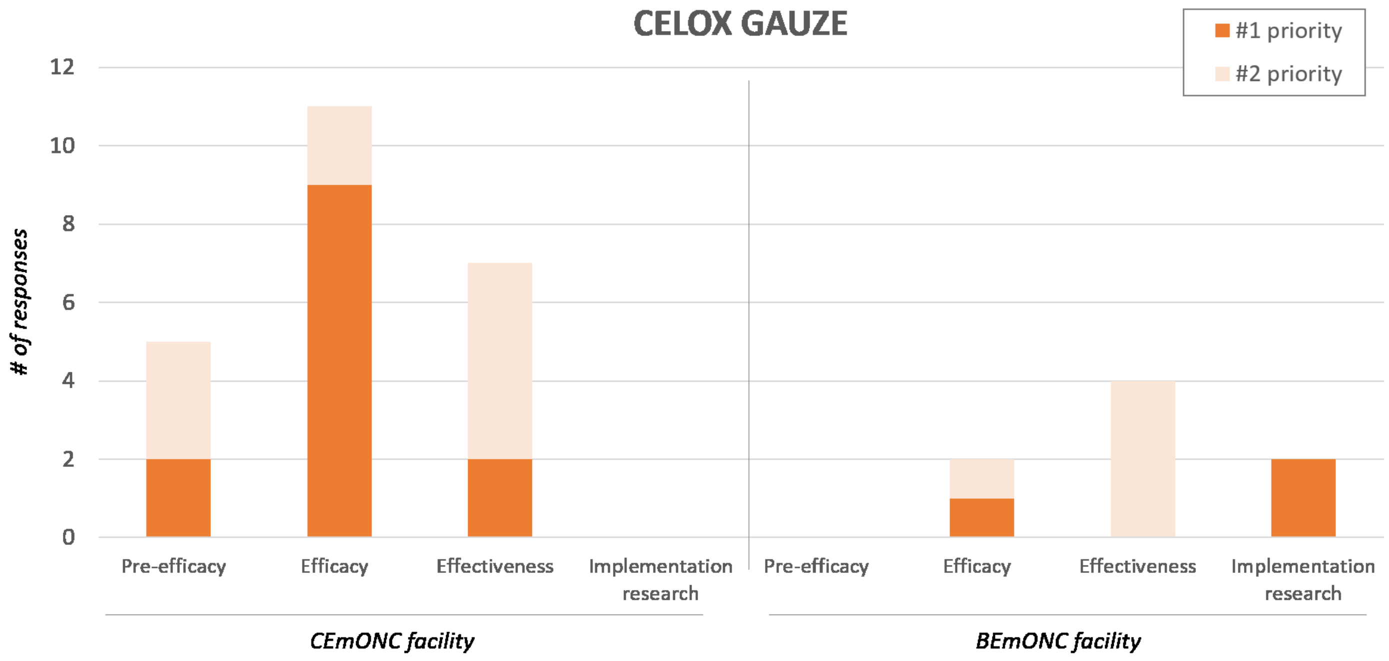


Within the small group that discussed Celox, the following research questions were identified for LMIC settings. For each proposed question, please select whether you would keep, modify or remove it.

Please "keep" questions that you feel are important to address in the next 5 years.

## EFFICACY & SAFETY| CEmONC SETTING
## Celox gauze

In an adequately resourced CEmONC setting with ability to respond to PPH, what is the efficacy and safety of using Celox gauze compared to standard of care in the reduction of PPH-related maternal morbidity and mortality among women who experience refractory PPH due to uterine atony?

This study would be conducted in a highly controlled research LMIC CEmONC setting to test whether Celox gauze works to control refractory PPH due to atony.

◯ Keep
◯ Keep with modifications
◯ Remove

Please enter your suggested modification.

__________________________________

What types of births/deliveries should this research include?

◯ All births (vaginal or CS)
◯ Vaginal delivery only
◯ Vaginal delivery with no prior CS
◯ Only CS
(Select only one.)

Please rate the priority of this research question (as written or your suggested modification).

◯ High priority
◯ Moderate priority
◯ Low priority

| | |
|---|---|
| In adequately resourced CEmONC settings with ability to respond to PPH, what is the efficacy and safety of using Celox gauze compared to standard of care in the reduction of PPH-related maternal morbidity and mortality among women who experience PPH due to atony who do not respond to the first response bundle?<br><br>This study would be conducted in a highly controlled research LMIC CEmONC setting to test whether Celox gauze works to control PPH after the first response bundle, including uterine massage, oxytocic drugs, TXA, IV fluids and examination & escalation. | ◯ Keep<br>◯ Keep with modifications<br>◯ Remove |
| Please enter your suggested modification. | __________________________________ |
| What types of births/deliveries should this research include? | ◯ All births (vaginal or CS)<br>◯ Vaginal delivery only<br>◯ Vaginal delivery with no prior CS<br>◯ Only CS<br>(Select only one.) |
| Please rate the priority of this research question (as written or your suggested modification). | ◯ High priority<br>◯ Moderate priority<br>◯ Low priority |
| Any additional research questions, comments or feedback you would like to add regarding research related to efficacy of Celox gauze in CEmONC settings? | __________________________________ |

**EFFECTIVENESS | CEmONC SETTING**

**Celox gauze**

| | |
|---|---|
| In adequately resourced CEmONC settings in an LMIC, what is the effectiveness of using the Celox gauze compared to standard of care in the reduction of PPH-related maternal morbidity and mortality among women who experience refractory PPH due to uterine atony?<br><br>This study would be conducted in a real-world LMIC CEmONC setting to test whether the Celox gauze works to control refractory PPH due to atony. | ◯ Keep<br>◯ Keep with modifications<br>◯ Remove |
| Please enter your suggested modification. | __________________________________ |
| What types of births/deliveries should this research include? | ◯ All births (vaginal or CS)<br>◯ Vaginal delivery only<br>◯ Vaginal delivery with no prior CS<br>◯ Only CS<br>(Select only one.) |
| Please rate the priority of this research question (as written or your suggested modification). | ◯ High priority<br>◯ Moderate priority<br>◯ Low priority |

| | |
|---|---|
| In adequately resourced CEmONC settings in an LMIC, what is the effectiveness of using the Celox gauze compared to standard of care in the reduction of PPH-related maternal morbidity and mortality among women who experience PPH due to atony who do not respond to the first response bundle?<br><br>This study would be conducted in a real-world LMIC CEmONC setting to test whether the Celox gauze works to control PPH after the first response bundle, including uterine massage, oxytocic drugs, TXA, IV fluids and examination & escalation. | ◯ Keep<br>◯ Keep with modifications<br>◯ Remove |
| Please enter your suggested modification. | __________________________________ |
| What types of births/deliveries should this research include? | ◯ All births (vaginal or CS)<br>◯ Vaginal delivery only<br>◯ Vaginal delivery with no prior CS<br>◯ Only CS<br>(Select only one.) |
| Please rate the priority of this research question (as written or your suggested modification). | ◯ High priority<br>◯ Moderate priority<br>◯ Low priority |
| Any additional research questions, comments or feedback you would like to add regarding research related to effectiveness of Celox gauze in CEmONC settings? | __________________________________ |

**RESEARCH at BEmONC SETTINGS**

**Celox gauze**

| | |
|---|---|
| Do you believe that any efficacy or effectiveness studies related to the Celox gauze sponge for PPH management should be conducted at BEmONC settings in an LMIC? | ◯ Yes<br>◯ Yes, but only after its efficacy has been evaluated in a CEmONC setting<br>◯ Yes, but only after its efficacy and effectiveness have been evaluated at a CEmONC setting<br>◯ No<br>◯ Not sure |
| In an adequately resourced BEmONC setting in an LMIC, what is the efficacy and safety of Celox gauze compared to standard of care in the reduction of PPH-related maternal morbidity and mortality among women with refractory PPH prior to referral?<br><br>This study would be conducted in a highly controlled research LMIC BEmONC setting to test whether Celox gauze (administered by a nurse or midwife) works to improve PPH outcomes, such as stabilize a woman who requires transfer to a higher level of care for PPH. | ◯ Keep<br>◯ Keep with modifications<br>◯ Remove |
| Please enter your suggested modification. | __________________________________ |

| | |
|---|---|
| Please rate the priority of this research question (as written or your suggested modification). | ◯ High priority<br>◯ Moderate priority<br>◯ Low priority |
| In an adequately resourced BEmONC setting in an LMIC, what is the efficacy and safety of using Celox gauze compared to standard of care in the reduction of PPH-related maternal morbidity and mortality among women with PPH due to uterine atony who do not respond to the first response bundle?<br><br>This study would be conducted in a highly controlled research LMIC BEmONC setting to test whether Celox gauze (administered by a nurse or midwife) works to improve PPH outcomes, such as control PPH so that referral can be avoided. | ◯ Keep<br>◯ Keep with modifications<br>◯ Remove |
| Please enter your suggested modification. | __________________________________ |
| Please rate the priority of this research question (as written or your suggested modification). | ◯ High priority<br>◯ Moderate priority<br>◯ Low priority |
| Any additional research questions, comments or feedback you would like to add regarding research related to the Celox gauze in BEmONC settings? | __________________________________ |
| If there are other research questions not included above that you feel are priority areas for the Celox gauze in either CEmONC or BEmONC settings, please include them here. | __________________________________ |

**SECTION C. OTHER CROSS-CUTTING RESEARCH QUESTIONS.**
**Within each of the breakout groups, other research questions were raised that we felt could be applicable across the tools. Using a Likert scale (1=high priority; 2=moderate priority; 3=low priority), please rank how important you feel it is to explore the following questions.**

| | |
|---|---|
| How important is research to assess relative efficacy between two or more of these suction or sponge tools in LMICs (for example, Jada compared to XSTAT)? | ◯ High priority<br>◯ Moderate priority<br>◯ Low priority |

**JADA DEVICE.**

| | High priority | Moderate priority | Low priority |
|---|---|---|---|
| What is the usability and acceptability (e.g., ease and speed of use) of the Jada device from the provider perspective? | ◯ | ◯ | ◯ |

| | | | |
|---|---|---|---|
| In order to inform feasibility at BEmONC setting, can midwives be trained to insert the Jada device so that it can be used in the absence of an OBGYN? | ○ | ○ | ○ |
| In order to inform feasibility at BEmONC setting, can the Jada device be modified for use for settings with no electricity (e.g., battery suction, MVA syringe)? | ○ | ○ | ○ |
| In order to inform acceptability in LMICs where many women do not receive regional anesthesia, what is the patient experience (e.g., pain, discomfort) if the Jada device is used? | ○ | ○ | ○ |
| What are the risks of adverse events, such as perforation, infection, inadequate placement (placement in the vagina, instead of uterus) for the Jada device? | ○ | ○ | ○ |
| What are the costs associated with use of the Jada device for PPH management compared to standard of care? | ○ | ○ | ○ |

## LEVIN GASTRIC TUBE.

| | High priority | Moderate priority | Low priority |
|---|---|---|---|
| What is the usability and acceptability (e.g., ease and speed of use) of the Levin gastric tube from the provider perspective? | ○ | ○ | ○ |
| In order to inform feasibility at BEmONC setting, can midwives be trained to insert the Levin gastric tube so that it can be used in the absence of an OBGYN? | ○ | ○ | ○ |

| | | | |
|---|---|---|---|
| In order to inform feasibility at BEmONC setting, can the Levin gastric tube be modified for use for settings with no electricity (e.g., battery suction, MVA syringe)? | ○ | ○ | ○ |
| In order to inform acceptability in LMICs where many women do not receive regional anesthesia, what is the patient experience (e.g., pain, discomfort) if the Levin gastric tube is used? | ○ | ○ | ○ |
| What are the risks of adverse events, such as perforation, infection, inadequate placement (placement in the vagina, instead of uterus) for the Levin gastric tube? | ○ | ○ | ○ |
| What are the costs associated with use of the Levin gastric tube for PPH management compared to standard of care? | ○ | ○ | ○ |

**XSTAT SPONGE.**

| | High priority | Moderate priority | Low priority |
|---|---|---|---|
| What is the usability and acceptability (e.g., ease and speed of use) of the XSTAT sponge from the provider perspective? | ○ | ○ | ○ |
| In order to inform feasibility at BEmONC setting, can midwives be trained to insert the XSTAT sponge so that it can be used in the absence of an OBGYN? | ○ | ○ | ○ |
| In order to inform feasibility at BEmONC setting, can the XSTAT sponge be successfully placed in the intrauterine cavity without ultrasound guidance? | ○ | ○ | ○ |

| | | | |
|---|---|---|---|
| In order to inform acceptability in LMICs where many women do not receive regional anesthesia, what is the patient experience (e.g., pain, discomfort) if the XSTAT sponge is used? | ○ | ○ | ○ |
| What are the risks of adverse events, such as infection, inadequate placement (placement in the vagina, instead of uterus) for the XSTAT sponge? | ○ | ○ | ○ |
| What are the costs associated with use of the XSTAT sponge for PPH management compared to standard of care? | ○ | ○ | ○ |
| Can the XSTAT sponge be used for any cause of PPH (e.g., placenta bed abnormality, vaginal/cervical tears)? | ○ | ○ | ○ |

## CELOX GAUZE.

| | High priority | Moderate priority | Low priority |
|---|---|---|---|
| What is the usability and acceptability (e.g., ease and speed of use) of the Celox gauze from the provider perspective? | ○ | ○ | ○ |
| In order to inform feasibility at BEmONC setting, can midwives be trained to insert the Celox gauze so that it can be used in the absence of an OBGYN? | ○ | ○ | ○ |
| In order to inform feasibility at BEmONC setting, can the Celox gauze be successfully placed in the intrauterine cavity without ultrasound guidance? | ○ | ○ | ○ |
| In order to inform acceptability in LMICs where many women do not receive regional anesthesia, what is the patient experience (e.g., pain, discomfort) if the Celox gauze is used? | ○ | ○ | ○ |

| | | | |
|---|---|---|---|
| What are the risks of adverse events, such as infection, inadequate placement (placement in the vagina, instead of uterus) for the Celox gauze? | ○ | ○ | ○ |
| What are the costs associated with use of the Celox gauze for PPH management compared to standard of care? | ○ | ○ | ○ |
| Can the Celox gauze be used for any cause of PPH (e.g., placenta bed abnormality, vaginal/cervical tears)? | ○ | ○ | ○ |

**SECTION D. CLOSING.**

**We appreciate your participation in this survey. We hope this work can inform collaborative and coordinated efforts to move the field forward.**

If you have any other comments or considerations you would like to share that were not covered in the sections above, please feel free to use the space below. __________________________________

If you are interested in continued collaboration as we develop the manuscript related to this process, please provide your name. ______________________________

Please provide your email address. ______________________________

End of survey.

THANK YOU!
