# Supplementary material for: Global research priorities for intrauterine suction and sponge tools for postpartum haemorrhage management in low-income and middle-income countries: a modified Delphi approach
Source: BMJ Public Health. 2024 May 30;2(1):e000113. doi: 10.1136/bmjph-2023-000113 (PMC11812741; doi:10.1136/bmjph-2023-000113)
Supplement: online supplemental file 4 [file bmjph-2-1-s004.pdf]

| DEVICE | STUDIES | STUDY N | SUCTION | METHOD NOTES | INSERTION TIME | OUTCOMES | LOCATION |
|---|---|---|---|---|---|---|---|
| FG36 Levin stomach tube, later termed Suction Tube Uterine Tamponade<br><br>Wide-bore (FG24 to FG36) Levin suction tube connected by suction tubing to an adjustable electronic suction pump or wall suction source | Case series, Hofmeyr 2020 | 3 women | 100-200mmHg | - Held about 20 cm from the tip, concave anterior, and introduced along the anterior cervical lip into the uterine cavity<br>- Tube becomes fixed in uterus<br>- Taped to the woman's thigh with some slack | in place for 1 hour; up to 4 hours | - Insertion takes less than 1 minute.<br>- Suction found to be effective | Frere Maternity Hospital, South Africa |
| | Randomized single-center, double-blind feasibility study Hofmeyr, 2019 | 45 women all CS | 120mmHG | Tube placed during cs connected transvaginally to suction; randomized to early or delayed UST (after uterus closure vs. after closure of skin wound) | 14 min early UST; 4 min delayed UST | - No difference in the measured blood loss or in any of the secondary outcomes between the two study groups.<br>- No cases of postoperative complications, blood transfusion, wound infection, or endometritis. | Frere Maternity Hospital, South Africa |
| 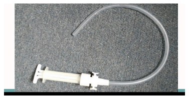 | Internal pilot of RCT Cebekuhu 2021 (Hofmeyr senior author) | 12 women STUT vs. 12 women UBT | Not stated | - Feasibility and acceptability<br>- Examined women's perspectives<br>- All received additional uterotonics (oxytocin, ergo/syn, miso, TXA) | If effective, suction interrupted every 30–60 min and removed as soon as the bleeding was controlled without suction | - Insertion difficult: 3/10 STUT and 4/9 UBT<br>- two laparotomies and one intensive care unit admission in the UBT group.<br>- Pain during STUT insertion: none/mild in 9/10 and severe in 1/10<br>- STUT experience: fine 4/11 and "uncomfortable but acceptable" in 7/11 | 10 secondary and tertiary hospitals, South Africa |

**Updated May 2022. Please note that this synthesis may not be comprehensive and is a working document.*

| DEVICE | STUDIES | STUDY N | SUCTION | METHOD NOTES | INSERTION TIME | OUTCOMES | LOCATION |
|---|---|---|---|---|---|---|---|
| JADA device (previously known as InPress)<br><br>Intrauterine tubing loop w holes, inflatable cervical seal<br><br>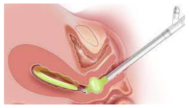 | First-in-human proof of concept study, Purwosunu et al 2016 | 10 women, vaginal delivery | 70mmHg | EBL at time of placement: 600-1000ml<br>Women received PPH management uterotonics per SoC (oxytocin, miso, methergine) | Remained in place for a minimum of 1 hour and up to 6.5 hours in one case. | - immediate seal at the cervical os<br>- 50–250 mL of residual blood was evacuated<br>- Uterus collapsed and regained tone within minutes, and hemorrhaging was controlled | Tertiary hospital, Jakarta Indonesia |
| | Multi-center prospective single-arm treatment study, D'Alton 2020 | 107 women w PPH 500-1500in VD (85%) 1000-1500 in CS (15%) | 70-90 mm Hg | Manual sweep<br>Placement after estimated EBL 500-1500ml VD or 1000-1500CS, after unresponsive to uterotonics and massage | suction on until bleeding controlled plus 1 hour, leave device in 30 min after suction off<br>Median 144 min tx time | - 3 min to bleeding control<br>- 94% successful control<br>- Average 191 minutes indwelling<br>- High provider acceptability | 12 centers in the US (PEARLE study) |
| | Unpublished – found in Jada Instructions for Use | 13 women | Not known | IFU notes that 3 enrollees were beyond inclusion criteria EBL | Not known | 2 deaths following control of abnormal bleeding due to lack of blood product | Nsambya Hospital, Kampala, Uganda |

**Updated May 2022. Please note that this synthesis may not be comprehensive and is a working document.*

| DEVICE | STUDIES | STUDY N | SUCTION | METHOD NOTES | INSERTION TIME | OUTCOMES | LOCATION |
|---|---|---|---|---|---|---|---|
| Vacuum-induced tamponade using a modified balloon system 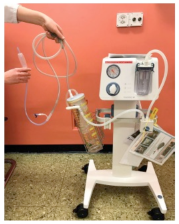 | Single center cohort Haslinger 2021 | 66 women - 2/3 atony, 1/3 placenta abnormality | 60-70kPa | - Compares two observation periods<br>- Bakri balloon was inflated with only 50–100 mL of physiologic saline solution; catheter connected with a nonsterile tube to a vacuum device, and intrauterine vacuum was applied | Median duration 2.5 hrs (1-4hr range) | - Success rate was 82% over study period (improved to 94% in latter half of study)<br>- Success higher in atony vs. placental pathology<br>- EBL 1500ml | University Hospital Zurich |

**Updated May 2022. Please note that this synthesis may not be comprehensive and is a working document.*

| DEVICE | STUDIES | STUDY N | SUCTION | METHOD NOTES | INSERTION TIME | OUTCOMES | LOCATION |
|---|---|---|---|---|---|---|---|
| Specially made stainless steel or plastic cannula 12mm diameter, 25 cm length with multiple 4mm holes with thick-walled suction tube 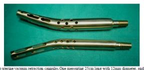 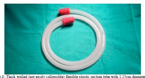 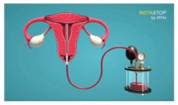 | Prospective observational cohort, Panicker 2014 | 20 women; 16 vaginal, 4 CS with atonic PPH | 650mmHg | -SoC uterotonics received (methergine, oxytocin, carboprost) | Not stated | - Cessation of bleeding was associated with contraction and firm retraction of uterus<br>- Observed in all women within 4mns after initiation of procedure.<br>- The amount of blood collected in suction bottle ranged from 150ml to250ml | Maternity Hospital, India |
| | Prospective observational cohort, Panicker 2017 | 55 women; 40 vaginal delivery, 15 CS with atonic PPH | 700mm Hg | -SoC uterotonics received | Suction maintained for 30 min; 2 cases blocked cannula requiring replacement | Quantity of blood sucked varied from 50–300 ml, after which bleeding ceased. | Maternity Hospital, India |
| | Prospective observational cohort, Meena 2018 | 25 women; 20 vaginal, 5 CS with atonic PPH | 650 mmHg | -SoC uterotonics received – GA 30-40+wks | Removed after 6 hours; temporary adhesions developed due to sucking of soft cervical tissues into perforations required manual manipulation | - 88% cases stopped<br>- bleeding within 4 minutes<br>- 96% blood collected <300ml<br>- 2 deaths (cause unknown) | Pannadhay Zanana Hospital Udaipur, India |

**Updated May 2022. Please note that this synthesis may not be comprehensive and is a working document.*

| DEVICE | STUDIES | STUDY N | SUCTION | METHOD NOTES | INSERTION TIME | OUTCOMES | LOCATION |
|---|---|---|---|---|---|---|---|
| Modified XStat™ Mini Sponge Dressing (MSD) 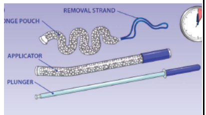  | Feasibility, Rodriguez 2020 Obstetrx,Inc | 9 women, vaginal delivery | Not applicable | - first publication was prototype development & animal testing (sheep)<br>-EBL >500ml | left in place on average for 1 hour (0.5 hours–14 hours). | - Resolution of bleeding within 1 minute. The mean time to place the device was 62 seconds.<br>- Uterine fill was documented in all cases by ultrasound scan<br>- Device placement was rated as "easy" to "very easy."<br>- No bleeding recurrence, no adverse events | University Teaching Hospital Lusaka Zambia |

**Updated May 2022. Please note that this synthesis may not be comprehensive and is a working document.*

| DEVICE | STUDIES | STUDY N | SUCTION | METHOD NOTES | INSERTION TIME | OUTCOMES | LOCATION |
|---|---|---|---|---|---|---|---|
| Celox™ chitosan-covered gauze tamponade 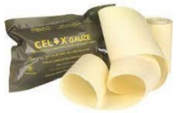  | Schmid 2013 | 19 cases PPH due to atony (13), placenta abnormality (3); other (3); 8 vag and 11 CS | Not applicable | -uterine packing with chitosan-covered gauze<br>- 3m long gauze, removed by end left in vagina<br>- concurrent sulprostone and antibiotics | Up to 24 hours; >30 for 2 cases | - 18/19 bleeding stopped; no further interventions needed<br>- Pre-post showed 75% reduction in hysterectomies<br>- 2 patients persistent spotting, residual chitosan gauze | Marienkrankenhaus Hamburg |
| | Carles 2017 | Case series, 4 cases | Not applicable | 4 types of obstetric hemorrhage: hemostatic hysterectomy, vag tears, atonic PPH, hem CS | Not stated | - gauze and powder used | French Guiana |
| | Dueckelmann 2019<br><br>Additional webinar from Medtrade | 78; 47 Celox; 31 Bakri; mostly atonic | Not applicable | -retrospective 2016-2018<br>- 8 Celox, 2 Bakri injury of cervix or birth canal<br>- cost Celox 50USD vs. Bakri 250USD | Up to 24 hours | - No statistically significant differences between groups (primary outcome: bleeding termination without additional surgical interventions)<br>- 3 in UBT group required hysterectomy, none in gauze group | Charité University Hospital in Berlin, Germany |
| | Biele 2022 | 666 women; 530 medical therapy, 51 balloon, 85 chitosan tamponade (73% atony) | Not applicable | - database retrospective case-control study 2016- 2019<br>- ultrasound guided insertion after Step I/II of PPH algorithm | After 12-24 hours | - no significant differences in the need for surgical therapy<br>- lower number of hysterectomies in the chitosan group than balloon group<br>- 11 cases required both tamponades | Charité University Hospital in Berlin, Germany |

**Updated May 2022. Please note that this synthesis may not be comprehensive and is a working document.*
