# Supplementary material for: Global research priorities for intrauterine suction and sponge tools for postpartum haemorrhage management in low-income and middle-income countries: a modified Delphi approach
Source: BMJ Public Health. 2024 May 30;2(1):e000113. doi: 10.1136/bmjph-2023-000113 (PMC11812741; doi:10.1136/bmjph-2023-000113)
Supplement: online supplemental file 5 [file bmjph-2-1-s005.pdf]

Research Priorities for Sponge and Suction Tools for PPH

KII summary findings

July 2022

Interviews with diverse stakeholders

19 informants interviewed via Zoom

- 4/19 from LMICs
- 10/15 from HICs with LMIC experience

Two interview guides

- Device developers/champions: prior research, next steps, perceived advantages (n=6)
- Clinicians, funders, implementors and policy-makers: prior knowledge, perceptions, research priorities (n=13)*

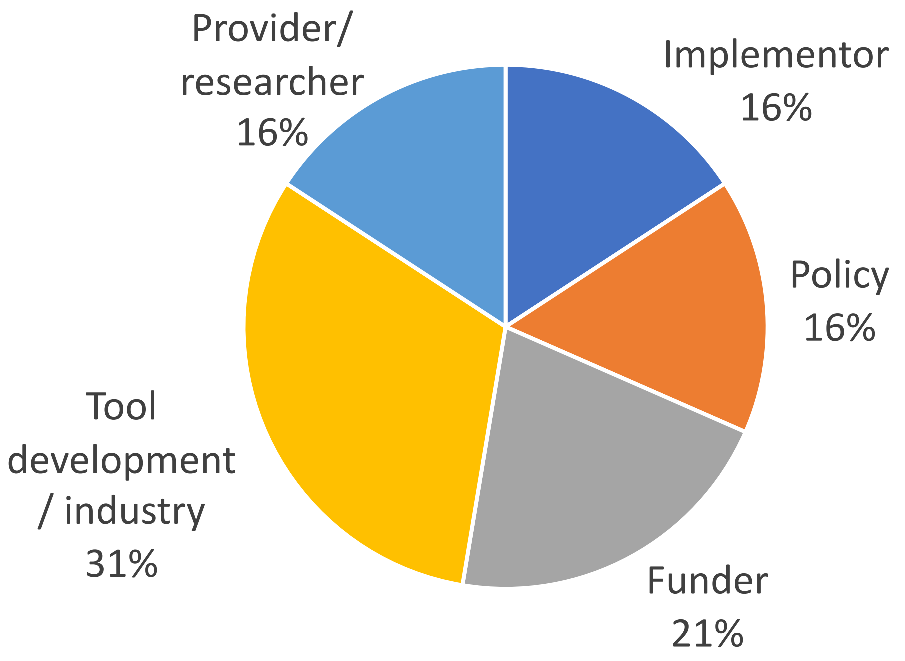

*subsequent KII data among this sub-sample

Opinion split on purpose-built "bespoke" devices vs. improvised tools

I don't believe it needs to a bespoke device. If the system has something that can be deployed - **if we can do something that doesn't require a separate justification specifically for maternal health, [this] might be an appealing option and easier to keep in stock.**

– R10, funder

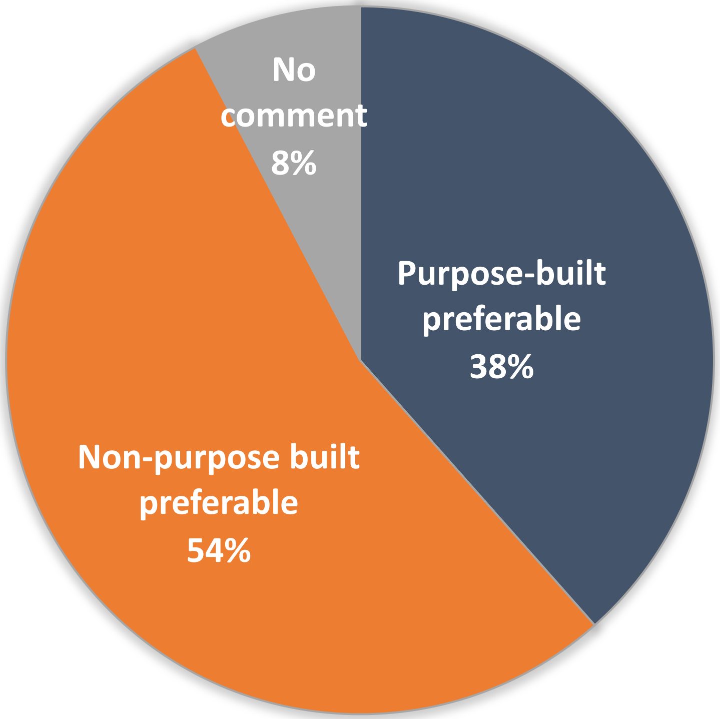

I think there is a market for a device, but not 6 devices. There are strong regulatory authorities, and they should be made available at an accessible price. **The tinkering approach is not acceptable for this class of device.**

- R2, funder

PPH tools in context across the research continuum

Efficacy

Controlled, ideal conditions

*But let's avoid the UBT problem: **First, does it work or not, then the when and where.***

– R4, implementor

Effectiveness

Normal, usual circumstances

***Effectiveness should start at places where women are protected, then move it down to lower levels** for further implementation research or effectiveness work.*

– R6, policy

Implementation

Scale-up

***Start with the end in mind** – what would it take to get a WHO recommendation. Got to design research agenda with that in mind.*

– R10, funder

While most agreed that efficacy has not been demonstrated, many emphasized the need to keep effectiveness and scale-up in mind.

Respondents stressed need to consider contextual factors

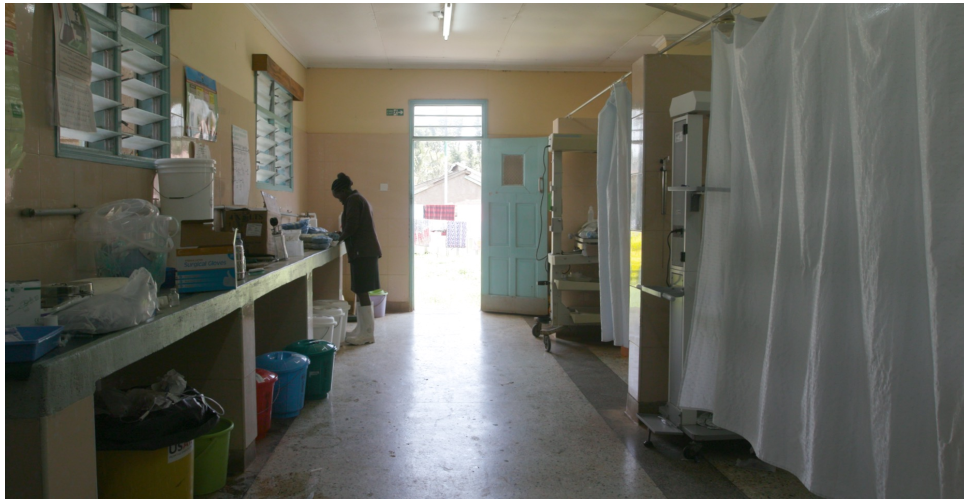

The UBT in my hands is seen as effective, …….But if you take this device to places where PPH occurs at practice level - **lower cadre people with less than optimal skills or even health center IV where the majority of deliveries are occurring, you may have different experiences.**

\- R12, clinician, tertiary hospital

Research parameters

- Study design
- Primary intervention
- Comparator
- Setting
- Sample size

Facility

- First response bundle
- Quality uterotonics
- Ability to refer
- Electricity
- Skills retention

System Factors

- Cost
- Supply chain
- Policy and governance
- Community education/ outreach
