# Supplementary material for: Global research priorities for intrauterine suction and sponge tools for postpartum haemorrhage management in low-income and middle-income countries: a modified Delphi approach
Source: BMJ Public Health. 2024 May 30;2(1):e000113. doi: 10.1136/bmjph-2023-000113 (PMC11812741; doi:10.1136/bmjph-2023-000113)
Supplement: online supplemental file 6 [file bmjph-2-1-s006.pdf]

Emerging tools for PPH management:

Research Prioritization Survey

Summary of results (draft)

July 2022

TABLE OF CONTENTS

Description	Pages
Summary	3
Methods	4
Detailed results	
Demographics	5 - 10
Perceptions & prior knowledge	11 - 16
Research prioritization	17 – 23
Designing an efficacy study	24 - 35
Limitations	36

Summary

Objective:

We surveyed respondents through targeted outreach and snowball sampling to identify priority research questions related to emerging suction/sponge tools for postpartum hemorrhage (PPH) management. The survey was designed to elicit diverse input on efficacy, effectiveness and implementation research. While knowledge, perceptions and general research priority questions were not specific to individual tools, we included some questions whereby respondents were asked to specify priority research elements, such as name of tool to test, type of facility, etc.

Participants:

Respondents included 66 individuals. The majority (74%) either lives or works in a low- or middle-income country (LMIC). Three-quarters of respondents identified as a healthcare provider, the majority of which were OBGYNs who worked at tertiary, private or academic hospitals. Approximately 77% (n=51) had heard about either suction or sponge tools prior to completing this survey of which 16 individuals had used one clinically and/or in a research context.

Findings:

- Majority of respondents perceived these tools to hold promise. The top three challenges they could help overcome included: the inability to manage PPH alone or with limited personnel; inability to stabilize a patient prior to referral; and difficulty with available tools such as UBT.
- Priority research areas identified by respondents:
  - Effectiveness research to understand clinical impact in real-world settings
  - Provider ease of use
  - Level of pain with placement (women's perspective)
  - Cost-effectiveness
- When asked about designing an efficacy trial (i.e., research in an ideal, highly-controlled setting), there was little consensus across different research elements, including setting/facility type, intervention vs. comparator, and outcomes.
- There were some differences when comparing responses from the overall sample vs. those with LMIC experience. For example, LMIC respondents were more likely to indicate use early in the PPH response algorithm (e.g. prior to second-line uterotonics), and that non-physicians should be trained to use these tools (e.g., midwife, nurse)

Collectively, these results underscore the desire for research to span both higher and lower level facilities.

Methods

Context:

The Research Prioritization Survey was deployed as part of an overarching project to understand and arrive at a research prioritization for intrauterine, non-balloon suction and sponge devices for the management of PPH. The project included a literature review, in-depth interviews with key informants, and surveys to understand product characteristics and research priorities. This work will culminate in an expert convening which will be documented with a white paper, research roadmap, Target Product Profile, and/or other summary product.

Survey Development and Deployment:

Research Prioritization survey questions were developed based on a literature review of existing tools and insights from key informant interviews. This survey aimed to elicit input on efficacy, effectiveness and implementation research. Some questions regarding knowledge, perceptions and general research priority questions were not specific to individual suction or sponge tools. However, other questions gathered input on specific research elements, such as name of priority tool to test, type of facility, etc.

Surveys were piloted by three individuals to refine clarity and flow (1 UCSF-based MNCH researcher and 2 LMIC OBGYNs) prior to dissemination. They were then emailed out to professional networks of colleagues working in safe motherhood, PPH, and related topics. Key researchers from publications related to new tools were also included, and all respondents were encouraged to share survey links with their own professional networks. Surveys were accessed via a link and completed in REDCap on a UCSF server. The survey link was left open for over one month (May 11 to June 16, 2022), with weekly reminders sent to email recipients.

Analysis:

Survey data were analyzed descriptively using SPSS and charts/graphs were created using Microsoft Excel. Data were analyzed by those who lived or had experience in LMICs in order to determine if perspectives differed among these groups.

Questions where respondents were asked to rank topics/prompts were averaged, such that lower mean scores represented higher ranking of importance (e.g., 1=highest priority, 5=lowest priority).

Some questions were not answered by all respondents. Sample size (n) reflects those who answered the question, unless noted as “did not state.”

Notes:

This work was led by Dr. Dilys Walker at University of California, San Francisco with funding from the Bill & Melinda Gates Foundation.

Demographics

Detailed results

Residence and location of work

Place of residence/employment (all respondents, n=65)

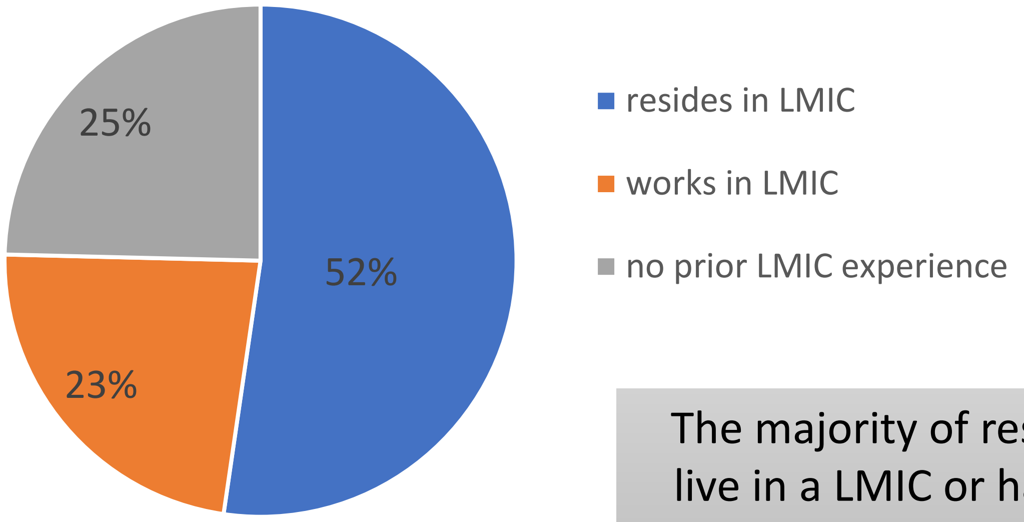

The majority of respondents either live in a LMIC or have employment experience in LMICs.

Non-HIC residents (n=34) were from over a dozen different countries

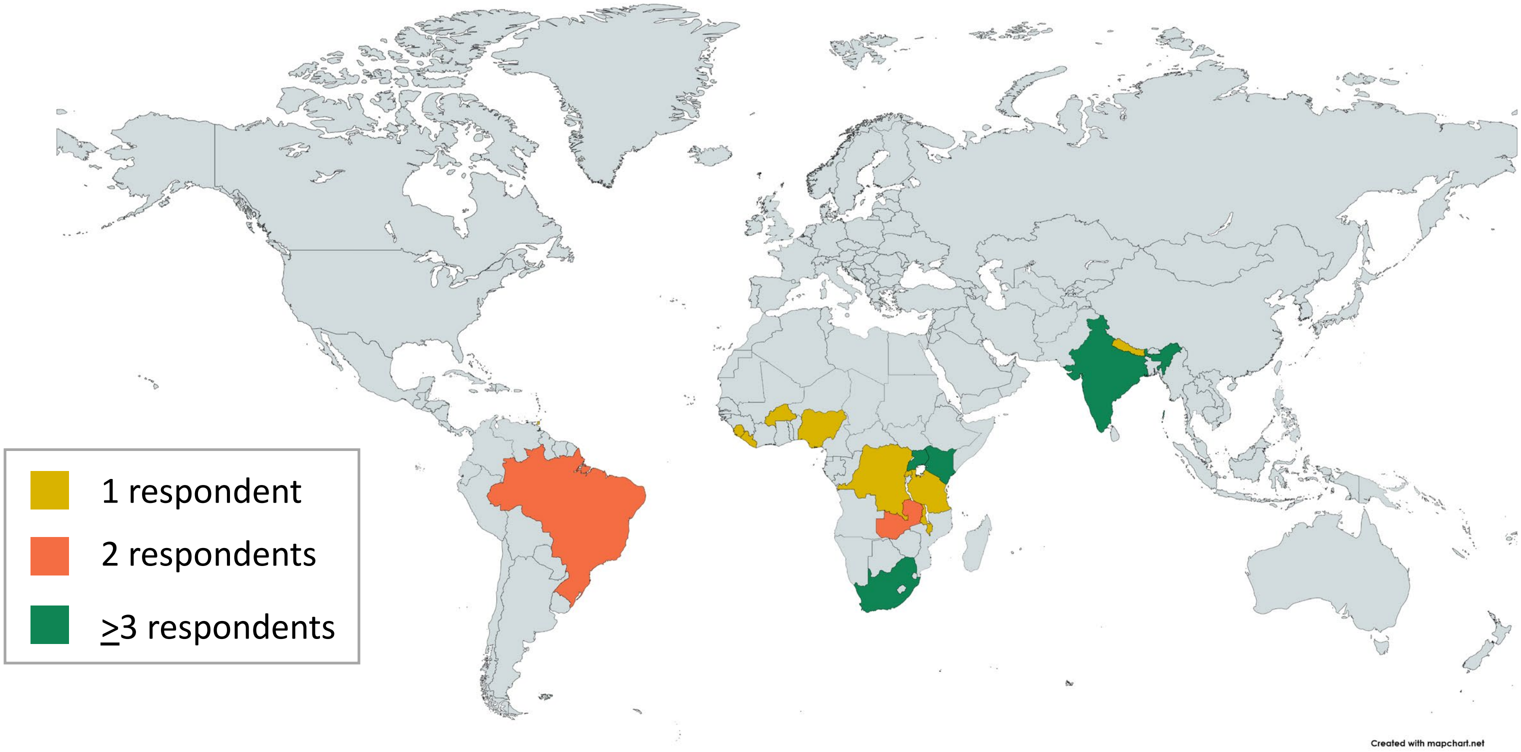

Those with LMIC-related employment experience (n=15) have worked in:

- Asia: Bangladesh, Cambodia, India, Laos, Nepal, Pakistan, Vietnam
- Africa: DRC, Egypt, Ghana, Malawi, Mozambique, Nigeria, Senegal, Tanzania, Uganda
- Other: Afghanistan, Ecuador, Haiti, Papua New Guinea

Profession

Professional background (all respondents, n=66)

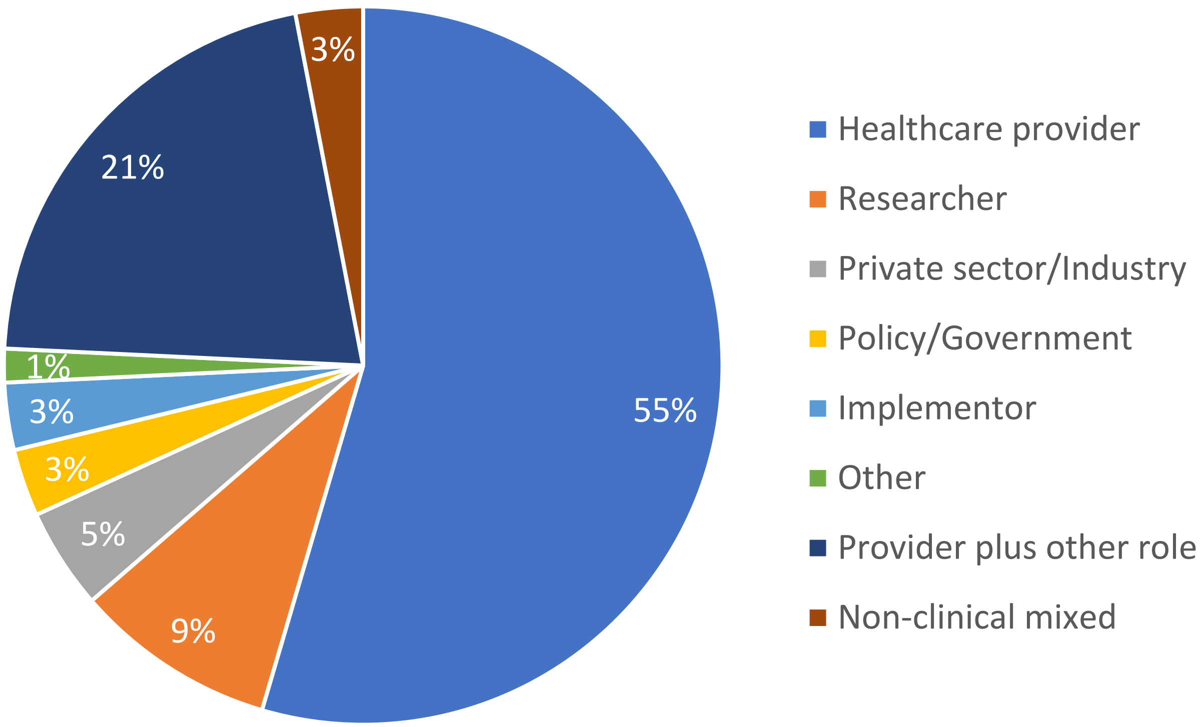

Professional background (respondents with LMIC experience* (n=49)

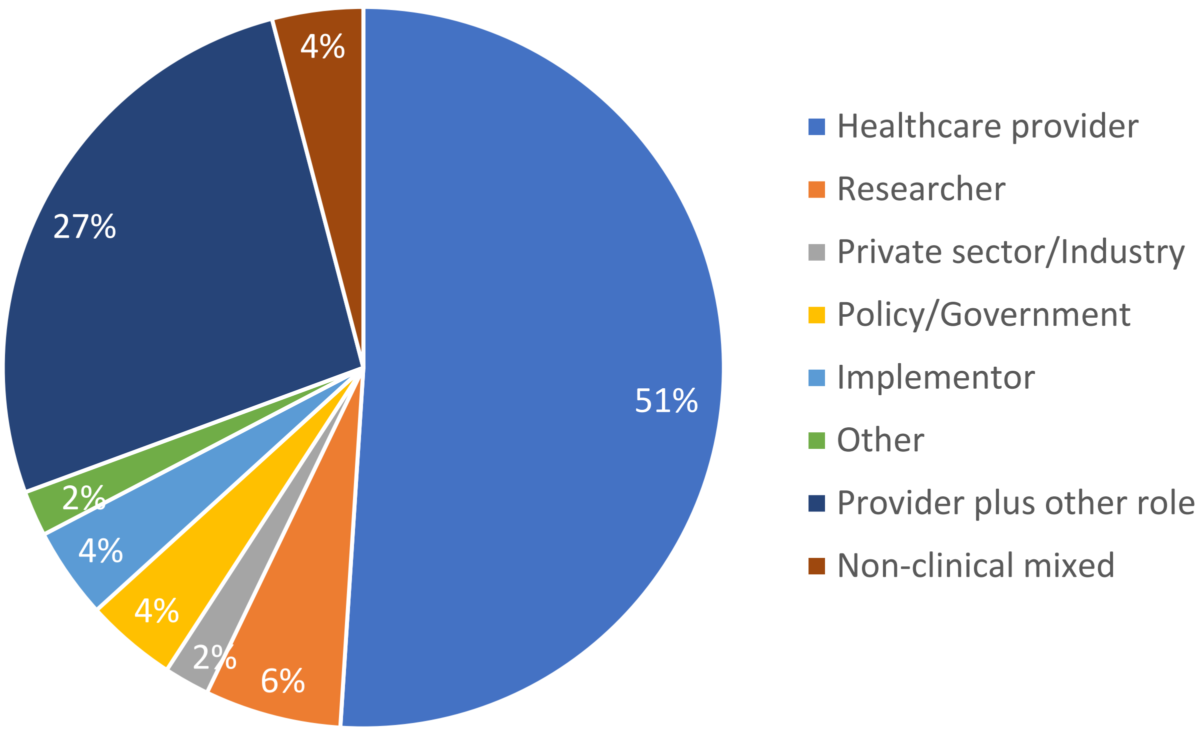

*Either lives or work in LMIC setting

Years of experience in a field related to PPH

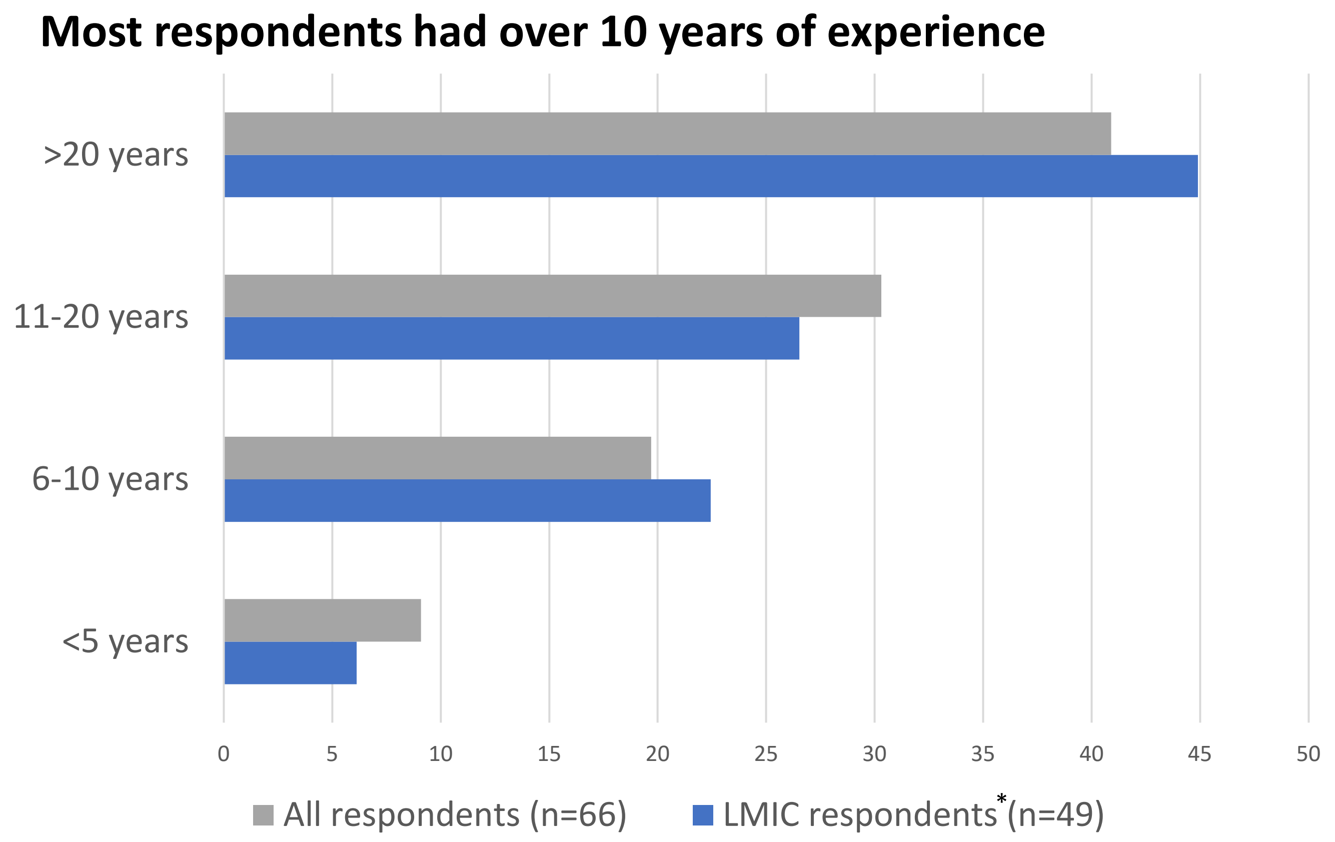

*Either lives or work in LMIC setting

Provider characteristics

Among all respondents, 50 (75.8%) identified as a health care provider.

- 82% of providers (n=41) identified as a clinician educator.
- 64% (n-32) reported being involved in >100 annual deliveries.
- 24% (n=12) identified as a researcher.

Over half of providers live in a LMIC

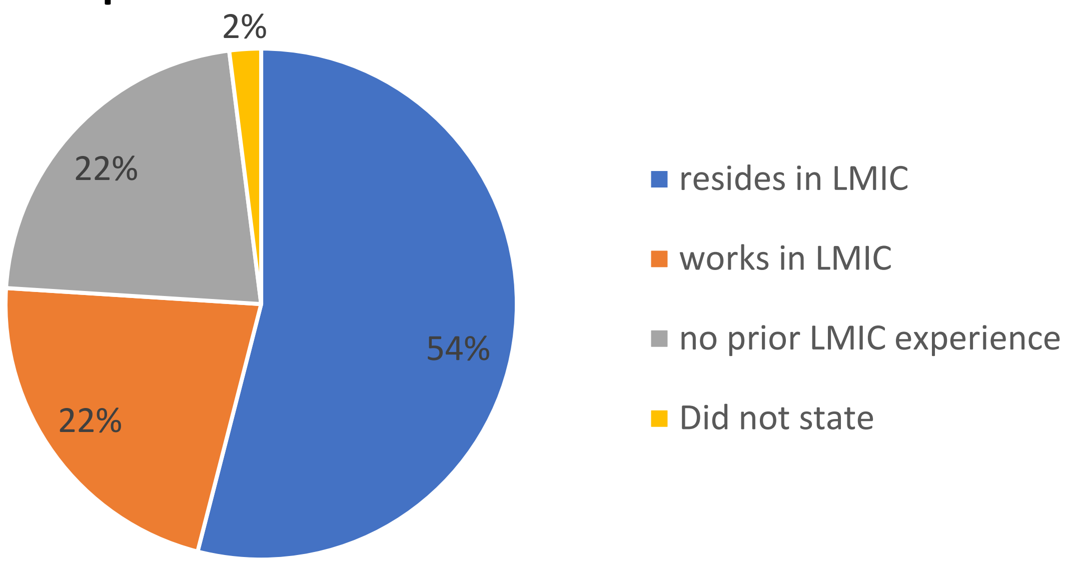

Majority of providers are OBGYNs

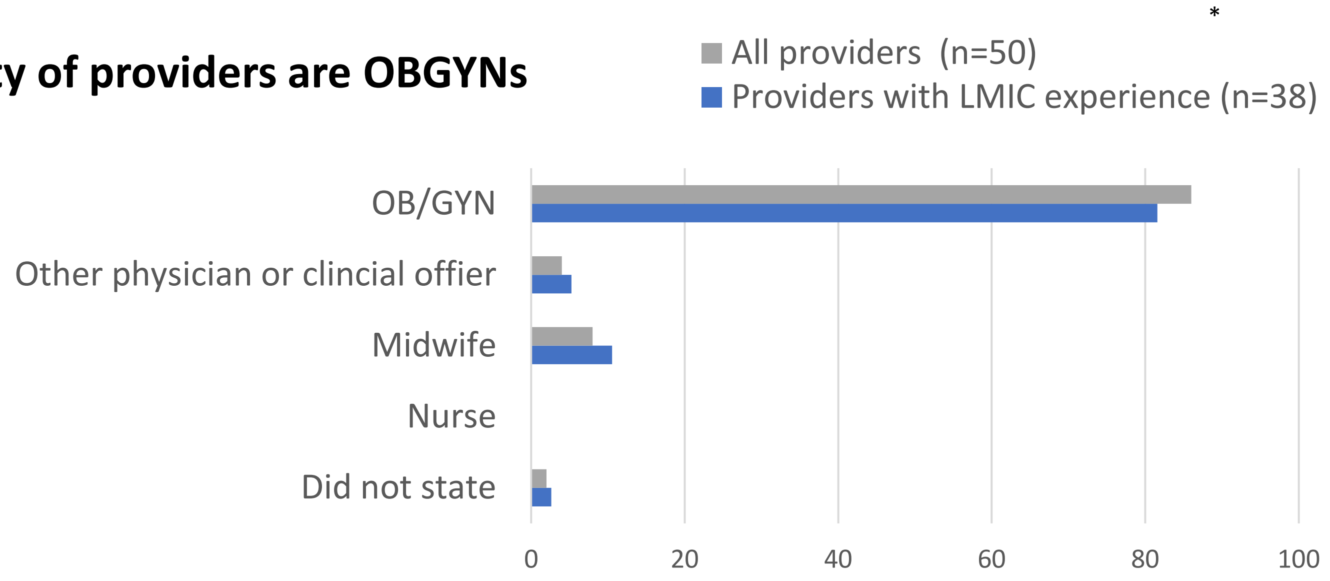

*Either lives or work in LMIC setting

Provider characteristics

Types of facilities providers primarily work

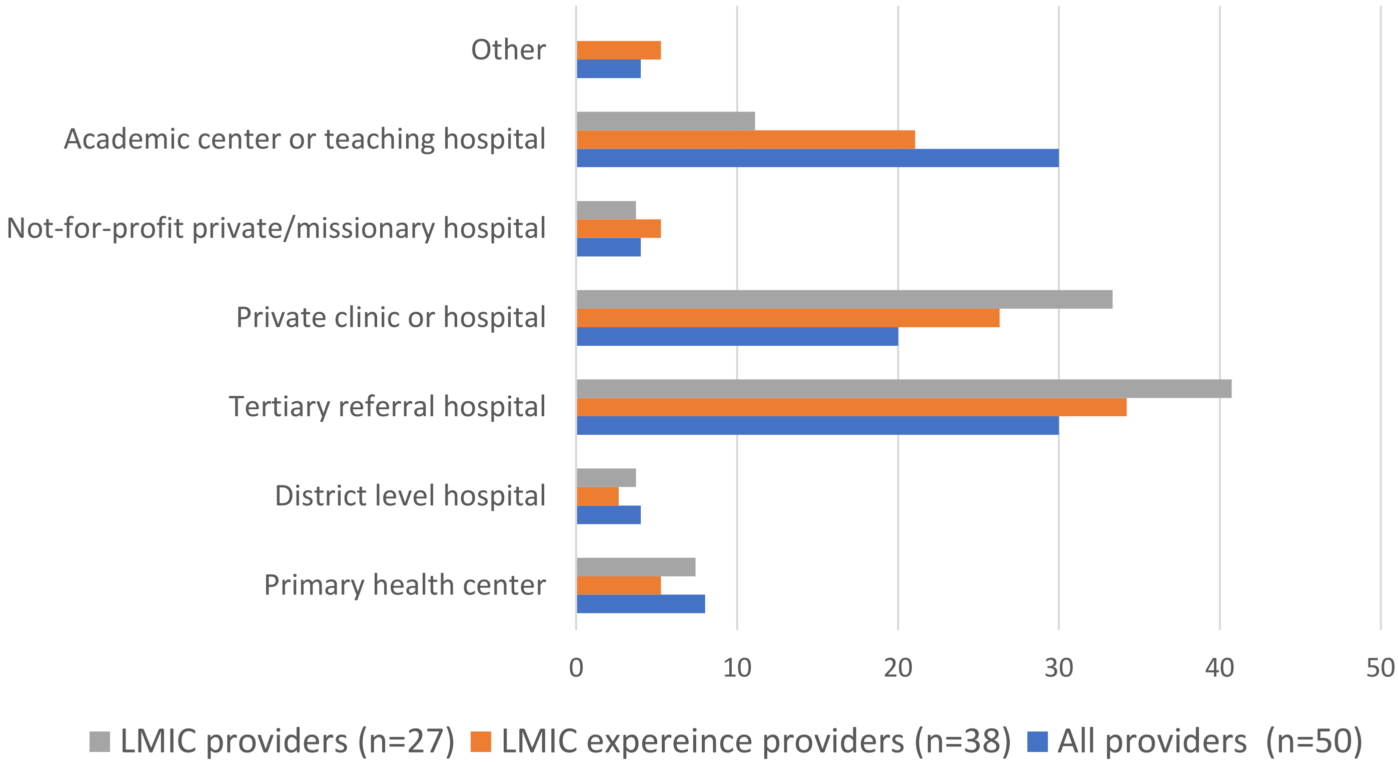

The findings elicited through this survey may be skewed toward clinician/end-user perspectives given that providers comprise the majority of the sample. These individuals are more likely to be OBGYNs who work at higher level facilities.

Knowledge & Perceptions

Detailed findings

Prior knowledge about tools

Majority of respondents had heard about one or more of these tools.

- 51 (77.3%) had heard about either suction or sponge tools
- 16 individuals have used one or more of these types of tools clinically and/or in a clinical research context
    - Jada (1), XSTAT (3), Celox (9), Other tool* (11)
    - **It is possible that some respondents included UBT, Ellavi, foley catheters, and condom tamponade as "other tool." However, it should be noted that the intent was to exclude all UBT tools.*

Prior knowledge about emerging tools (all respondents, n=65)

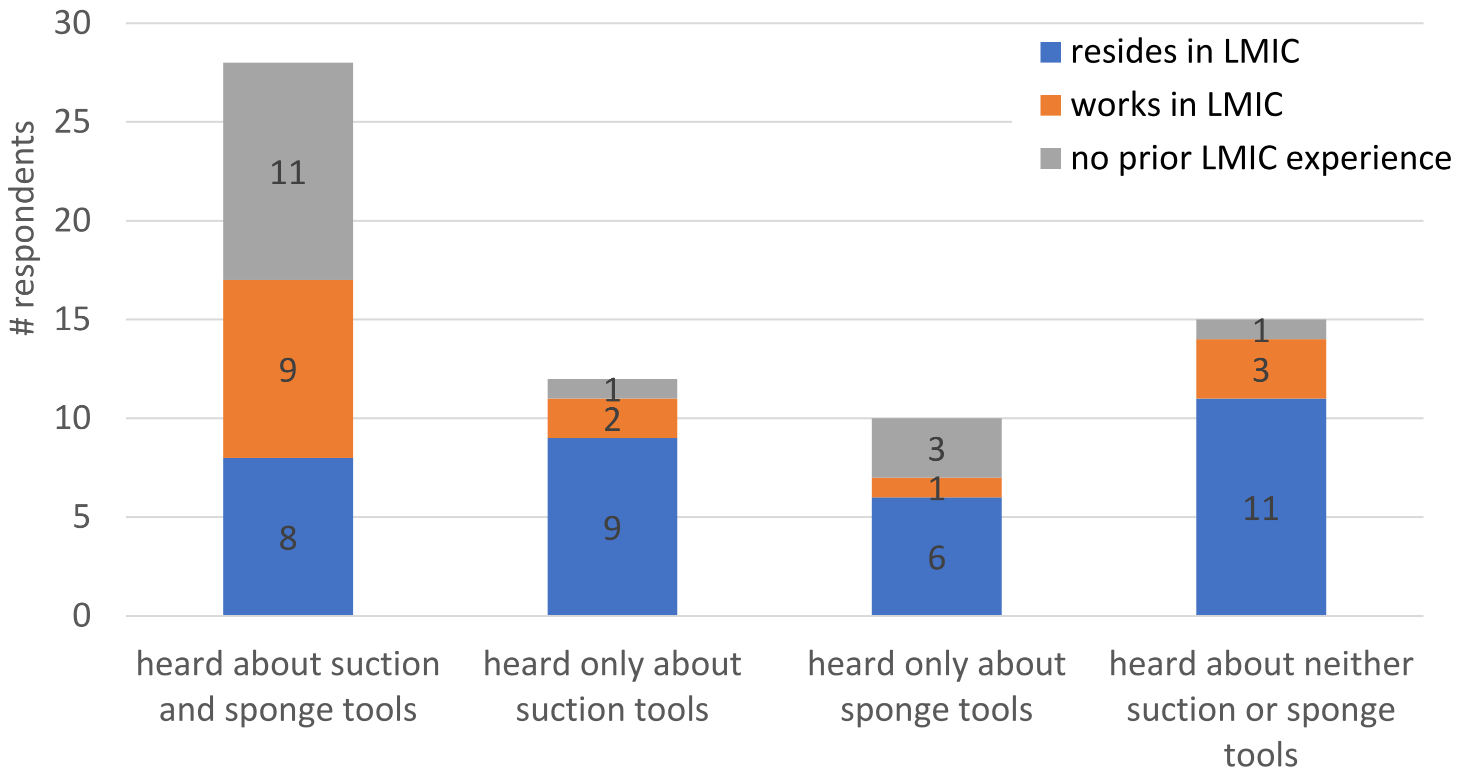

Perceived value of emerging tools

Perceived value among all respondents (n=65)

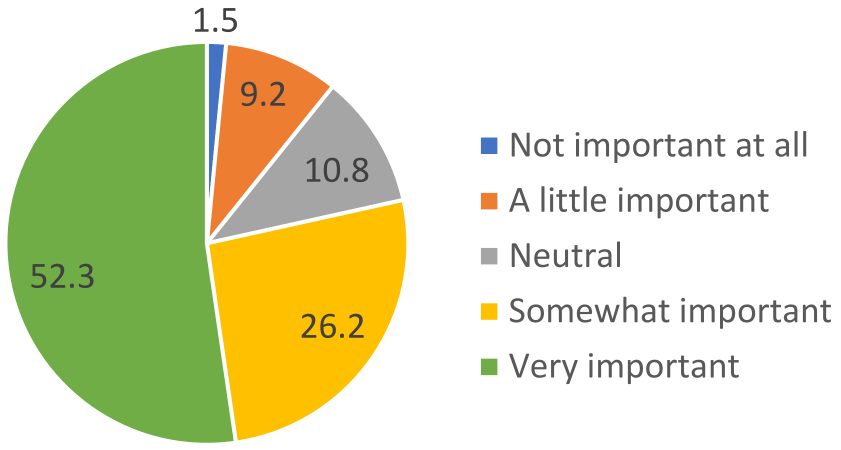

The majority of respondents regarded these tools as having a lot of potential.

Reason why these tools are somewhat or very important (n=51)

- Emerging tools show promise (n=23)
- Lack of effectiveness in certain cases (n=9)
- Lack of effectiveness in settings (n=7)
- Current tools are insufficient (n=6)
- Other (n=4)
- Conflicted data of existing tools (n=2)

Reason why these tools are not or a little important (n=7)

- Scale up of existing tools needed (n=3)
- New tools will be hard to scale/sustain (n=3)
- Other reason (n=1)

Perceived value among LMIC residents (n=34)

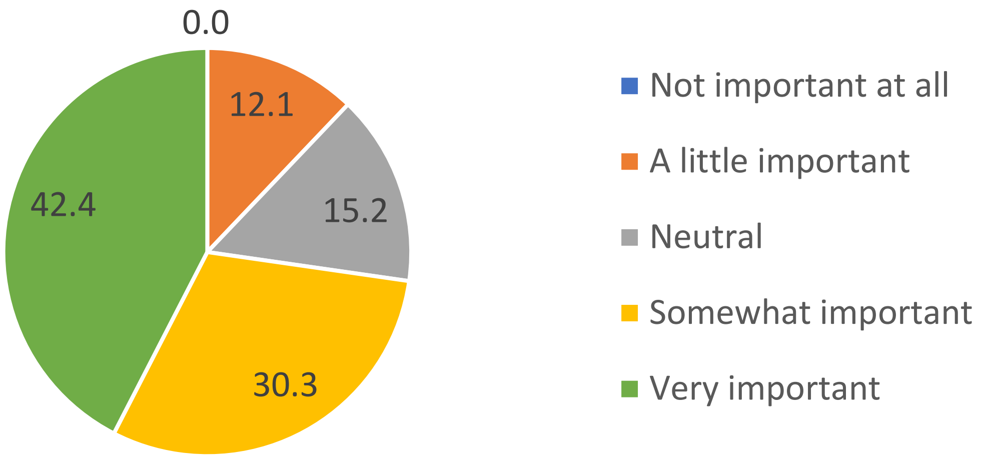

Perceived value of emerging tools

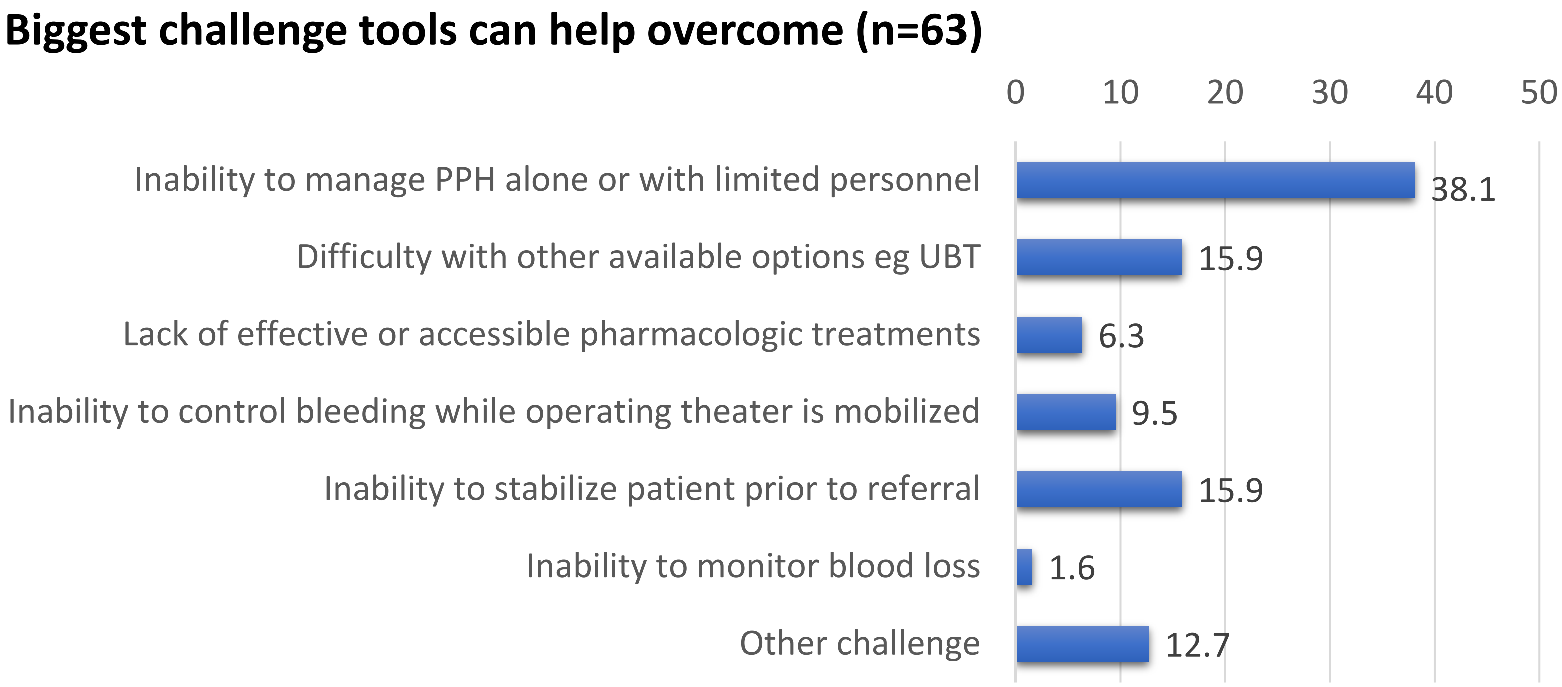

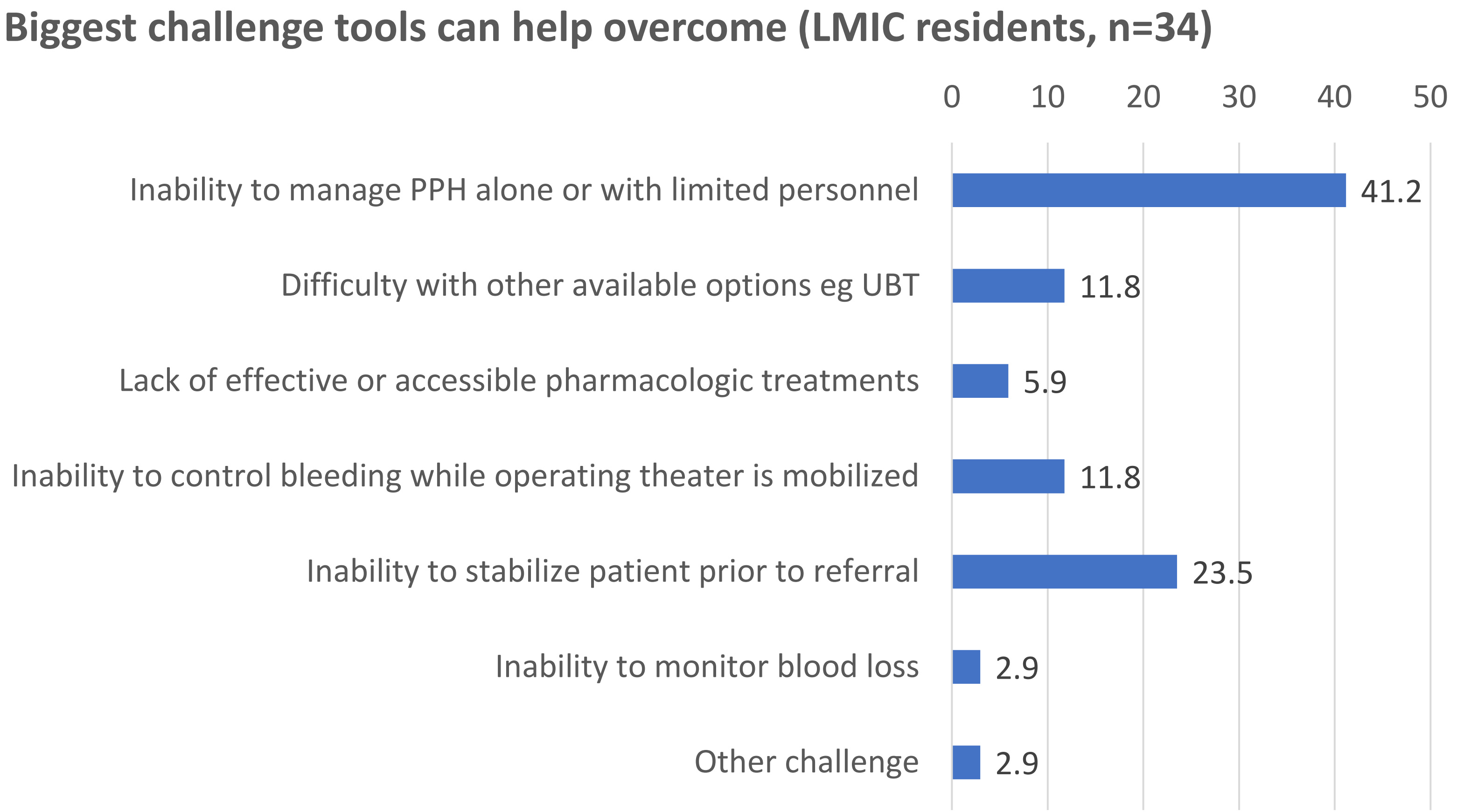

Many stated that they can help overcome issues around limited personnel, as well as stabilization prior to referral to a higher level of care. This suggests potential use at lower level facilities.

Recommended device use by provider cadre

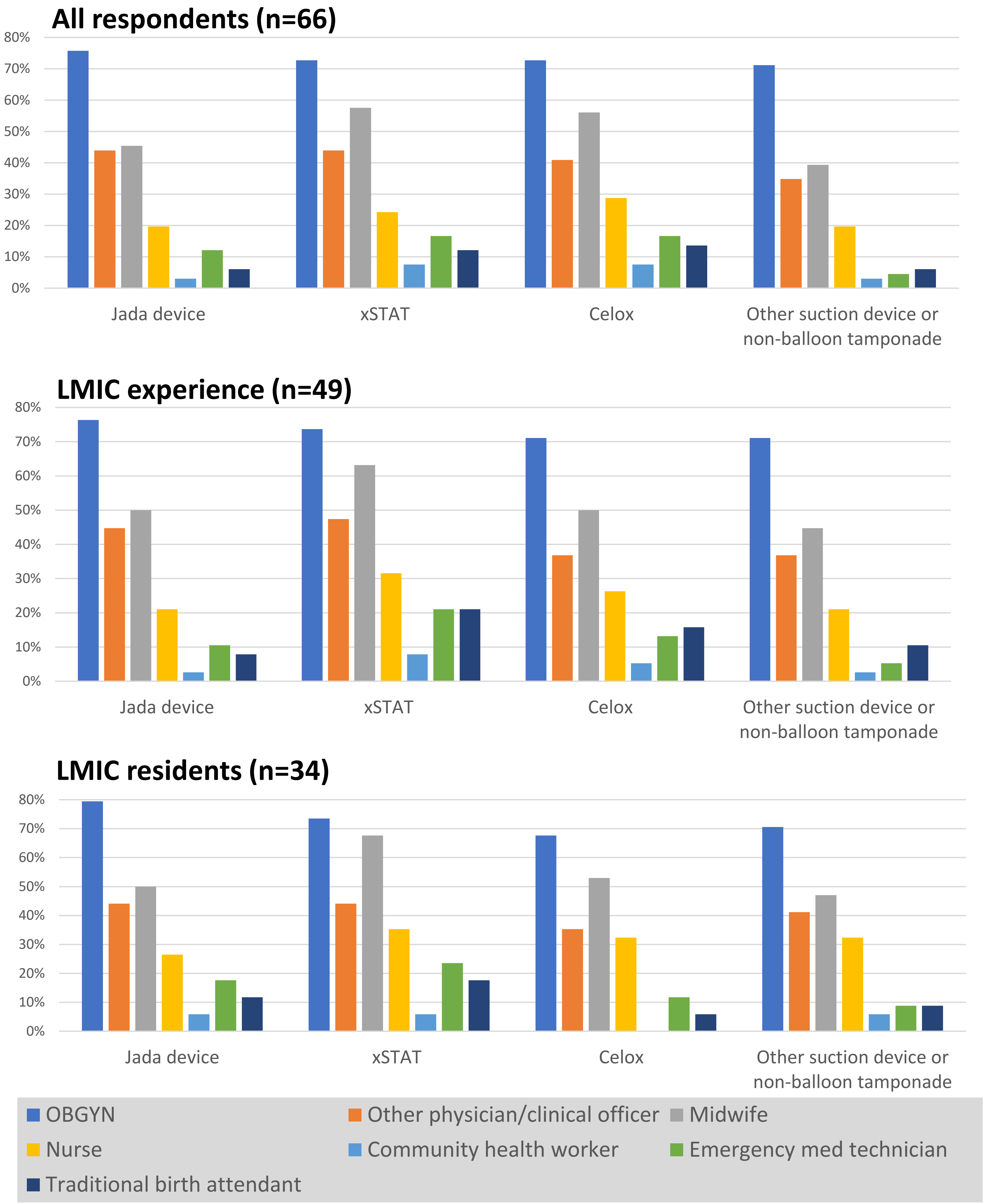

Overall provider impressions

- Among all providers, 34/50 (68%) would try one of these devices if they had access
- 5/50 (10%) would not; 11/50 (22%) did not answer

- Among LMIC providers, 25/38 (65.8%) would try one of these devices if they had access
- 4/38 (10.5%) would not; 9/38 (23.7%) did not answer

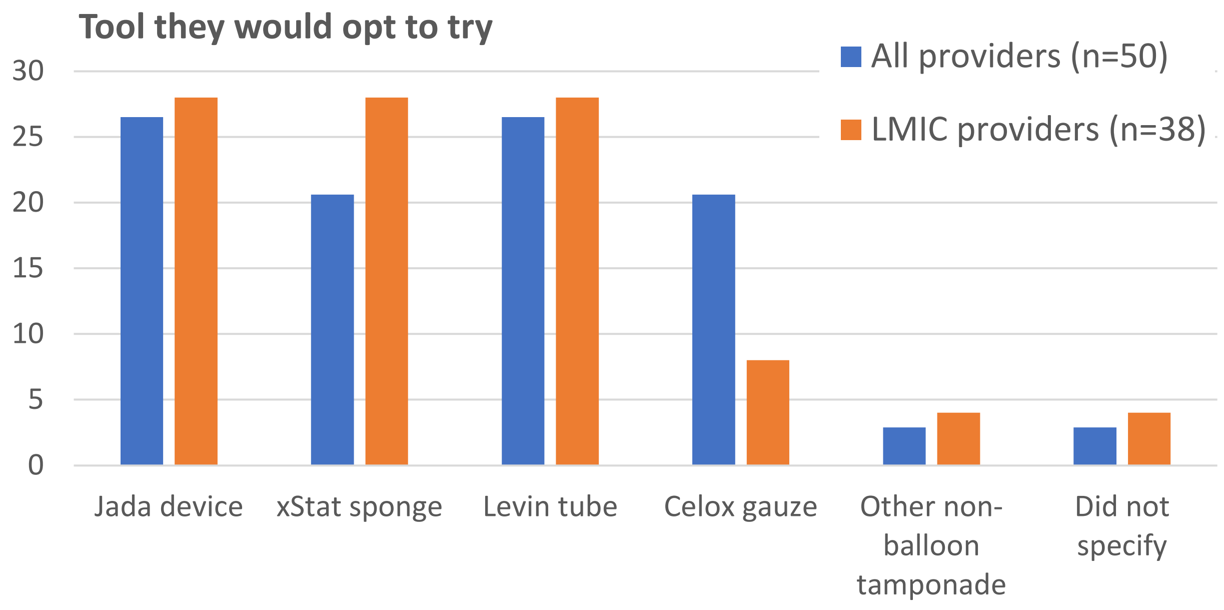

- Respondents largely think that these tools should be used by both OBGYNS/physicians and midwives. Fewer respondents felt they could be used by nurses.

- Higher proportion of respondents from LMICs and those with LMIC experience felt that XSTAT, Celox and other improvised suction/tamponade tools could be used by nurses or another lower level provider cadre.

Research prioritization

Detailed findings

Research prioritization | overall research focus

Survey prompt: When making a decision about introducing such a new tool in a LMIC setting, please rank the questions that must be answered in order of importance. *(1 = most important; 5 = least important).*

RANK	Research type	Mean	Stdev
**1**	**How does it impact PPH clinical outcomes in practice/real-world settings (effectiveness)?**	**1.37**	**0.641**
2	How does it impact PPH clinical outcomes in a highly controlled research environment (efficacy & safety)?	3.06	1.628
3	What is the provider experience, including ease of use (feasibility/acceptability)?	3.18	1.181
4	What is the patient experience, including pain, discomfort (feasibility/ acceptability)?	3.35	0.925
5	What other factors should be considered, such as cost, training/skill retention, supply chain, scalability (implementation research)?	3.62	1.300

Among LMIC residents, patient experience was slightly higher than provider experience (mean (SD) 3.26 (0.999) vs 3.29 (1.101)).

Research prioritization | device optimization

Survey prompt: What elements or characteristics of intrauterine devices/tools should be further explored in order to optimize or better understand their use? *(1 = most important; 5 = least important).*

RANK	Research type	Mean	Stdev
1	Adverse clinical outcomes	1.53	1.120
2	Standard procedures recommended with use	3.23	1.376
3	Length of time the device remains in place for treatment	3.58	1.419
4	Minimum suction required for effect	3.64	1.467
5	One-time use versus reusability	3.93	1.503
6	Prophylactic placement to prevent PPH among women with elevated risk	4.07	1.776

Among LMIC residents, rankings changed:

1. Adverse clinical outcomes
2. Length of time the device remains in place for treatment
3. Minimum suction required for effect
4. Standard recommendations with use
5. One-time use versus reusability
6. Prophylactic placement to prevent PPH among women with elevated risk

Research prioritization | provider perspective

Survey prompt: From providers' perspectives, what are priority topics to explore regarding introduction of these novel tools?
(1 = most important; 4 = least important).

RANK	Research type	Mean	Stdev
**1**	**Feasibility, ease of insertion and/or use**	**1.45**	**0.807**
2	Suitability of use by non-physician providers	2.47	0.935
3	Acceptability, resistance/ acceptance to change clinical practice	2.85	0.988
4	Effective training modalities to ensure and maintain competency	3.00	1.069

Ranking was identical among LMIC residents.

Research prioritization | women's perspective

Survey prompt: From women's perspectives, what are priority topics to explore regarding introduction of these novel tools? *(1 = most important; 5 = least important).*

RANK	Research type	Mean	Stdev
**1**	**Level of pain with placement and removal**	**2.26**	**1.262**
2	Level of pain throughout duration of treatment	2.85	1.133
3	Overall experience of care, including respectful care and communication	3.17	2.062
4	Acceptability, resistance/acceptance to device use	3.55	1.620
5	Length of insertion/treatment	4.09	1.378
6	Type of provider who should insert the device	4.58	1.592

Ranking was identical among LMIC residents.

Research prioritization | health system lens

Survey prompt: What health system factors should be further explored prior to implementation in order to optimize or better understand their adoption and uptake (scalability)? *(1 = most important; 5 = least important).*

RANK	Research type	Mean	Stdev
**1**	**Cost-effectiveness, including cost savings for averting subsequent care**	**2.42**	**1.560**
2	Product cost and implementation cost	3.00	1.715
3	Options for use in settings which lack electricity	3.30	1.659
4	Options for use during referral/transport to higher levels of care	3.49	1.230
5	Modifications of current PPH management algorithms to optimize integration and introduction	4.00	1.732
6	Requirements for supply chain and device maintenance	4.25	1.761

Among LMIC residents, requirements for supply chain/device maintenance was ranked higher than algorithm optimization.

Research prioritization key points

- Despite limited efficacy evidence regarding these tools (e.g., no randomized trials published to date), effectiveness research is the number one priority. This suggests that real-world circumstances and contextual factors are important to consider and prioritize when examining the potential for these tools.

- Many research areas were similarly ranked between respondents. LMIC respondents did prioritize issues related to supply chain/device maintenance and length of insertion compared to the larger sample, suggesting that these are important contextual factors. For example, shorter lengths of stay postpartum in LMICs may need to be accounted for when assessing feasibility and acceptability.

Designing an efficacy research study

Detailed findings

Efficacy research characteristics | Setting

Health system level appropriate for this study (all respondents, n=65)

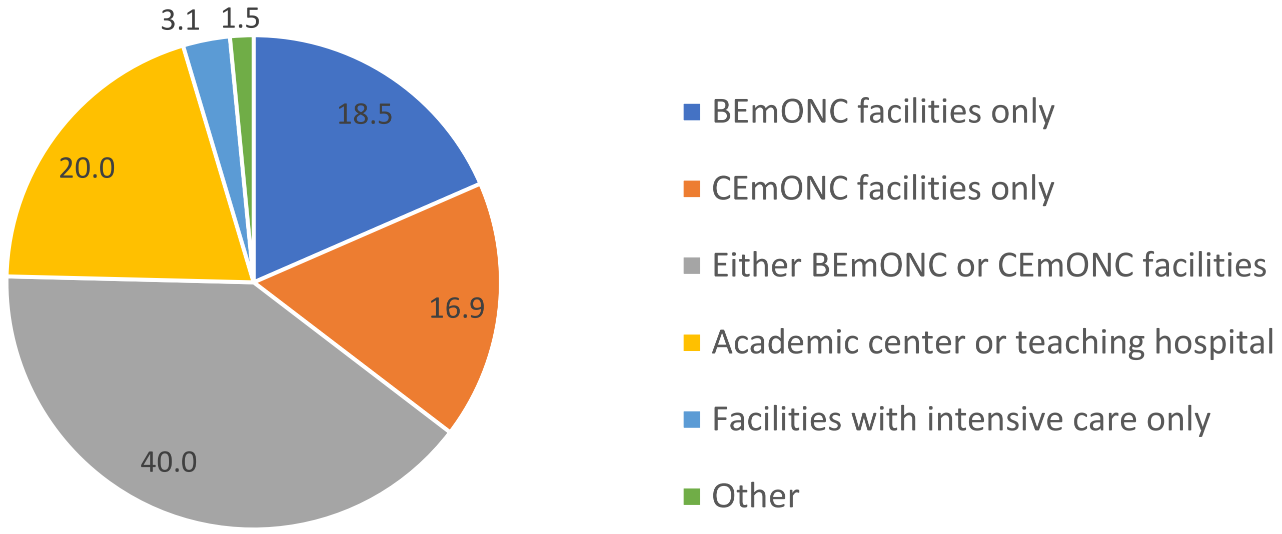

(LMIC experience, n=49)

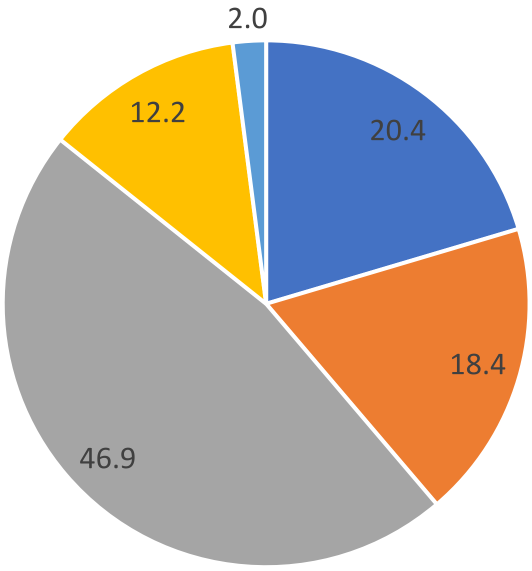

(LMIC residents, n=34)

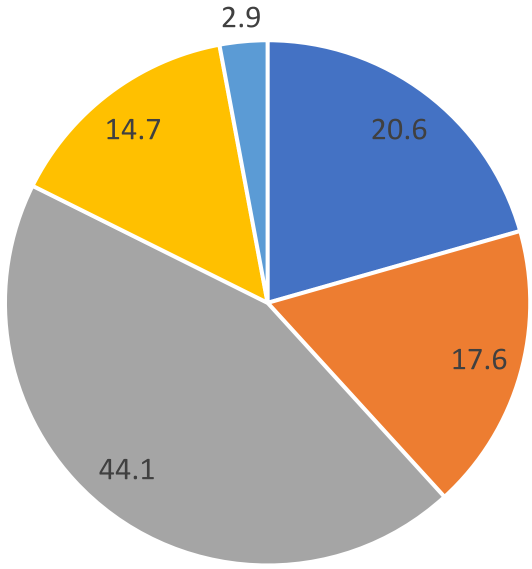

Efficacy study characteristics | Facility

Specific facility type (all respondents, n=64)

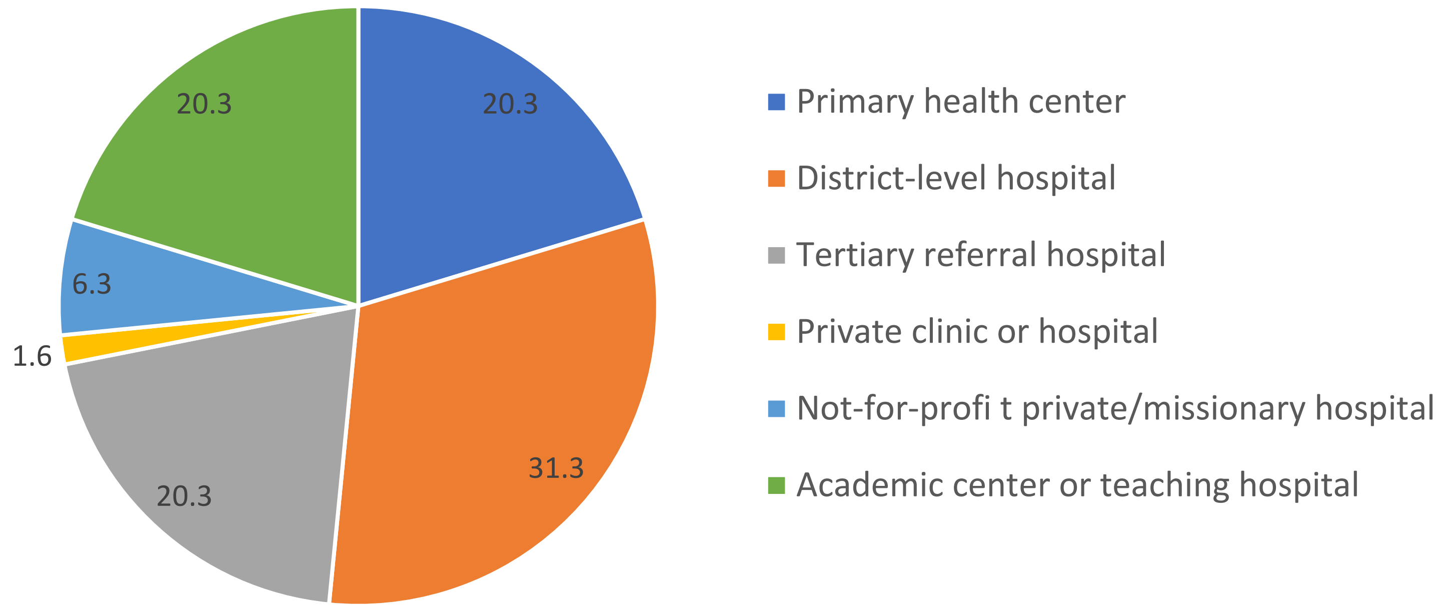

(LMIC experience, n=49)

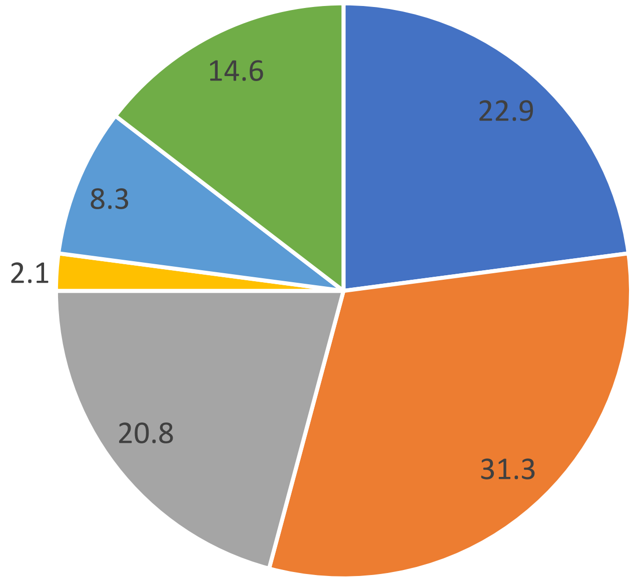

(LMIC residents, n=34)

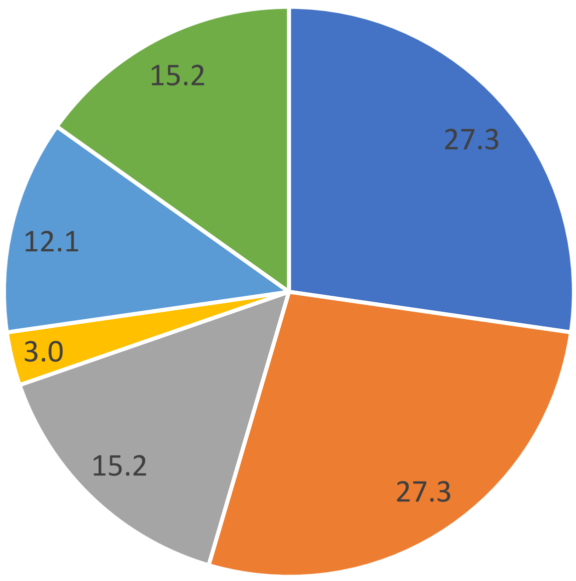

More LMIC residents felt studies should focus on primary health centers and not-for-profit missionary hospitals. Compared to the larger study sample, they prioritized tertiary referral hospitals and district level hospitals less.

Necessary facility characteristics that should be in place at study site

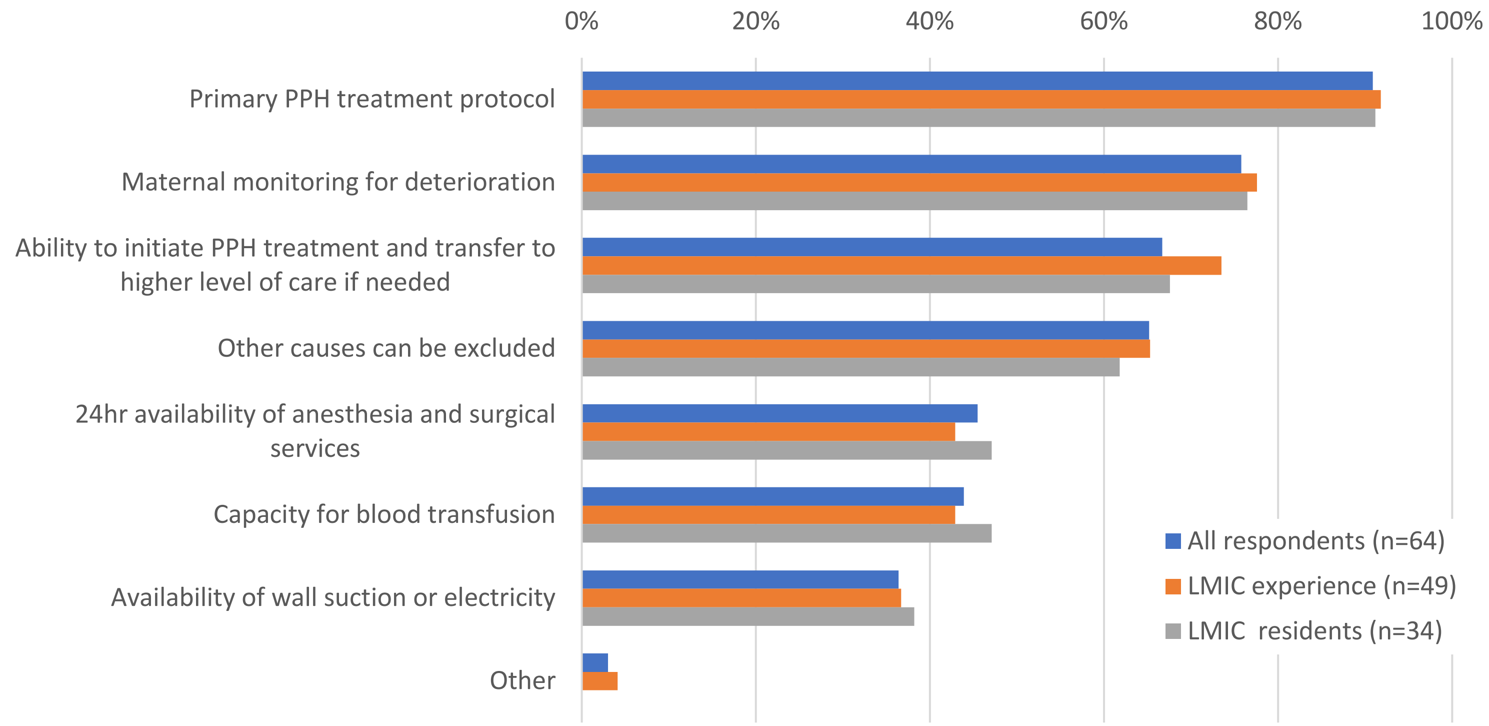

The top 4 necessary facility characteristics rated across all sub-groups should be present at BEmONC facilities.

Necessary facility characteristics that should be in place at study site	All respondents (n=64)	LMIC experience (n=49)	LMIC residents (n=34)
Primary PPH treatment protocol	90.9%	91.8%	91.2%
Maternal monitoring for deterioration	75.8%	77.6%	76.5%
Ability to initiate PPH treatment and transfer to higher level of care if needed	66.7%	73.5%	67.6%
Other causes can be excluded	65.2%	65.3%	61.8%
24hr availability of anesthesia and surgical services	45.5%	42.9%	47.1%
Capacity for blood transfusion	43.9%	42.9%	47.1%
Availability of wall suction or electricity	36.4%	36.7%	38.2%
Other	3.0%	4.1%	0.0%

Elements that would require strengthening prior to study initiation

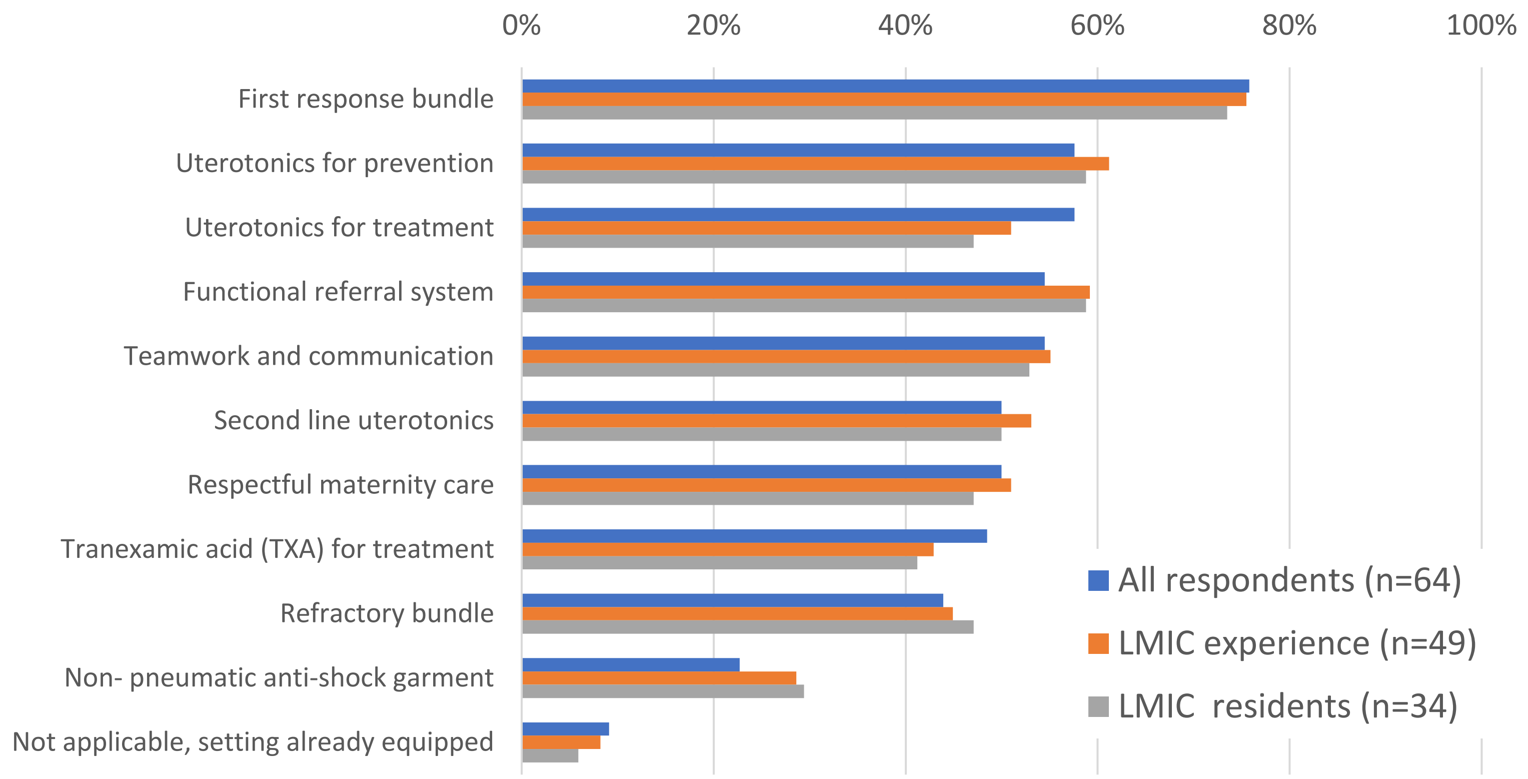

Elements that would require strengthening prior to study initiation	All respondents (n=64)	LMIC experience (n=49)	LMIC residents (n=34)
First response bundle	75.8%	75.5%	73.5%
Uterotonics for prevention	57.6%	61.2%	58.8%
Uterotonics for treatment	57.6%	51.0%	47.1%
Functional referral system	54.5%	59.2%	58.8%
Teamwork and communication	54.5%	55.1%	52.9%
Second line uterotonics	50.0%	53.1%	50.0%
Respectful maternity care	50.0%	51.0%	47.1%
Tranexamic acid (TXA) for treatment	48.5%	42.9%	41.2%
Refractory bundle	43.9%	44.9%	47.1%
Non- pneumatic anti-shock garment	22.7%	28.6%	29.4%
Not applicable, setting already equipped	9.1%	8.2%	5.9%

Efficacy study | Provider cadre

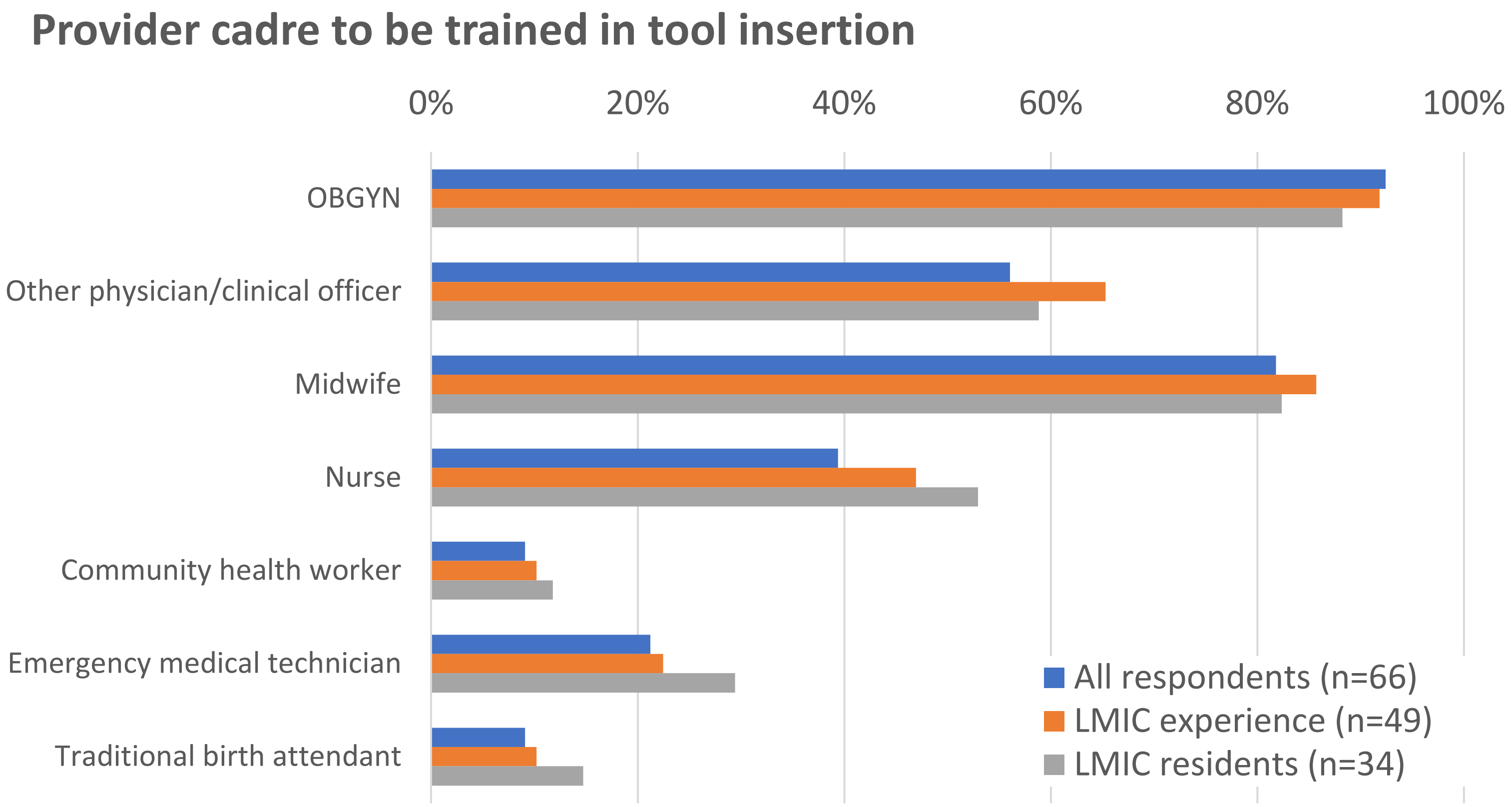

Although OBGYN and midwives were the preferred provider cadre to train, more LMIC residents felt nurses and lower cadre providers should also be trained to insert these tools.

Provider cadre	All respondents (n=66)	LMIC experience (n=49)	LMIC residents (n=34)
OBGYN	92.4%	91.8%	88.2%
Other physician/clinical officer	56.1%	65.3%	58.8%
Midwife	81.8%	85.7%	82.4%
Nurse	39.4%	46.9%	52.9%
Community health worker	9.1%	10.2%	11.8%
Emergency medical technician	21.2%	22.4%	29.4%
Traditional birth attendant	9.1%	10.2%	14.7%

Efficacy study | Timing of use

Step after which tool should be inserted

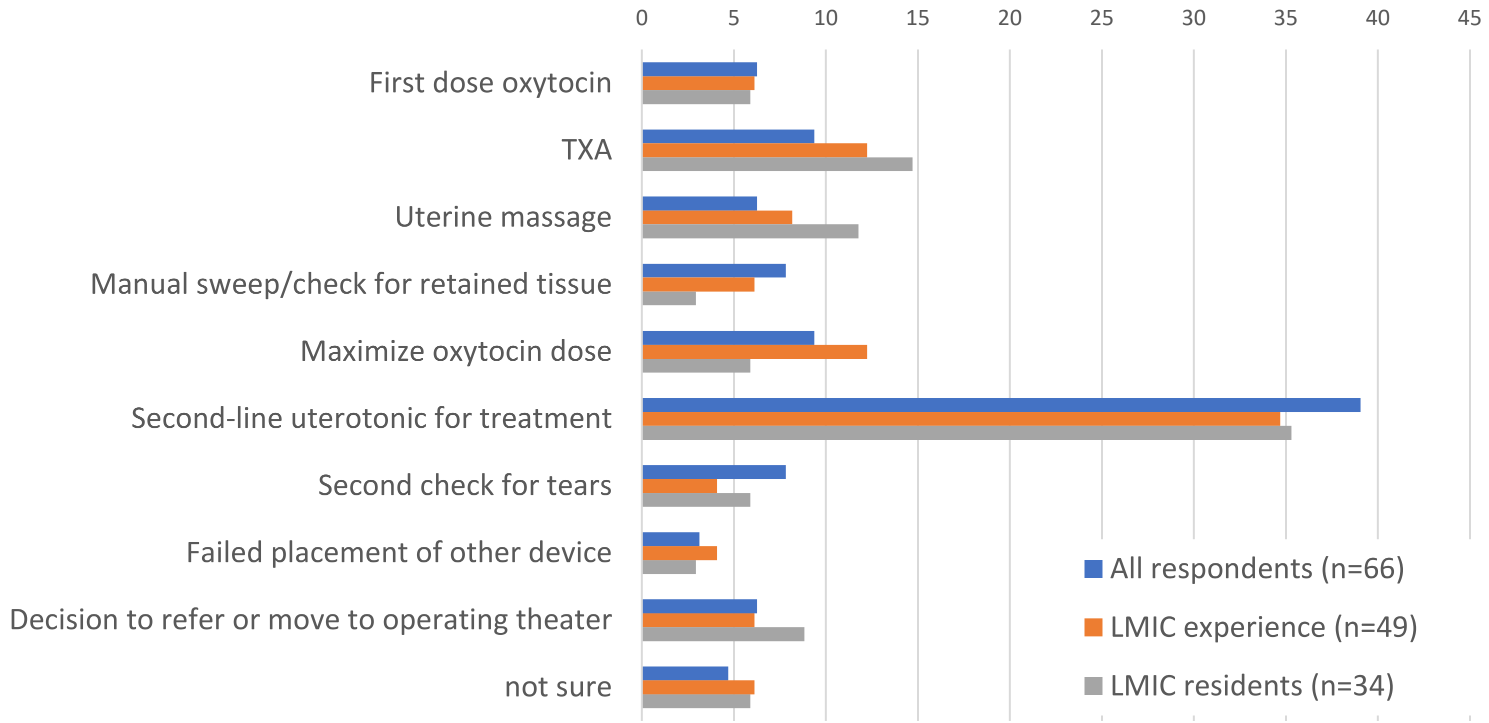

Majority of respondents felt that these tools should be used after second-line uterotonics are given. This begs the question of how long to wait after uterotonics are administered.
A higher proportion of LMIC-experienced respondents (45%) recommended use prior to second-line uterotonics compared to HIC respondents (21%).

**Step after which tool should be inserted**	**All respondents (n=66)**	**LMIC experience (n=49)**	**LMIC residents (n=34)**
First dose oxytocin	6.3	6.1	5.9
TXA	9.4	12.2	14.7
Uterine massage	6.3	8.2	11.8
Manual sweep/check for retained tissue	7.8	6.1	2.9
Maximize oxytocin dose	9.4	12.2	5.9
Second-line uterotonic for treatment	39.1	34.7	35.3
Second check for tears	7.8	4.1	5.9
Failed placement of other device	3.1	4.1	2.9
Decision to refer or move to operating theater	6.3	6.1	8.8
not sure	4.7	6.1	5.9

Efficacy study | Intervention & comparator

Among all respondents (n=61)		CONTROL GROUP: UBT	CONTROL GROUP: Standard care	CONTROL GROUP: Other	Total
INTERVENTION GROUP	Jada device	8	6	1	15 (24%)
	XSTAT sponge	13	11	2	26 (43%)
	Levin stomach tube	2	5	0	7 (11%)
	Vacuum-induced Bakri	1	2	0	3 (5%)
	Celox gauze	3	6	0	9 (15%)
	Other	0	1	0	1 (2%)
	Total	27 (44%)	31 (51%)	3 (5%)	61

Among respondents with LMIC experience (n=49)		CONTROL GROUP: UBT	CONTROL GROUP: Standard care	CONTROL GROUP: Other	Total
INTERVENTION GROUP	Jada device	8	5	0	13 (28%)
	XSTAT sponge	11	9	1	21 (46%)
	Levin stomach tube	2	3	0	5 (11%)
	Vacuum-induced Bakri	1	2	0	3 (7%)
	Celox gauze	1	2	0	3 (7%)
	Other	0	1	0	1 (2%)
	Total	23 (50%)	22 (48%)	1 (2%)	46

LMIC residents (n=34)		CONTROL GROUP: UBT	CONTROL GROUP: Standard care	Total
INTERVENTION GROUP	Jada device	7	4	11 (34%)
	XSTAT sponge	9	5	14 (44%)
	Levin stomach tube	2	1	3 (9%)
	Vacuum-induced Bakri	0	1	1 (3%)
	Celox gauze	1	1	2 (6%)
	Other	0	1	1 (3%)
	Total	19 (59%)	13 (41%)	32

Various permutations of intervention vs. control were selected. XSTAT vs. UBT was the most frequent combination, though overall numbers were small.

Efficacy study | Intervention & setting

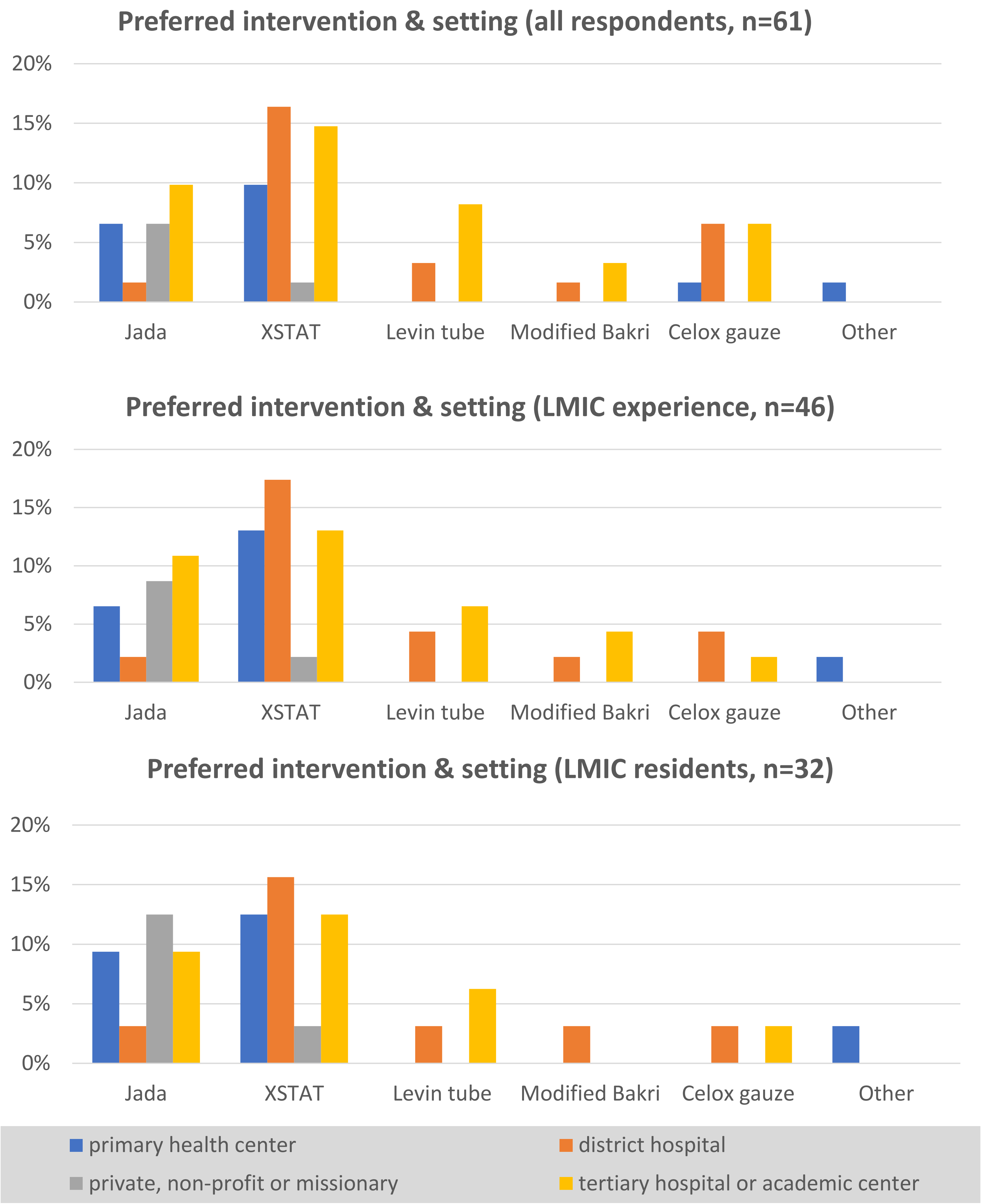

Efficacy study | Primary outcome

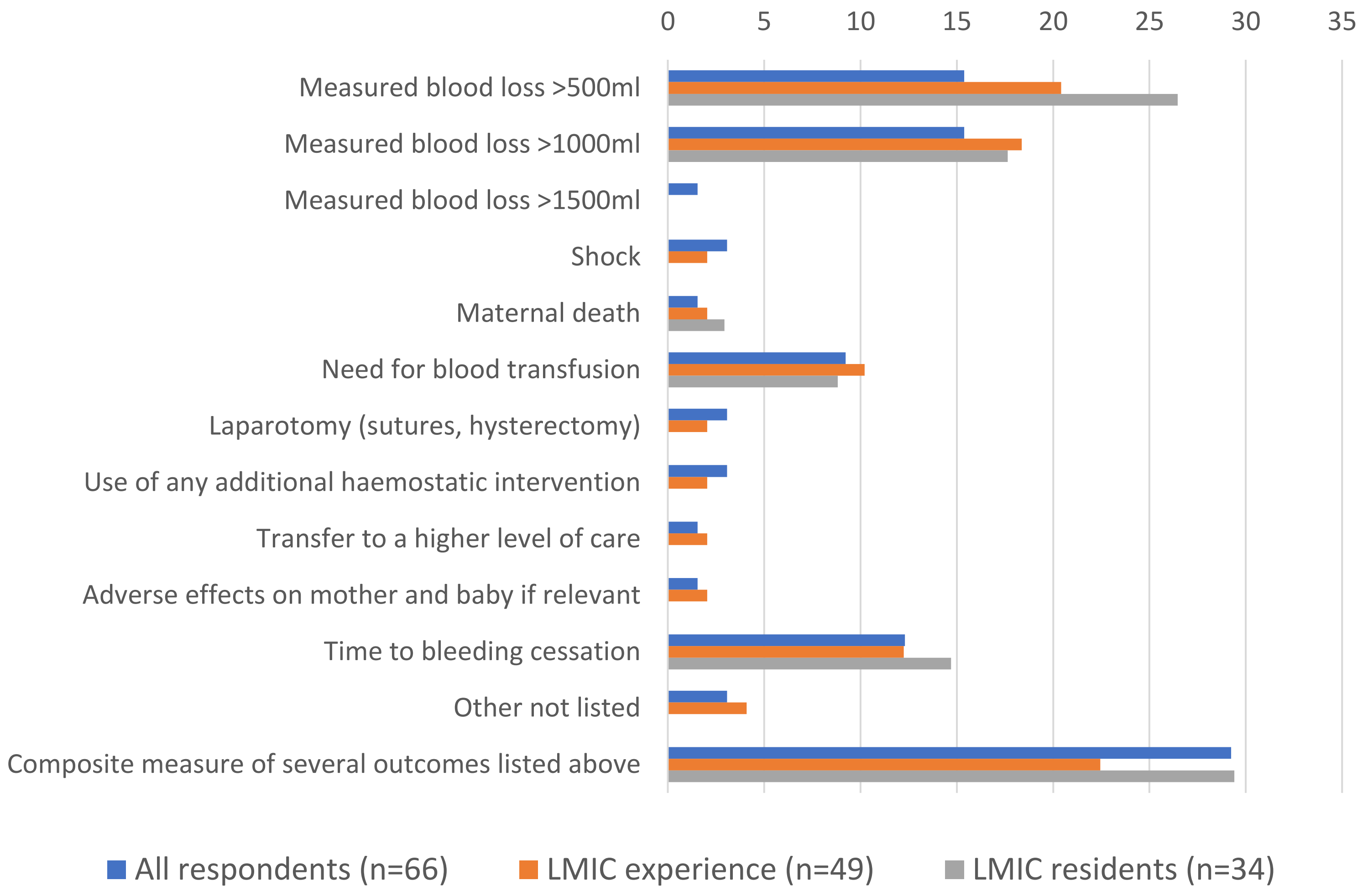

Efficacy study | Composite outcome components

Measures to include in a composite outcome	% (n=19)
Need for blood transfusion	68.4%
Shock	68.4%
Maternal death	63.2%
Time to bleeding cessation	57.9%
Measured blood loss >1000ml	52.6%
Laparotomy (sutures, hysterectomy)	52.6%
Admission to intensive care unit	47.4%
Measured blood loss >1500ml	36.8%
Use of any additional hemostatic intervention	36.8%
Transfer to a higher level of care	36.8%
Any organ dysfunction	36.8%
Post-procedural complication	31.6%
Coagulopathy	26.3%
Adverse effects on mother and baby if relevant	26.3%
Measured blood loss >500ml	21.1%
Breastfeeding	5.3%

Efficacy study | Secondary outcomes

Maternal clinical outcomes

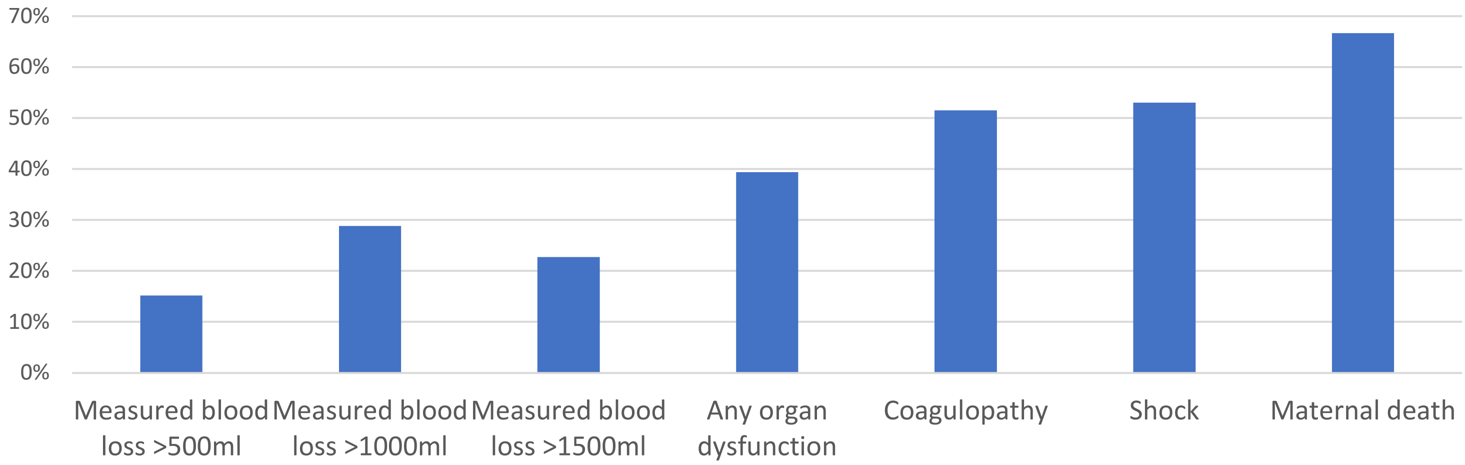

Clinical pathway/intervention

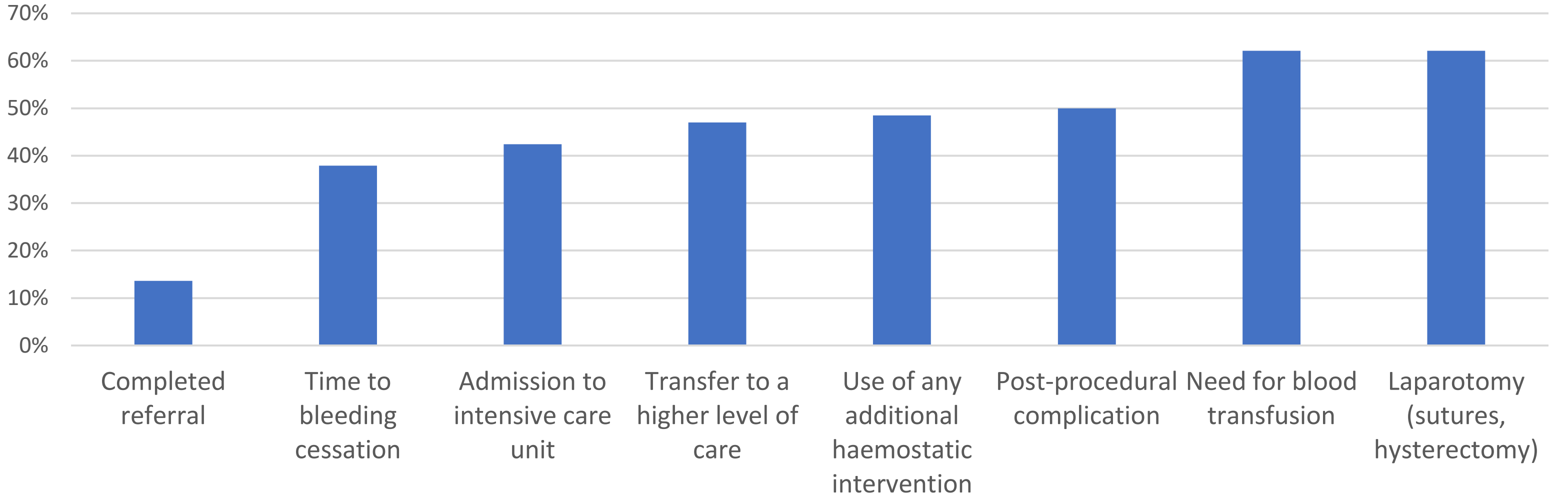

Other maternal or newborn outcome

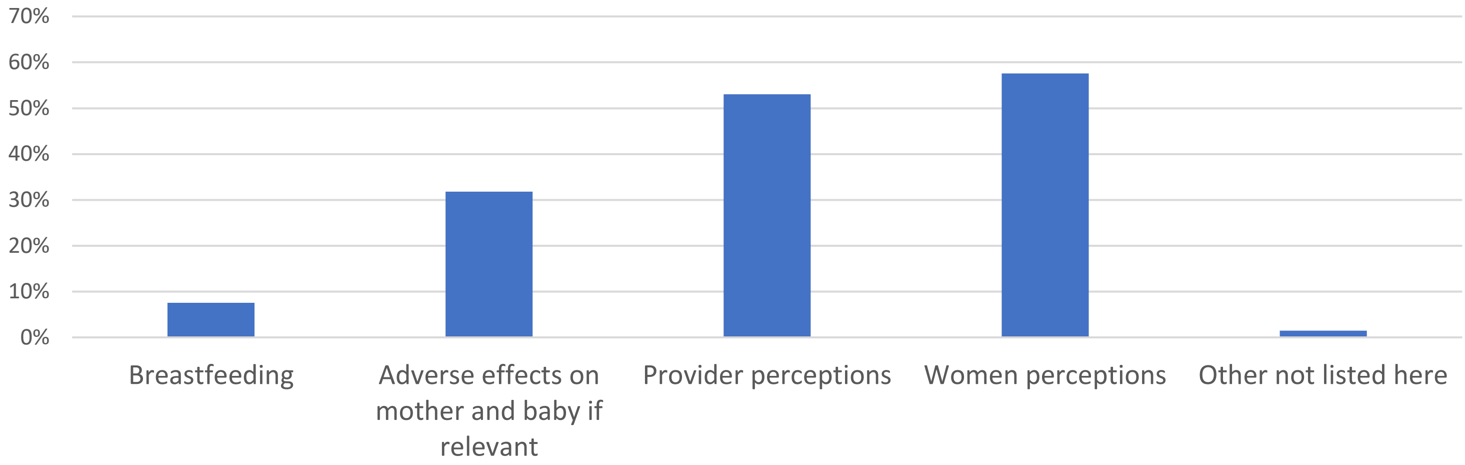

Limitations

- This survey has a small sample size, with most respondents identifying as a healthcare provider, specifically OBGYNs. This could skew results with specialist end-user perspectives. We lacked adequate representation from midwives and nurses, as well as other groups such as policymakers and implementors.
- Although we attempted to share the surveys broadly, our method of snowball dissemination may have increased potential for selection bias for people who are already familiar with certain tools/approaches.
- The survey presented a suite of emerging tools and then asked general questions about perceptions and knowledge. Given the heterogeneity of existing tools as well as individual’s background/experience, it is unclear if respondents answered these questions with a specific tool in mind.
- The survey was conducted online using a forced choice format, particularly when asked to rank research priorities or choose specific tools/methods to test. This format may have influenced respondents’ comfort in completing individual questions. However, we note few missing data across questions.
