# Supplementary material for: Global research priorities for intrauterine suction and sponge tools for postpartum haemorrhage management in low-income and middle-income countries: a modified Delphi approach
Source: BMJ Public Health. 2024 May 30;2(1):e000113. doi: 10.1136/bmjph-2023-000113 (PMC11812741; doi:10.1136/bmjph-2023-000113)
Supplement: online supplemental file 7 [file bmjph-2-1-s007.pdf]

EMERGING TOOLS FOR PPH MANAGEMENT: PRODUCT PROFILE SURVEY

Summary of results (draft)

July 2022

TABLE OF CONTENTS

Description	Pages
Summary	3
Methods	4 – 5
Study sample	6
Key findings	7 – 12
Detailed results	13
Demographics	14 – 17
Characteristics	18 – 32
Limitations	33

Summary

Objective:

We surveyed respondents through targeted outreach and snowball sampling to determine what characteristics should be considered essential and desirable for new tools and technologies emerging for the management of postpartum hemorrhage (PPH).

The survey asked respondents to rate the importance of tool characteristics, grouped across 15 characteristics domains focusing on the use and implementation of these tools in the respondents' setting. At the end of the survey, respondents were asked about which characteristics were most and least important.

Participants:

Respondents included 89 respondents of whom 51 were from low- and middle-income countries (LMICs) and the remaining 38 were from high-income countries (HICs). Health providers represented 95% of HIC respondents and 61% of LMIC respondents. Nearly all HIC providers were OB/GYNs, whereas LMIC providers included other physicians or clinical officers, midwives, and nurses.

Findings:

Respondents rated efficacy for limiting blood loss and prevention of surgery, clinical indication, cost, and timing of use as the top five characteristics of interest.

Characteristics which elicited consensus of 75% among all respondents rating them as essential include:

- Can be used for uterine atony following vaginal or cesarean birth
- Can be placed by OB/GYNs
- Can be used in district and tertiary hospitals

Additionally, at least 65% of LMIC respondents felt the following were essential:

- Can be used when bleeding continues after initial response
- Can be used in a private clinic or hospital
- Can be inserted by a medical officer or clinical officer
- Can be inserted by a midwife
- Works at least as well as current standard of care to avoid surgery with no increased risk
- In-person training with simulation/models, or ongoing proctoring or mentoring
- Can be inserted by a single provider
- Can be inserted quickly (<2 min)
- Can be stored at ambient temperature
- Costs less than USD$10/patient

Comparing these characteristics to what is known about emerging tools will aid in the identification of research priorities.

Methods

Context:

The Product Profile Survey was deployed as part of an overarching project to understand and arrive at research priorities for intrauterine, non-balloon suction and sponge devices for the management of PPH. The project included a literature review, in-depth interviews with key informants, and surveys to understand desired product characteristics and research priorities. This work will culminate in an expert convening which will be documented with a white paper, research roadmap, Target Product Profile, or other summary product.

Survey Development and Deployment:

Survey questions were developed based on literature review of existing tools and insights from key informant interviews. The Product Profile survey included questions about tool characteristics grouped across 15 characteristics domains. Respondents were asked to rate characteristics as essential, desirable or not important. An option was included for those who had no opinion on any given characteristic. At the conclusion of the survey, respondents were asked to rate which characteristics were most and least important.

Surveys were emailed out to professional networks of colleagues working in safe motherhood, PPH, and related topics. Key researchers from publications related to new tools were also included, and all respondents were encouraged to share survey links with their own professional networks. Surveys were accessed via a link and completed in REDCap on a UCSF server. The survey link was left open for one month, with weekly reminders sent to email recipients.

Analysis:

Survey data were analyzed using SPSS. We compiled frequencies and crosstabs separating respondents by HIC or LMIC setting. Frequencies were converted into stacked bar charts, and results reviewed for consensus. Consensus was defined as 75% of respondents from all settings agreeing that a characteristic was essential. Characteristics that failed to meet the essential threshold but had at least 75% of respondents designate them as either essential or desirable were classified as desirable. Results among LMIC respondents only were also examined to determine if any characteristics that did not meet thresholds for the full sample, were met among this subgroup using a 75% threshold and an alternate 65% threshold. Characteristics were ranked using a composite score that combined the proportion of respondents designating a characteristic among the most important and the inverse proportion of those designating it as among the least important characteristics.

A draft Target Product Profile was created using the characteristics identified as essential or desirable. Emerging products were compared to this profile for characteristics meeting the essential threshold among all respondents or LMIC respondents only, using 75% and 65% thresholds, respectively.

How we determined consensus on tool characteristics

To be consistent with other Target Product Profiles available online, we selected 75% as the threshold for consensus. Respondents were able to select only one choice (essential, desirable, not important or no opinion), so for characteristics that did not meet the consensus threshold for essential we combined essential plus desirable responses for the desirable threshold.

- **Essential**: ≥ 75% of all respondents concurred that the characteristic was essential
  - In the example below, tertiary and district hospitals are considered essential settings for use.

- **Desirable:** ≥ 75% of all respondents stated that the characteristic was essential or desirable (i.e., essential %+ desirable %)
  - In the example below, private clinics/hospitals, during transport, and primary health centers are considered desirable settings for use

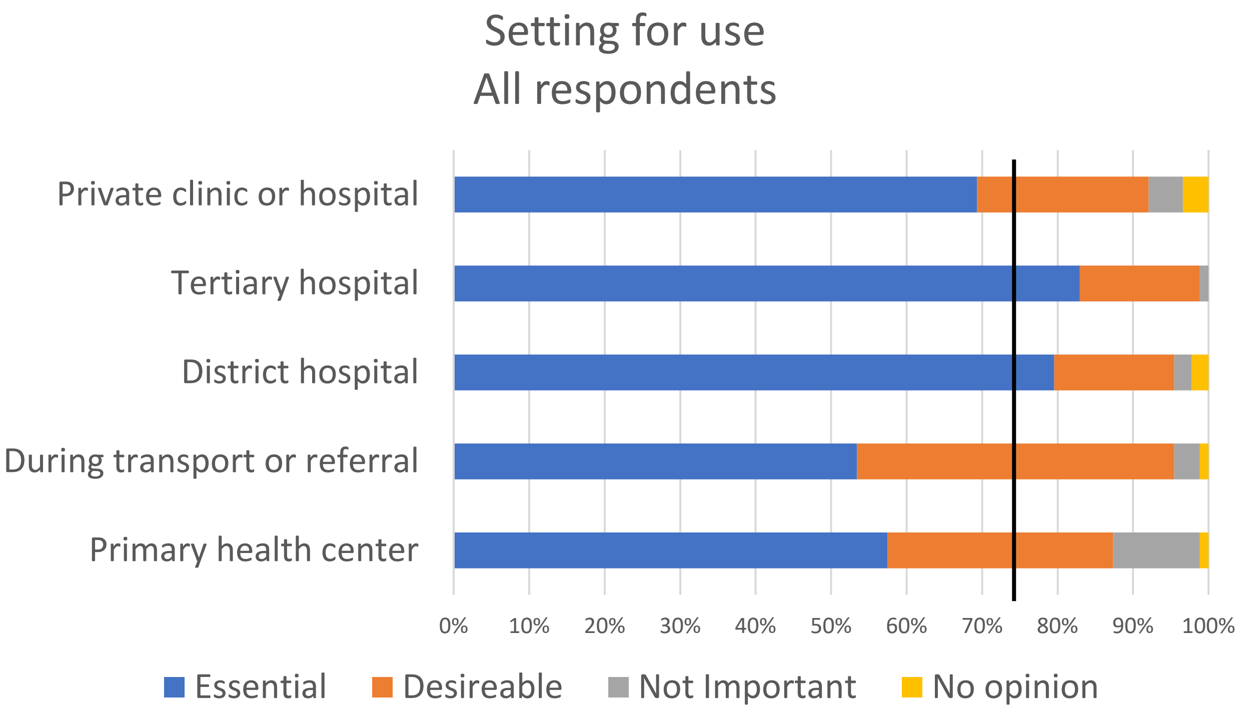

Study Sample

We sent an email with a link to the survey to approximately 500 individuals known to be researchers, healthcare providers, program implementors, or policy makers involved in PPH care. Of the 97 individuals who initiated the survey, 89 completed it, over half of whom were from LMICs.

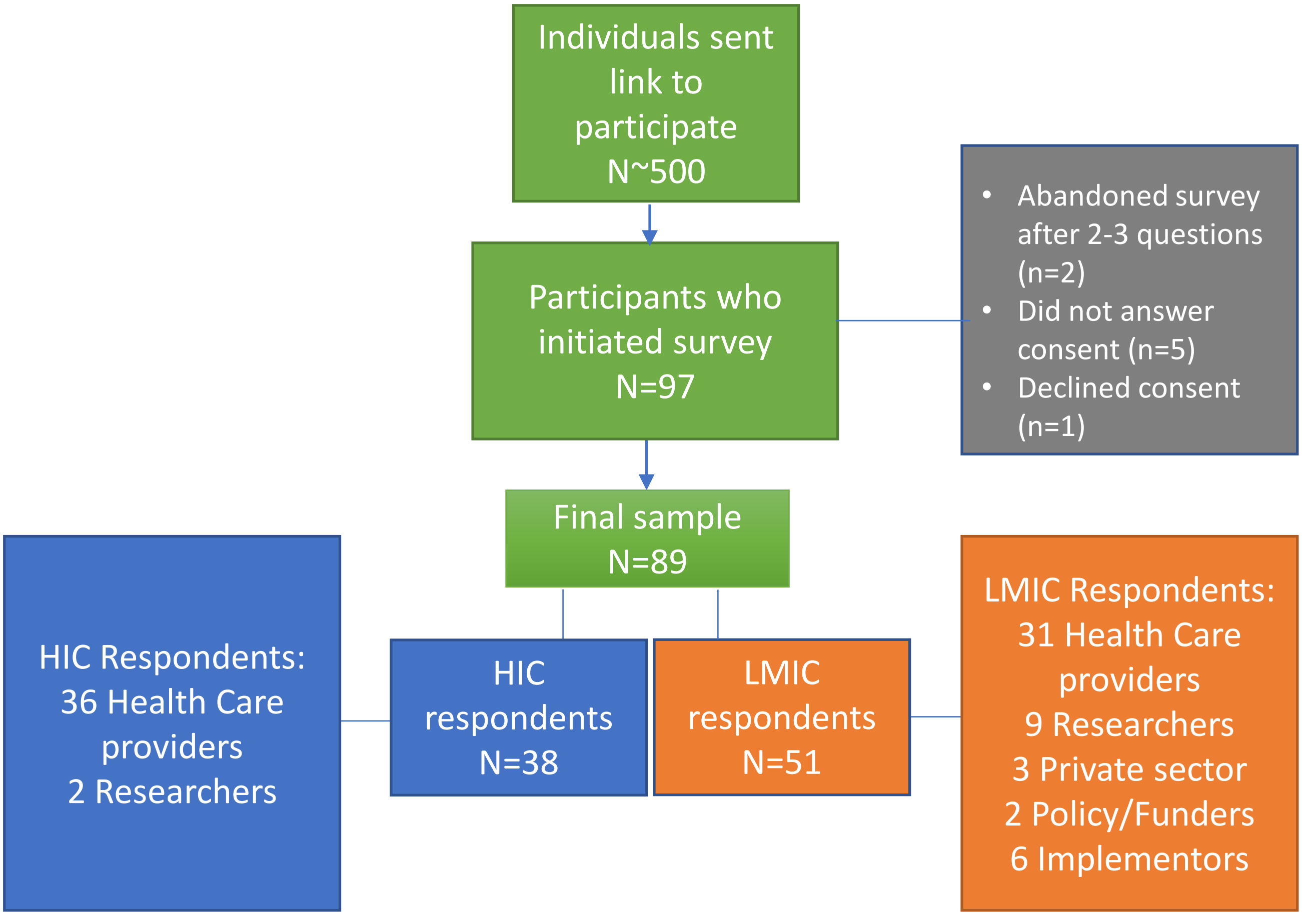

KEY FINDINGS

Considerations ranked by importance

The tables below show the relative rankings of the fourteen consideration categories as ranked by participants, who were asked to select the 6 most important and 6 least important considerations. We analyzed rankings for all respondents as well as examining those of LMIC respondents only. Of note, the top five and bottom three closely aligned between respondent groups. There was more variability around the middle of the list with reusability and provider cadre of higher importance to LMIC respondents.

ALL RESPONDENTS	
1	Efficacy/risk - prevention of blood loss >1000ml
2	Efficacy/risk - prevention of surgical intervention
3	Clinical indication (type of PPH)
4	Product cost
5	Timing of use during PPH management
6	Storage characteristics
7	**Patient experience**
8	Provider proficiency
9	Reuseability
10	Training mechanism
11	Provider cadre
12	Treatment duration
13	Regulatory considerations
14	Facility type

LMIC RESPONDENTS	
1	Efficacy/risk - prevention of blood loss >1000ml
2	Clinical indication (type of PPH)
3	Efficacy/risk - prevention of surgical intervention
4	Product cost
5	Timing of use during PPH management
6	**Reuseability**
7	Storage characteristics
8	Provider proficiency
9	Provider cadre
10	Patient experience
11	Training mechanism
12	Facility type
13	Treatment duration
14	Regulatory considerations

Note: ease of use was inadvertantly omitted from the ranking list.

DRAFT Target Product Profile (1 of 2)

The table below presents the essential and desirable characteristics for all respondents using the 75% threshold previously described.

Characteristic	Essential	Desirable
**Efficacy/Risk to prevent blood loss >1000ml**	No essential efficacy threshold identified	If it works at least as well as standard of care with no more than minimal increased risk
**Clinical Indication**	Uterine atony following vaginal birth<br>Uterine atony following cesarean section	Trauma to the genital tract<br>Placental implantation abnormality<br>Coagulopathy
**Efficacy/Risk to Prevent Surgical Intervention**	No essential efficacy threshold identified	If it works at least as well as standard of care with no more than minimal increased risk
**Product Cost**	No essential threshold identified	Product should be <US$10/patient
**Timing of Use**	No essential timing identified	When bleeding continues after initial response<br>When all other options have been exhausted
**Reusability**	No essential characteristic identified	Parts or all of the product can be sterilized and reused
**Storage Characteristics**	No essential characteristic identified	Able to be stored at ambient temperature<br>Able to withstand high ambient temperatures<br>Cold chain storage is NOT desirable

DRAFT Target Product Profile (2 of 2)

Characteristic	Essential	Desirable
**Provider Proficiency**	No essential threshold identified	2-5 insertions for competency
**Provider Cadre**	OBGYNs	Medical officers or clinical officers<br>Midwives<br>Emergency Care technician<br>Nurses
**Patient Experience**	No essential experience identified	Patient experience similar to or better than standard of care
**Training Mechanism**	No essential characteristic identified	All<br>In-person training with simulation/models<br>In-person training preferred in LMICs
**Facility Type**	Tertiary hospitals<br>District hospitals	Primary health centers<br>During transport or referral<br>Private clinic or hospital
**Treatment Duration**	No essential duration identified	Treatment duration comparable to or shorter than standard of care
**Regulatory Considerations**	No specific regulatory condition identified as essential	Product approval for PPH or another indication is desirable

Views of LMIC respondents diverged on some key points

We analyzed the subset of LMIC respondents only and found that an additional 5 characteristics were identified as essential by at least 75% of that subset. Further, if we drop the threshold to 65% for essential characteristics, an additional 6 characteristics emerge as important to LMIC respondents.

At least 75% of LMIC respondents felt the following were essential characteristics:

- Can be used in a private clinic or hospital (86%)
- Can be used when bleeding continues after initial response (82%)
- In-person training with simulation/model (80%)
- Can be inserted by a midwife (78%)
- Can be inserted by a medical officer or clinical officer (77%)

Additionally, at least 65% of LMIC respondents felt the following were essential:

- Costs less than USD$10/patient (74%)
- In-person training with ongoing proctoring or mentoring (74%)
- Can be stored at ambient temperature (72%)
- Can be inserted by a single provider (68%)
- Can be inserted quickly (<2 min) (68%)
- Works at least as well as current standard of care to avoid surgery with no increased risk (65%)

Key messages

Based on the responses from this heterogeneous group of respondents, the following messages emerge:

- Tools that can prevent surgery or blood loss greater than 1000ml are valued, particularly for uterine atony, provided no added risk
- Products should be inexpensive, storable at ambient temperature
- Emerging tools need to consider end-user needs
- Different tools might meet different needs (i.e. suction devices in district or tertiary hospitals, sponge devises in primary settings prior to transfer)

Additional characteristics emphasized by LMIC respondents include:

  - Reusability is valued
  - A broader range of settings, provider cadres, earlier usage were rated as essential or desirable
  - LMIC providers stress the need for in-person training

Detailed Results

Profession, all respondents

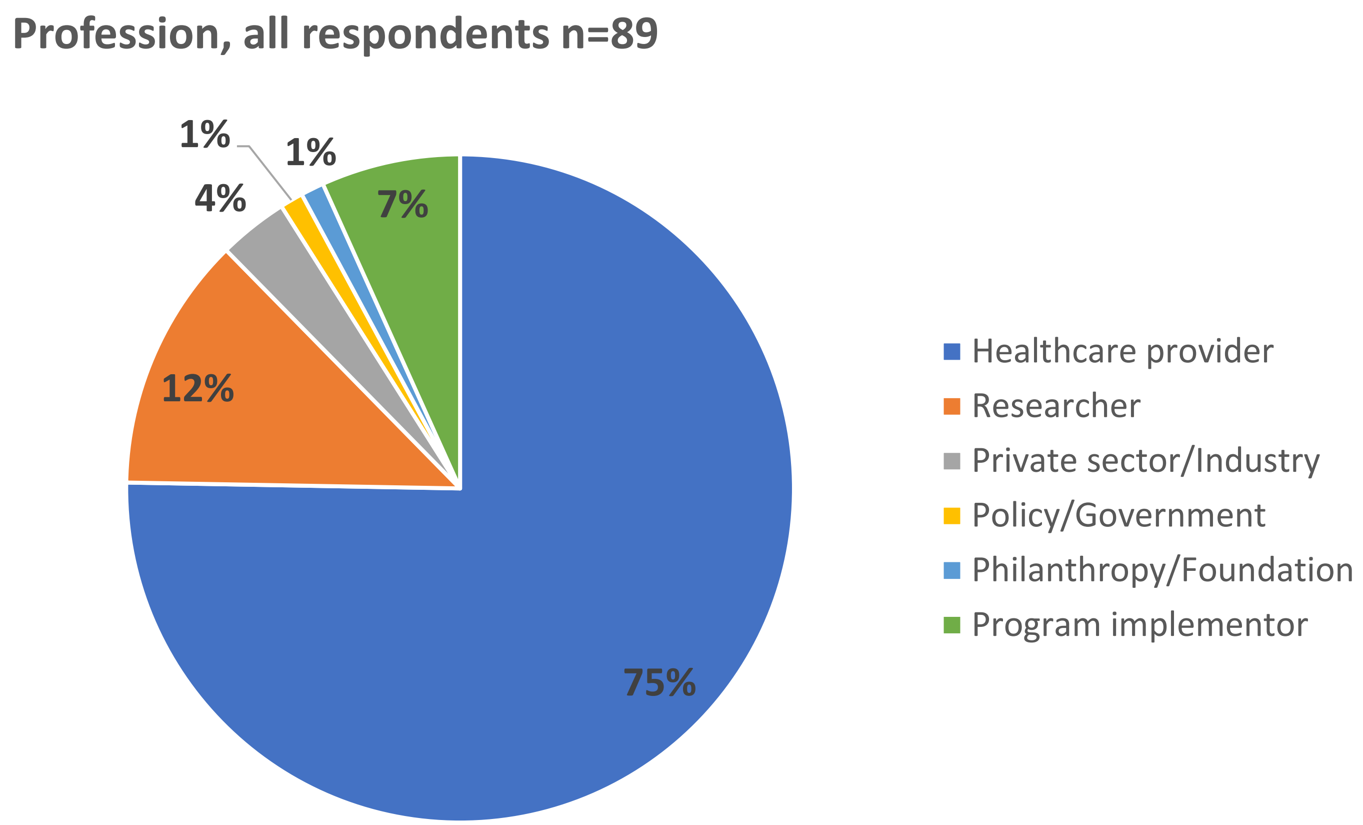

Healthcare providers, all respondents

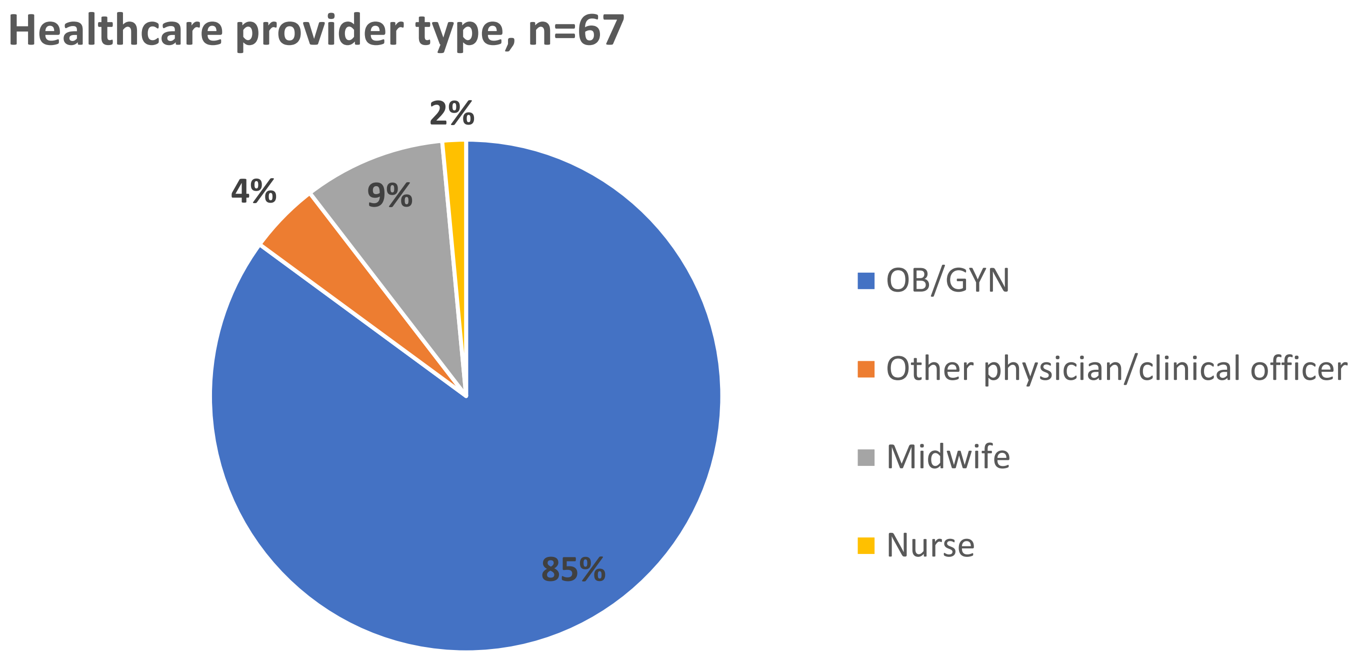

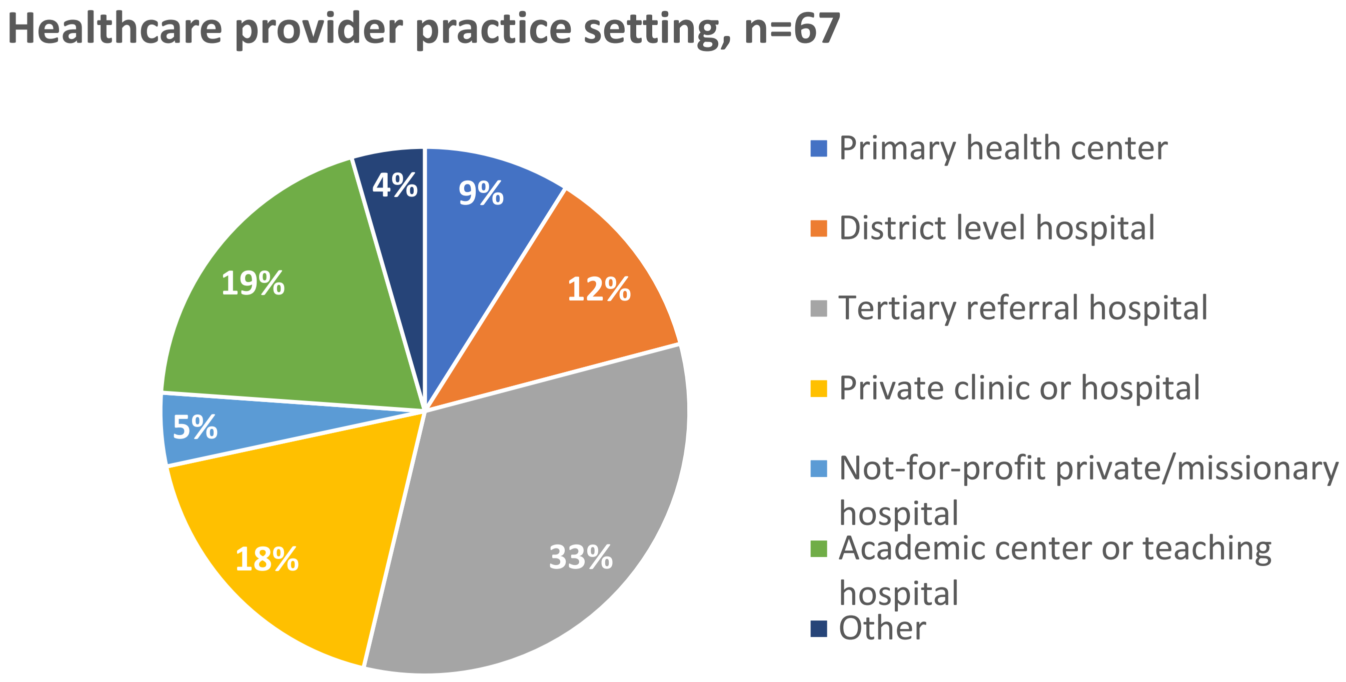

Profession by setting

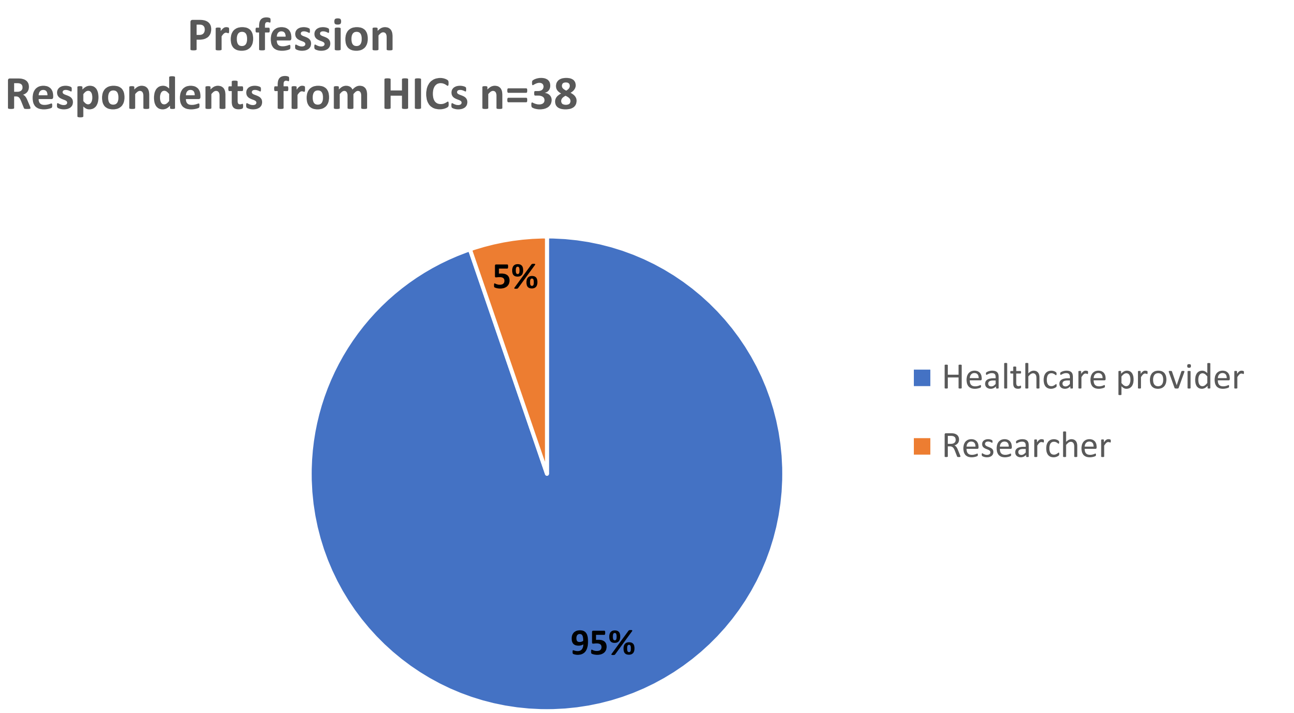

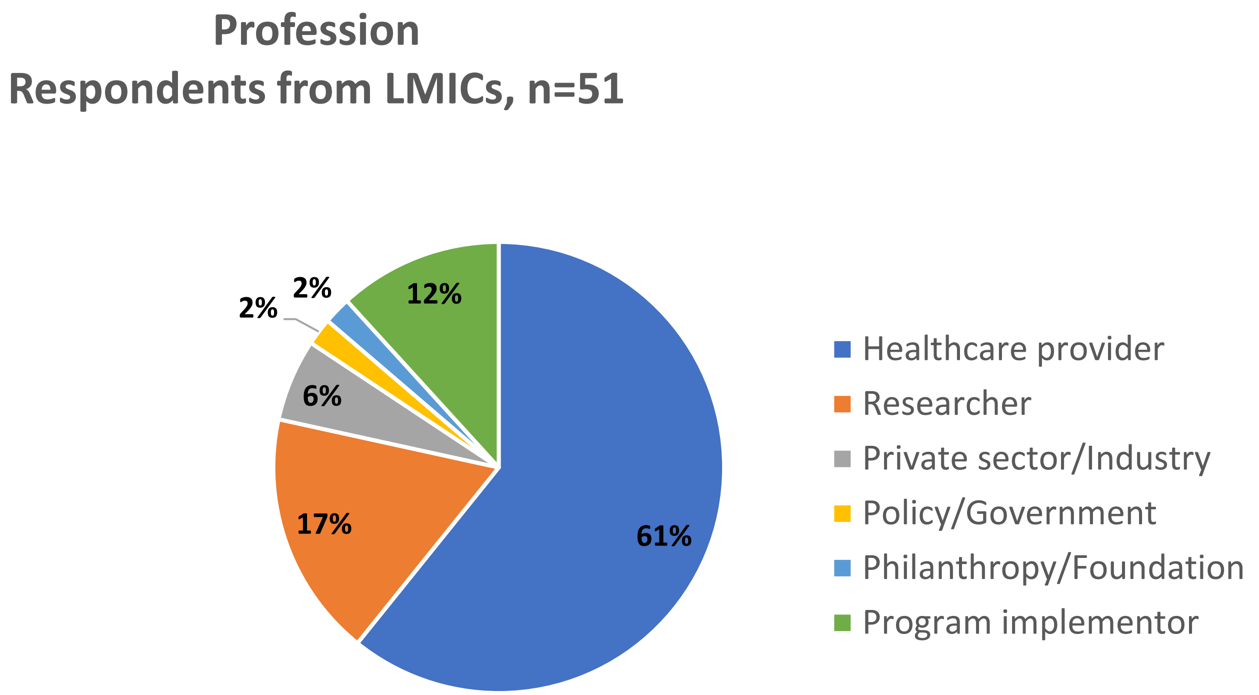

Healthcare providers by setting

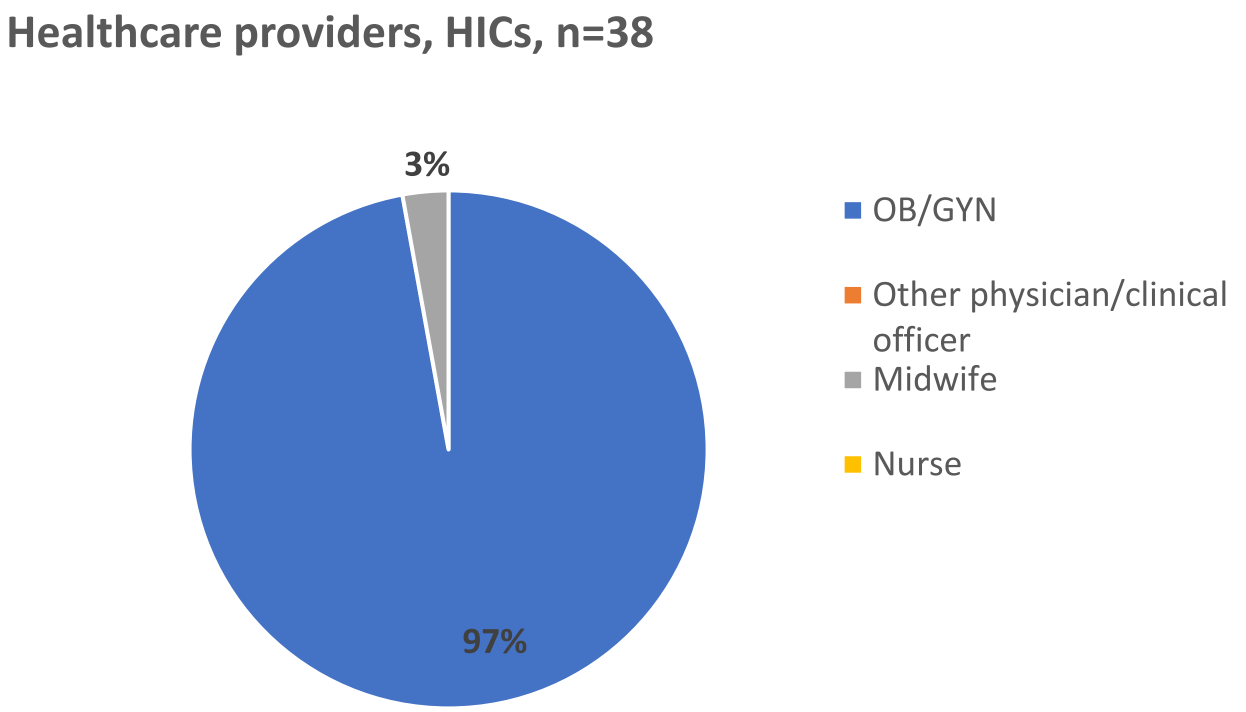

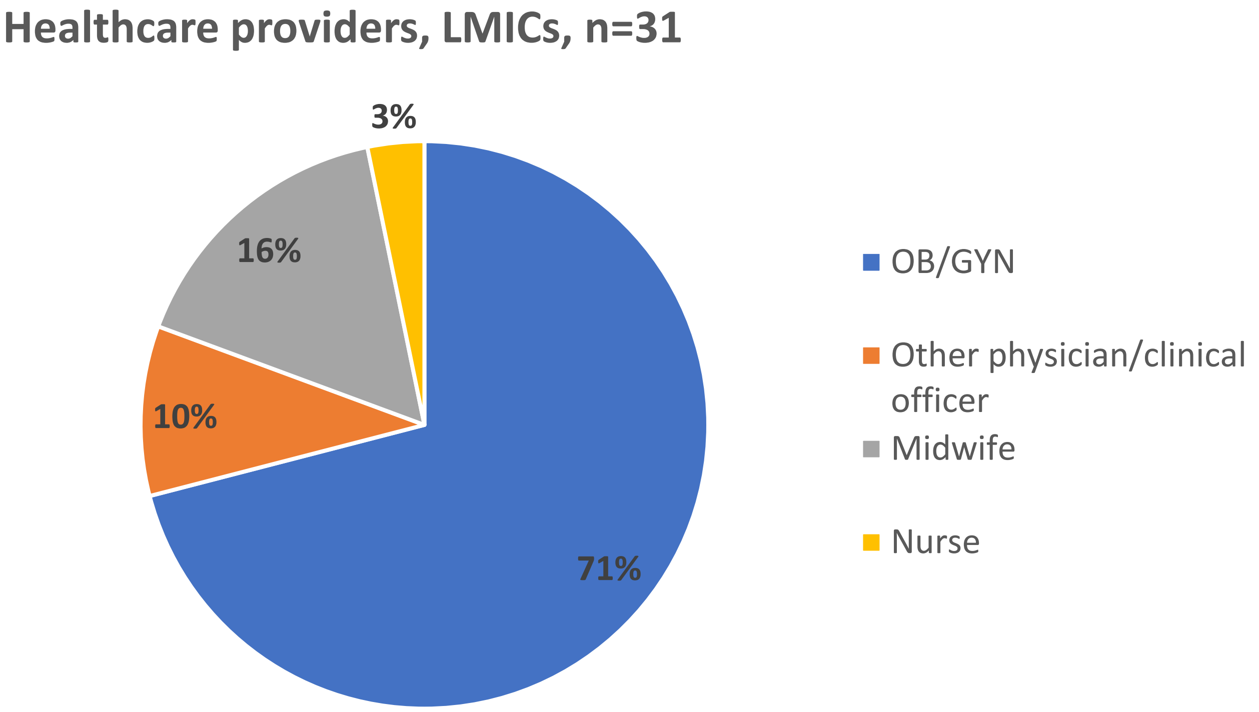

Clinical indication

We asked respondents which for clinical indications is it essential that a device can be used. Responses are displayed for all respondents and for the subset of respondents from LMICs.

- Most respondents considered it essential to be able to use tools for uterine atony after vaginal and cesarean delivery.
- Most respondents considered it desirable to be able to use tools for all other indications offered.

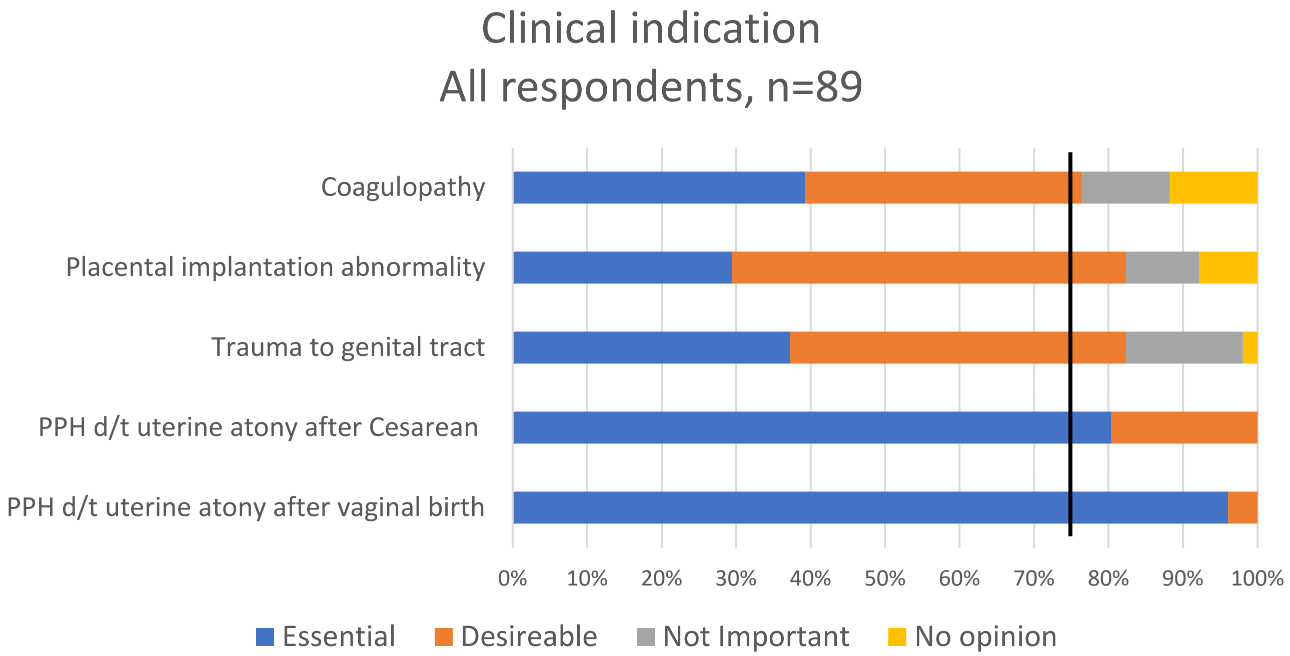

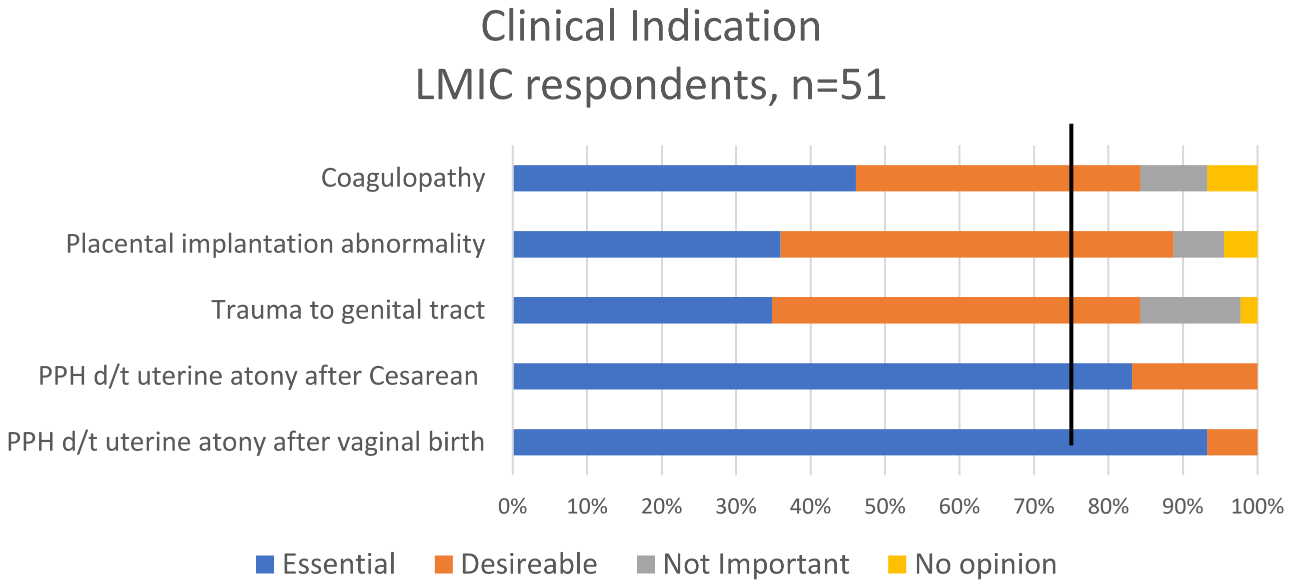

Timing

When asked what is the most important time in which to use such a device during PPH management, LMIC respondents were more interested in a device which could be used earlier.

- Most participants considered it desirable to be able to use these tools if bleeding continues after the initial response or all other measures have been exhausted.
- Respondents in low-resource settings considered the ability to use these tools after the initial response to be essential.

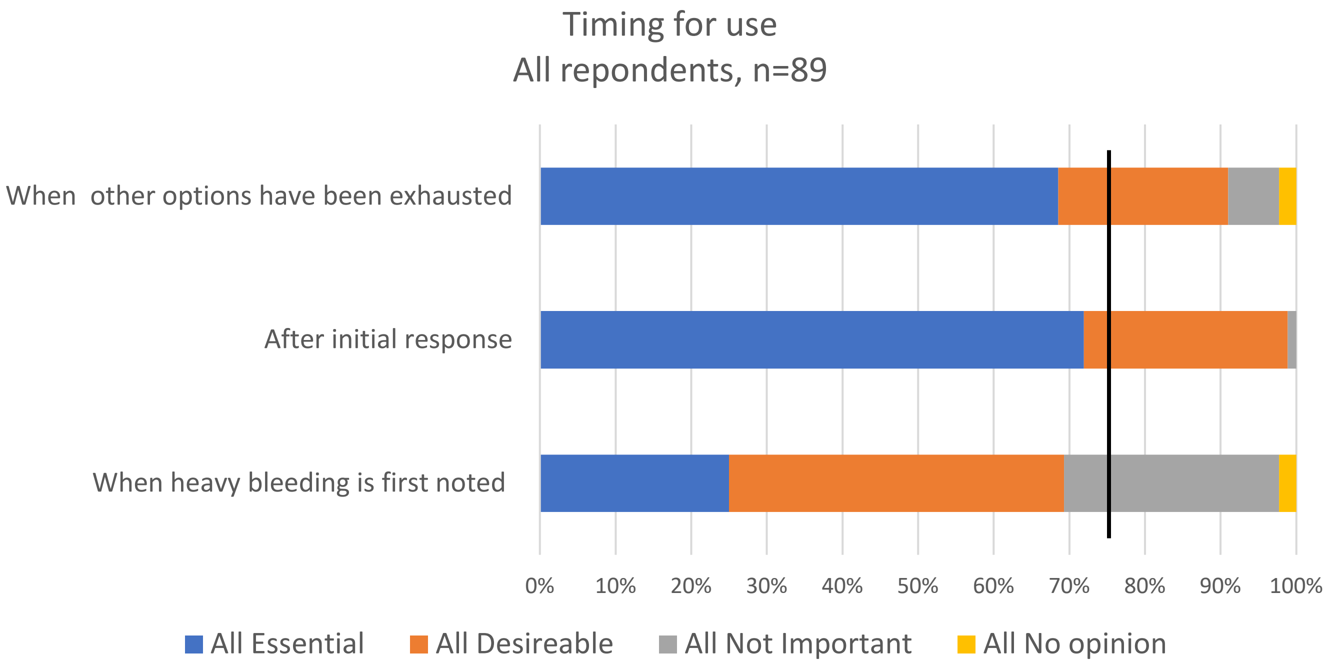

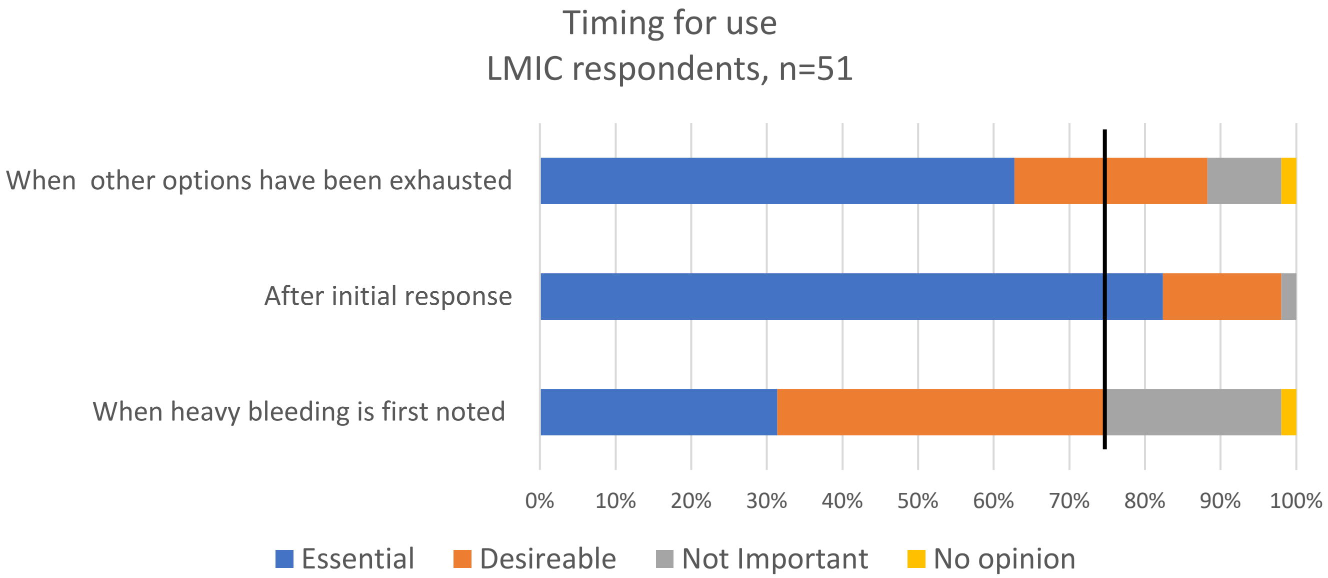

Efficacy for prevention of surgery

None of the efficacy thresholds met the threshold for essential. However, most respondents agree it would be desirable for these tools to prevent surgical intervention provided it was as effective or better than standard of care and there was no more than a minimal increase in risk.

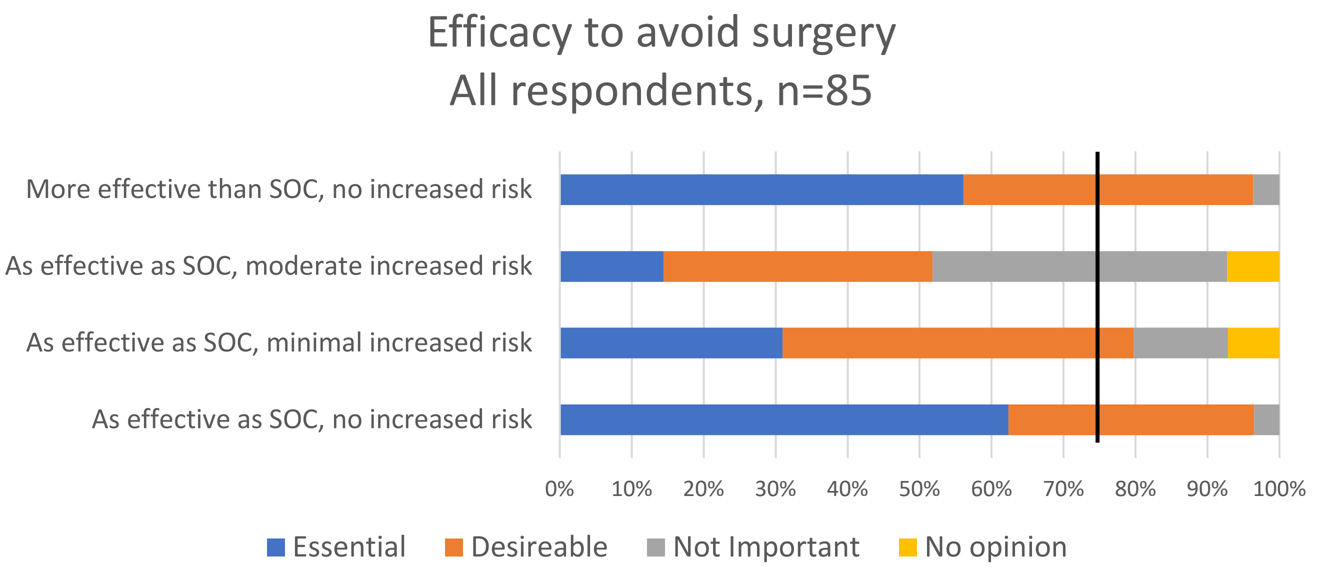

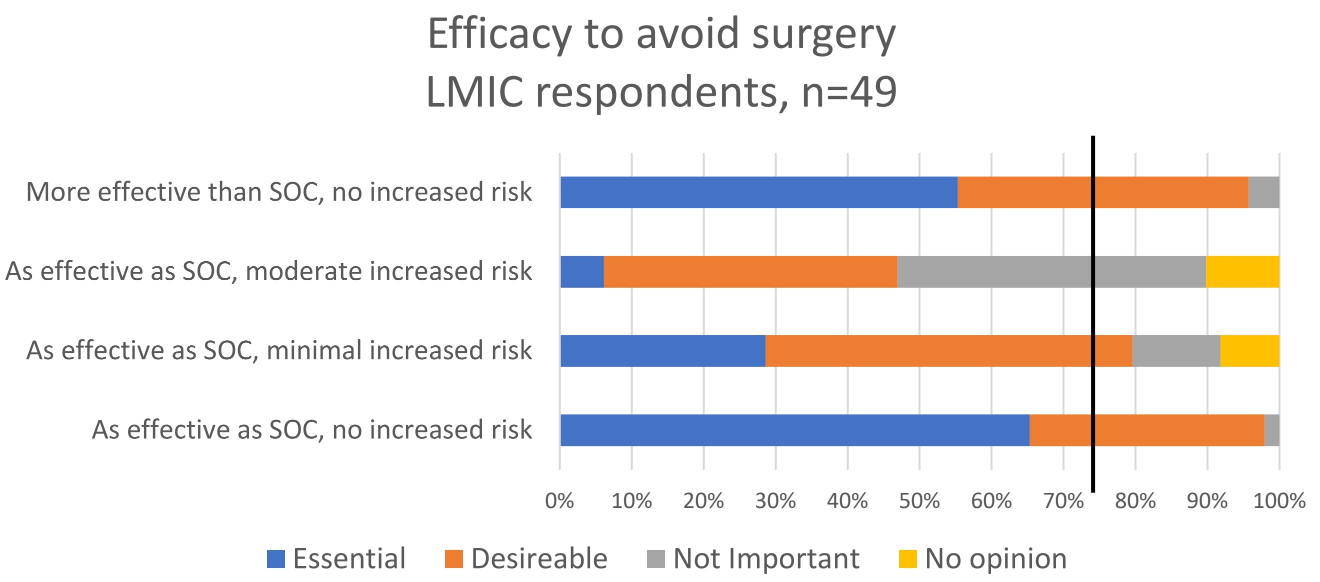

Efficacy for prevention of blood loss greater than 1000ml

While most respondents agree it would be desirable for these tools to prevent blood loss (BL) greater than 1000ml at least as well or better than standard of care provided there was no more than a minimal increase in risk, it was not considered essential.

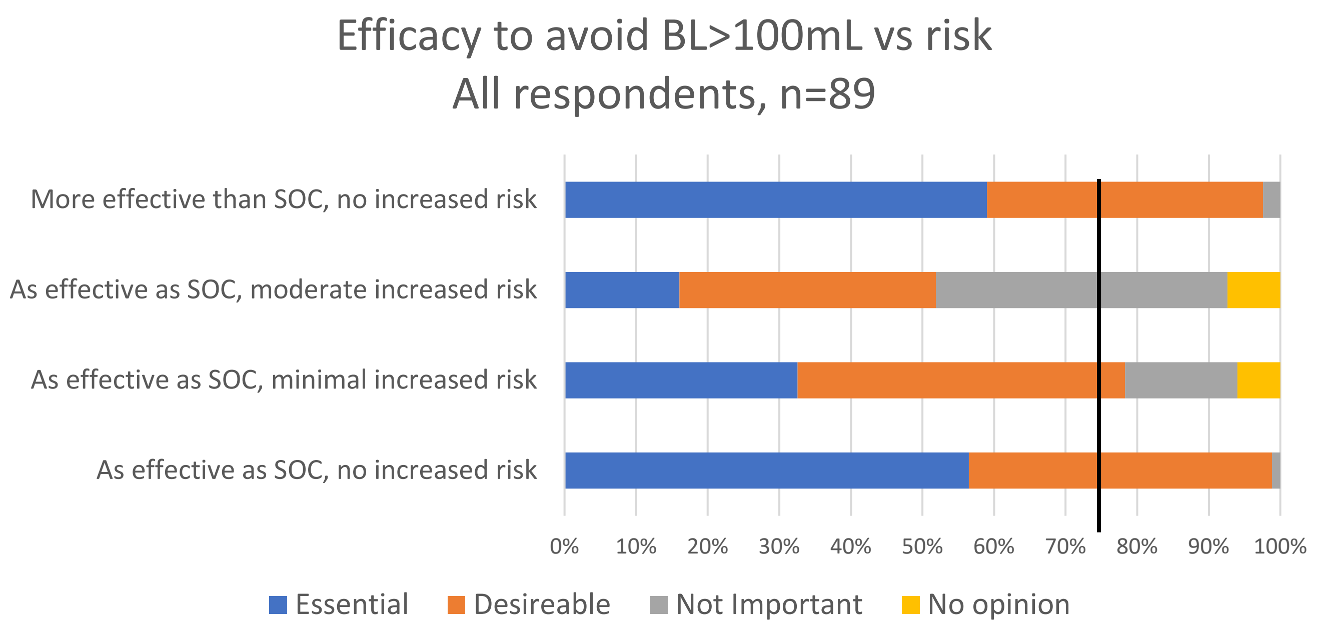

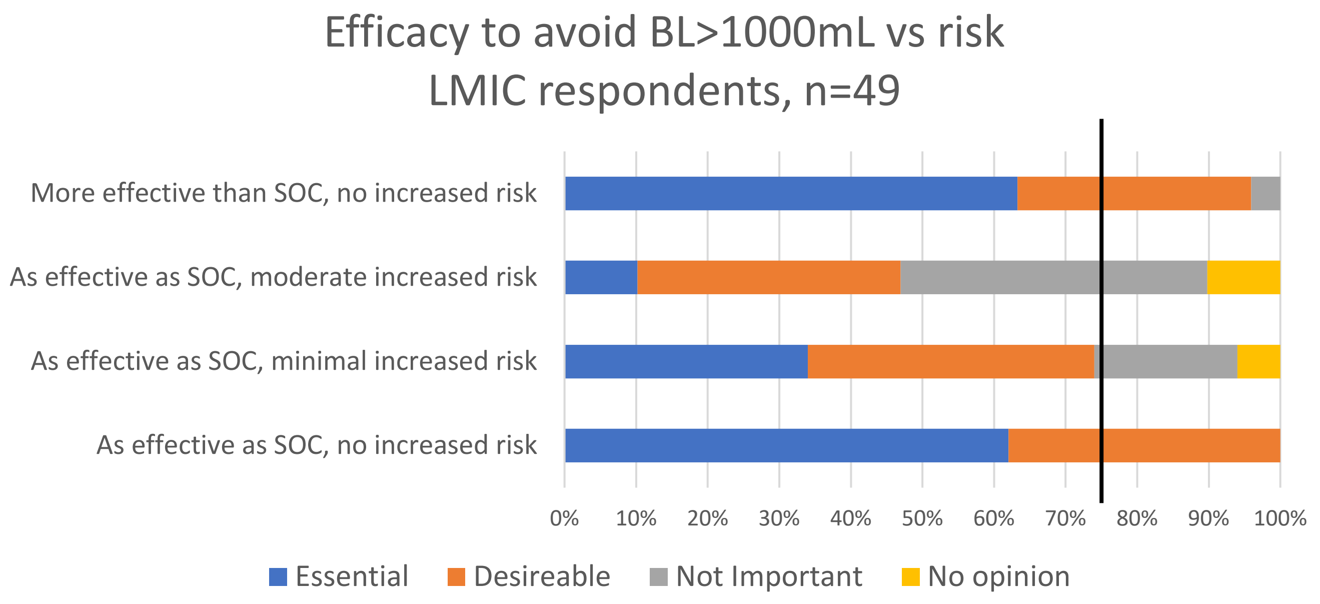

Setting

When asked which setting these devices should be used in:

- Tertiary and district hospitals are considered an essential place to find these tools by most respondents.
- Low-resource setting respondents also consider private clinics or hospitals to be essential.
- Most respondents agree it would be at desirable to have these tools available at primary health centers or during transport.

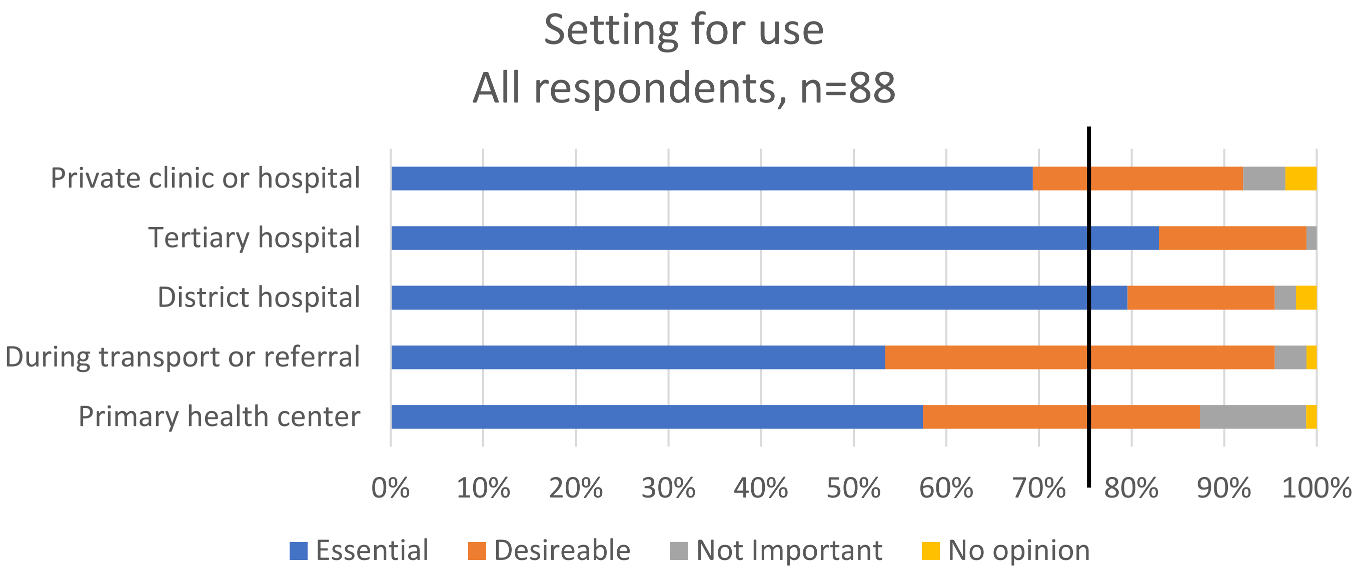

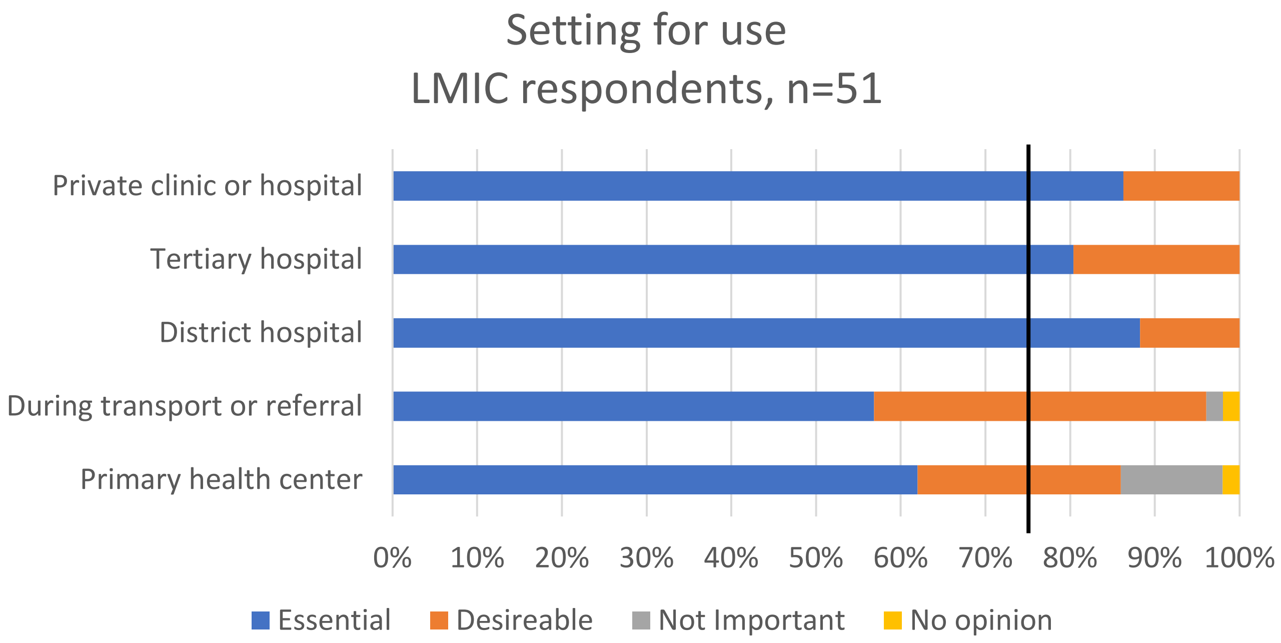

Provider cadre

- Usability by OBGYNs is considered essential.
- Low-resource setting respondents additionally consider use by other medical officers, clinical officers, and midwives to be essential. Usability by nurses and emergency care technicians during referral is also desirable.
- Use by Community Health Workers or Traditional Birth Attendants is not valued by most respondents.

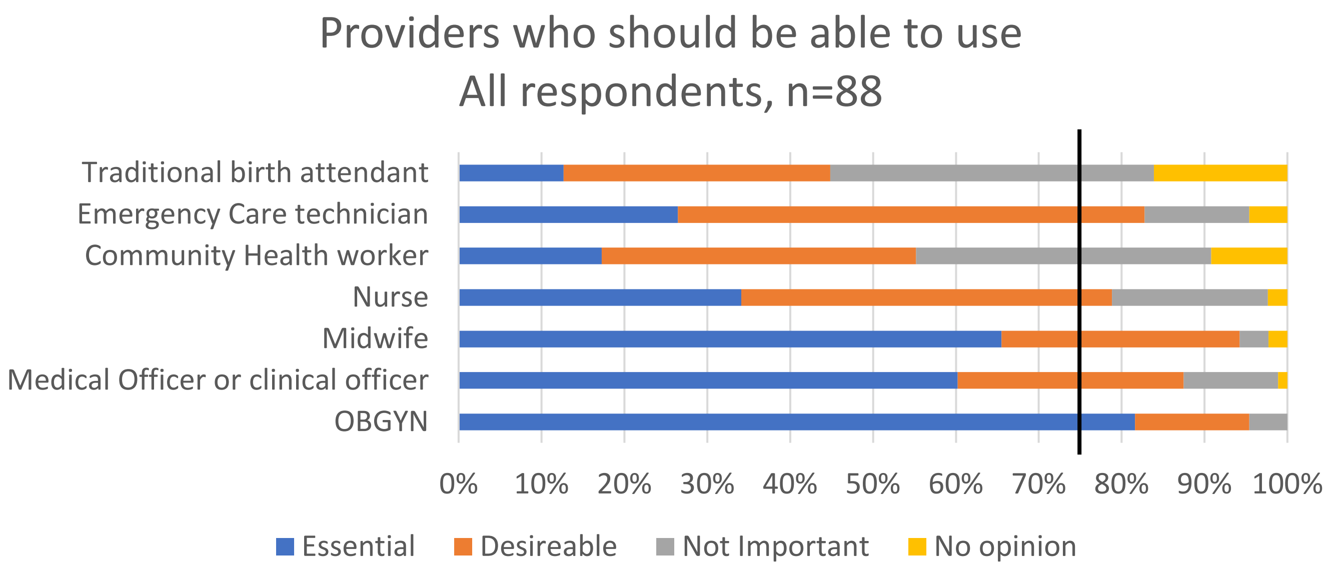

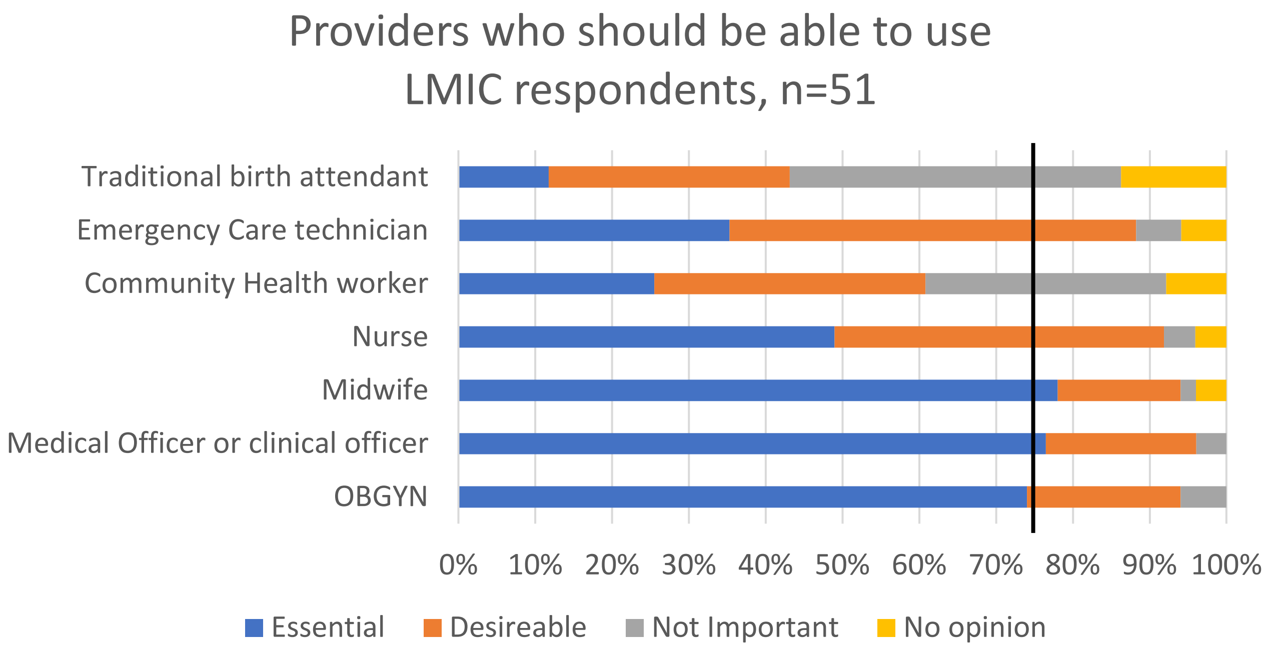

Training

Interestingly, LMIC respondents placed greater importance on training strategies for these tools.
Low-resource setting participants feel strongly that in-person training with simulation is needed, and on-going mentorship is highly desirable.

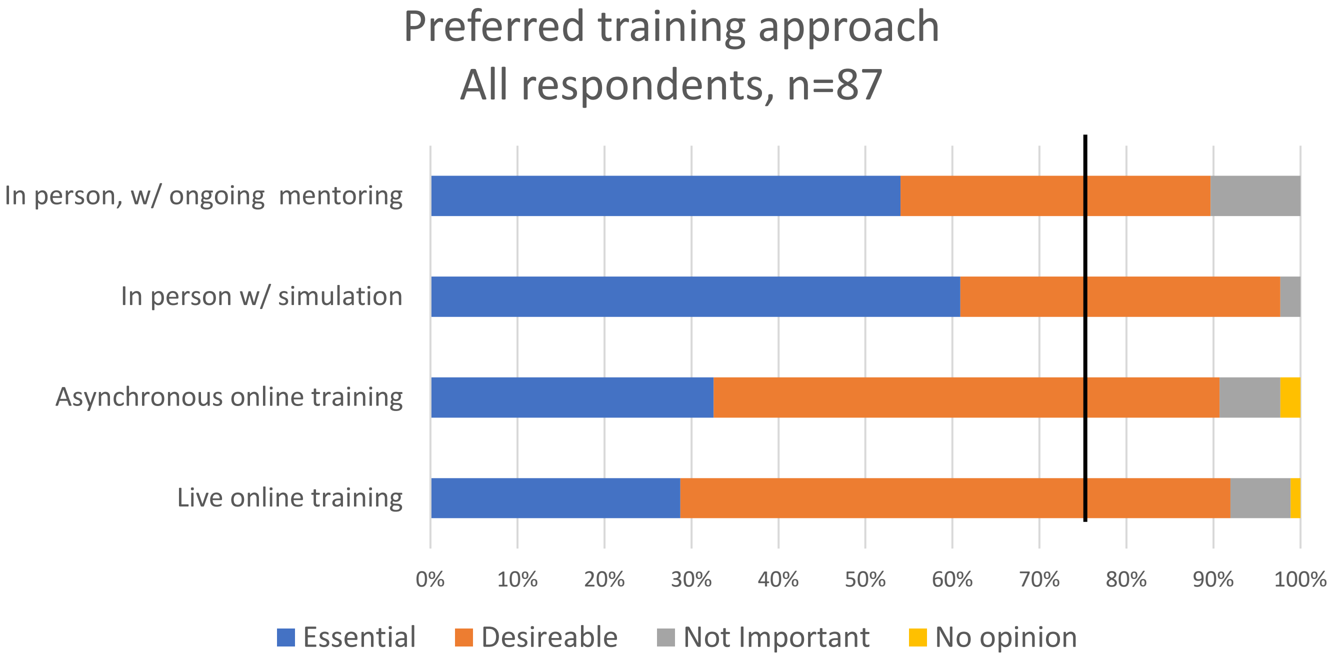

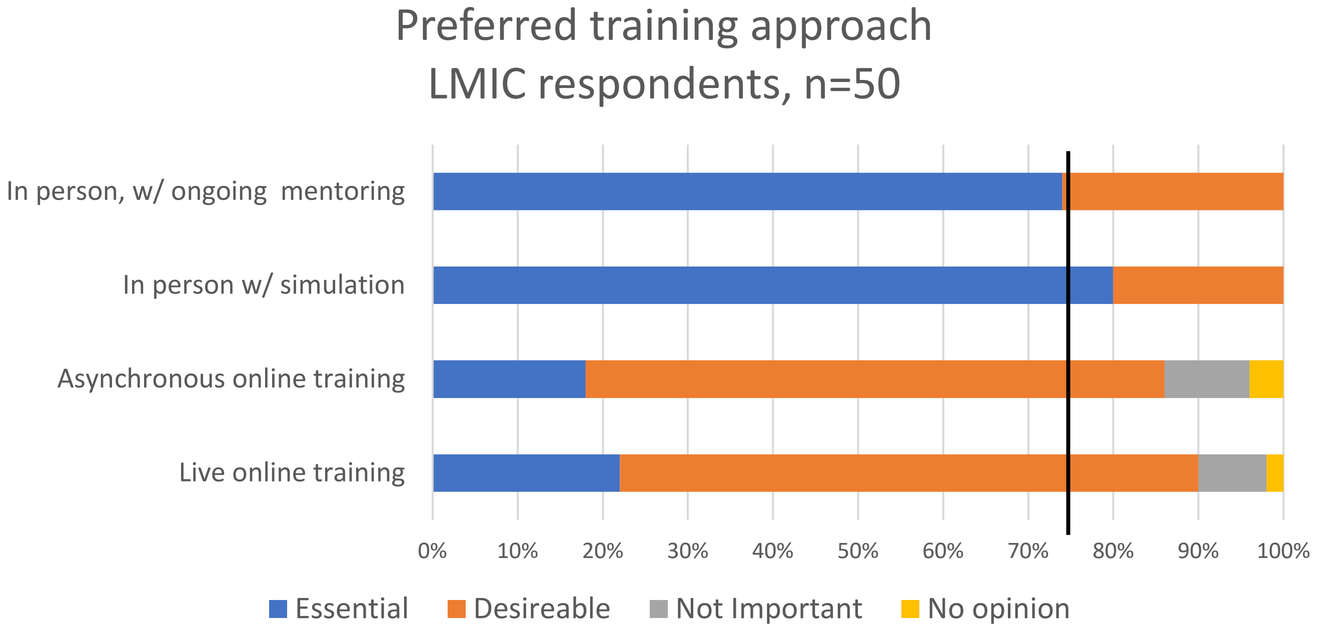

Competency

We asked respondents to indicate what level of experience should be needed for a provider to reach competency. The results below show that 2-5 insertions to gain competency is most desirable.

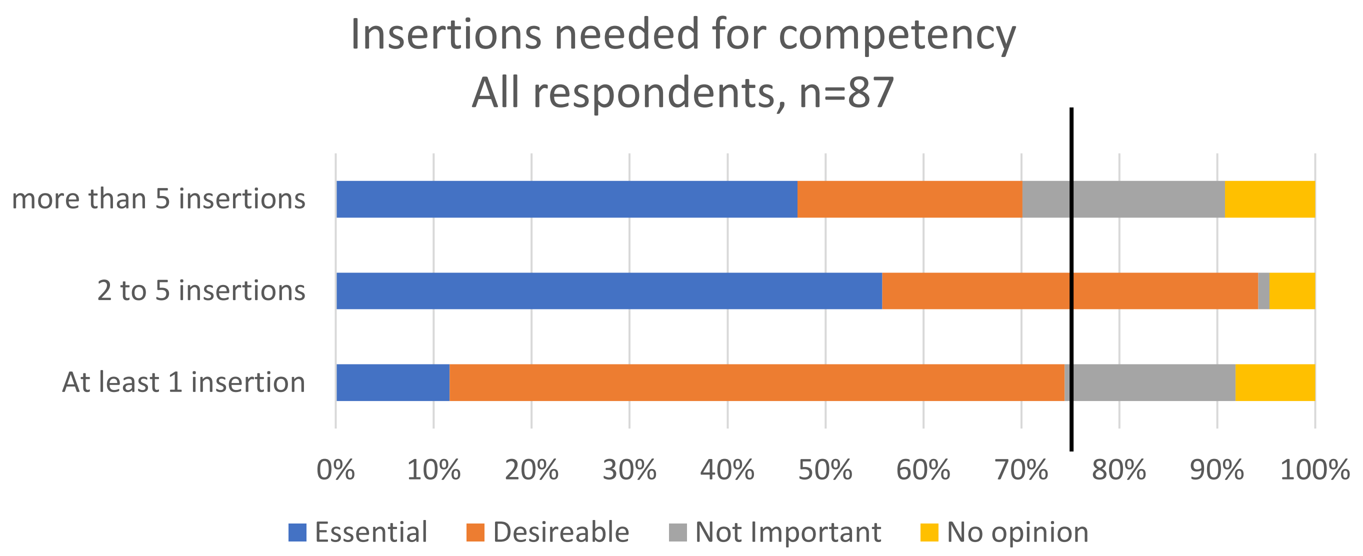

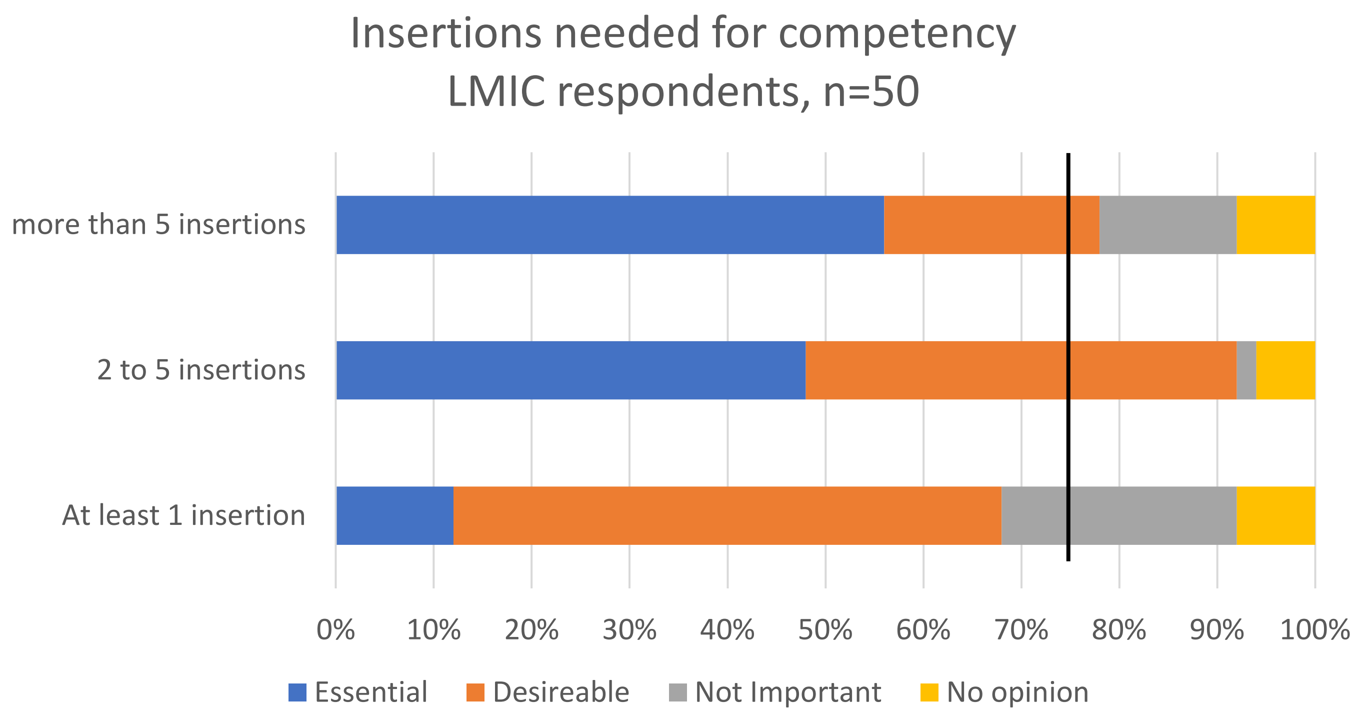

Ease of insertion

When asked about how important ease of insertion is, most characteristics were rated as desirable:

- Little to no assembly, quick insertion, and the ability to be inserted by one provider are all desirable.
- Low-resource setting providers also desire a tool that can be used with one hand.

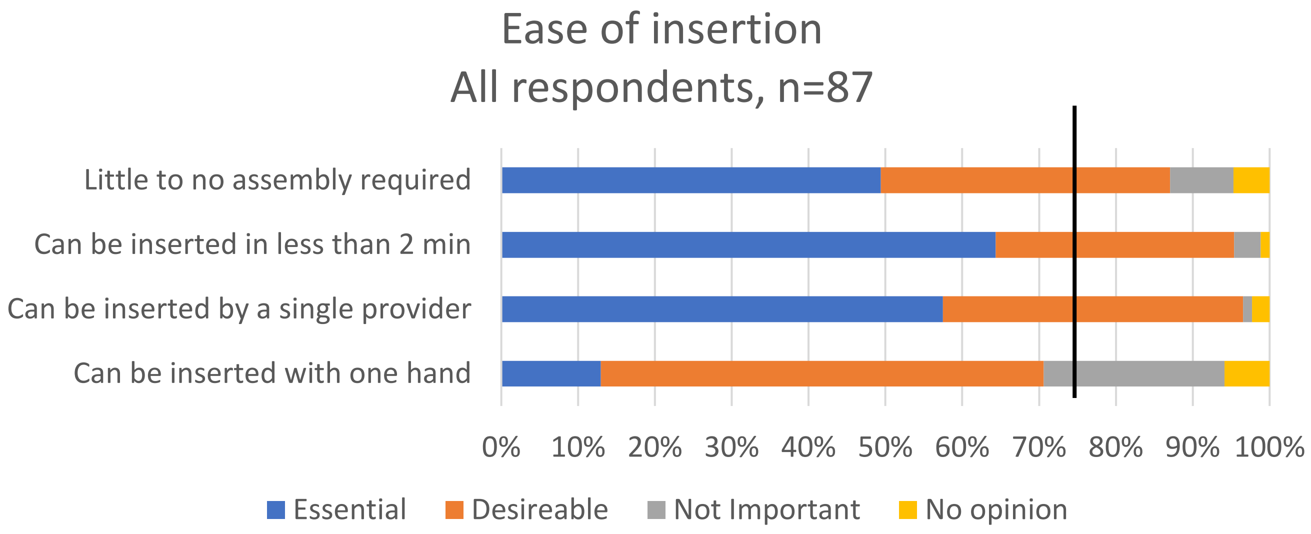

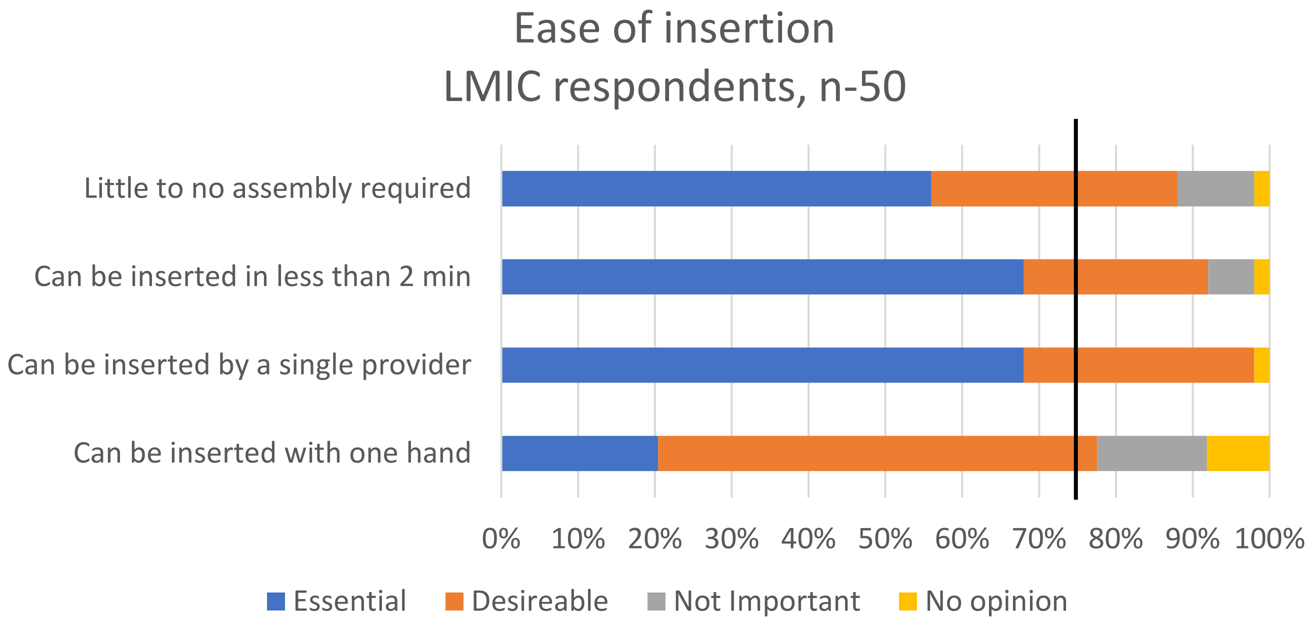

Treatment duration

Duration of treatment is an important characteristic from the perspective of patient experience and system impact. Respondents reported:

- Treatment should be comparable to or shorter than standard care, and </= 24 hours.
- Low-resource setting providers find <6 hours desirable.

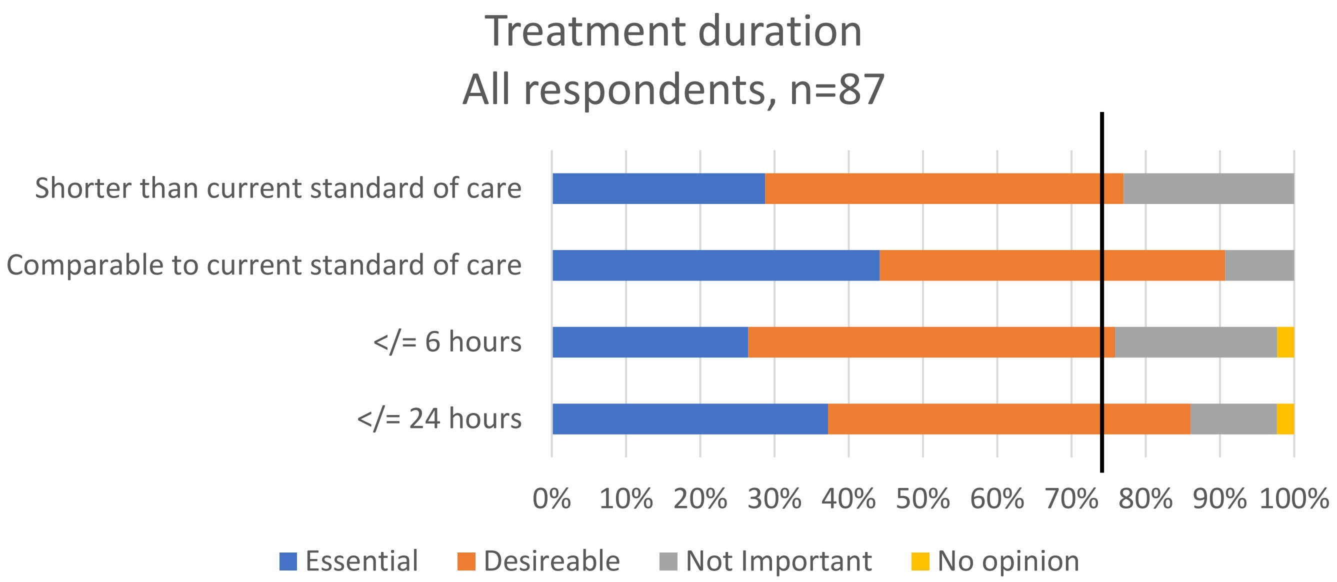

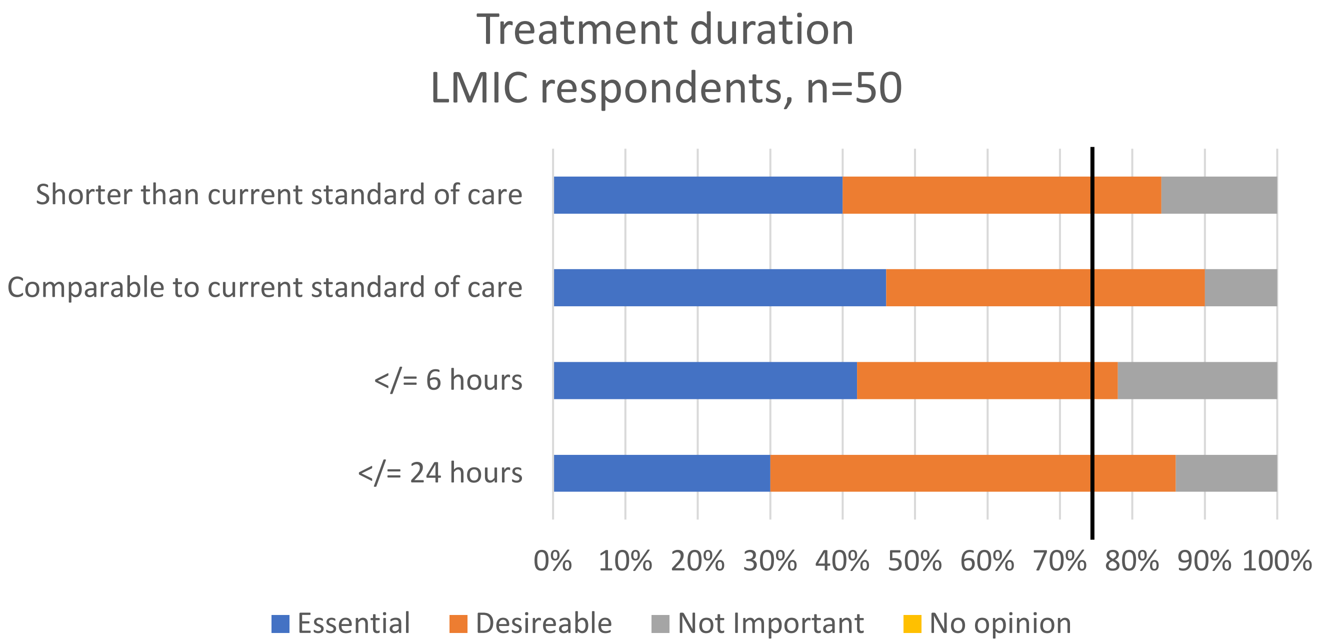

Patient experience

We asked respondents for their opinion on patient experience compared to standard of care. Most respondents agreed patient experience should be better or similar to standard care.

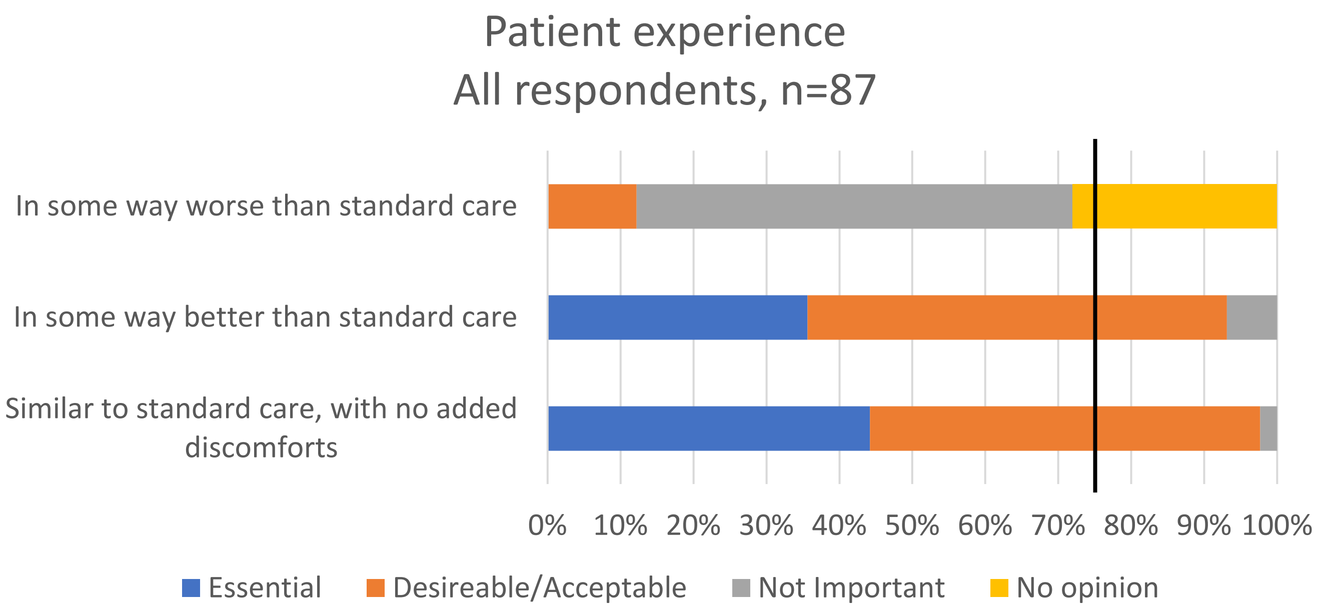

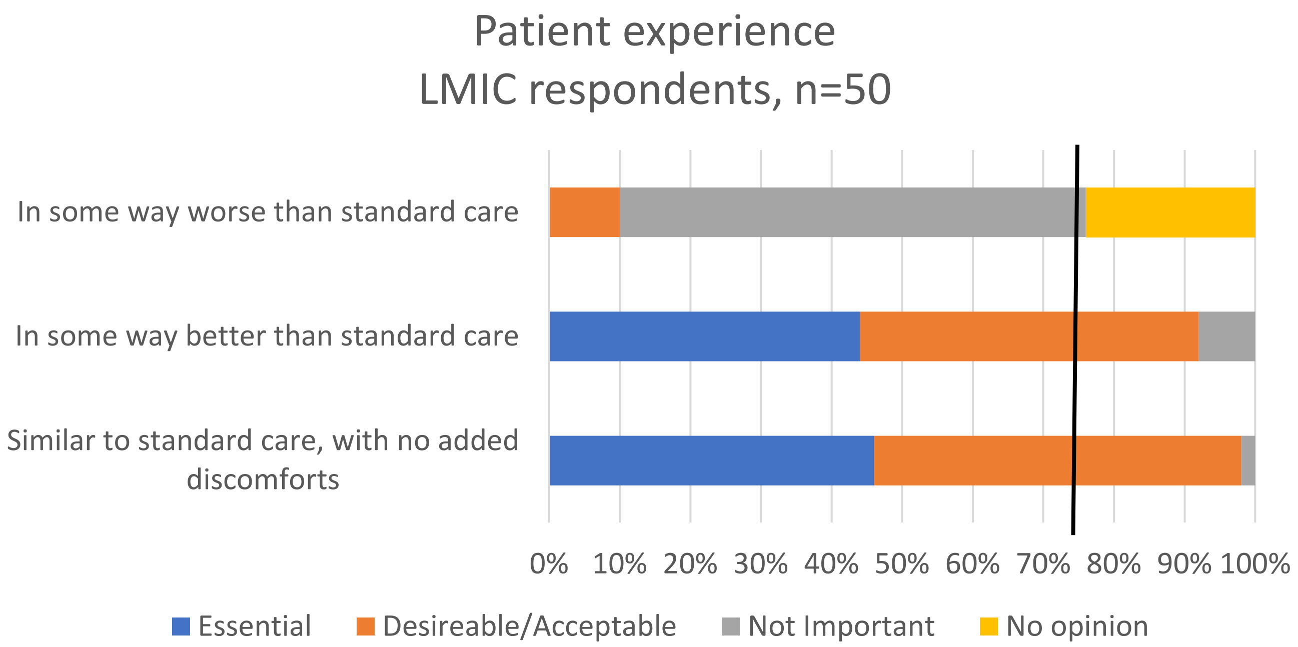

Reusability

- Reusability is seen as desirable but not essential.

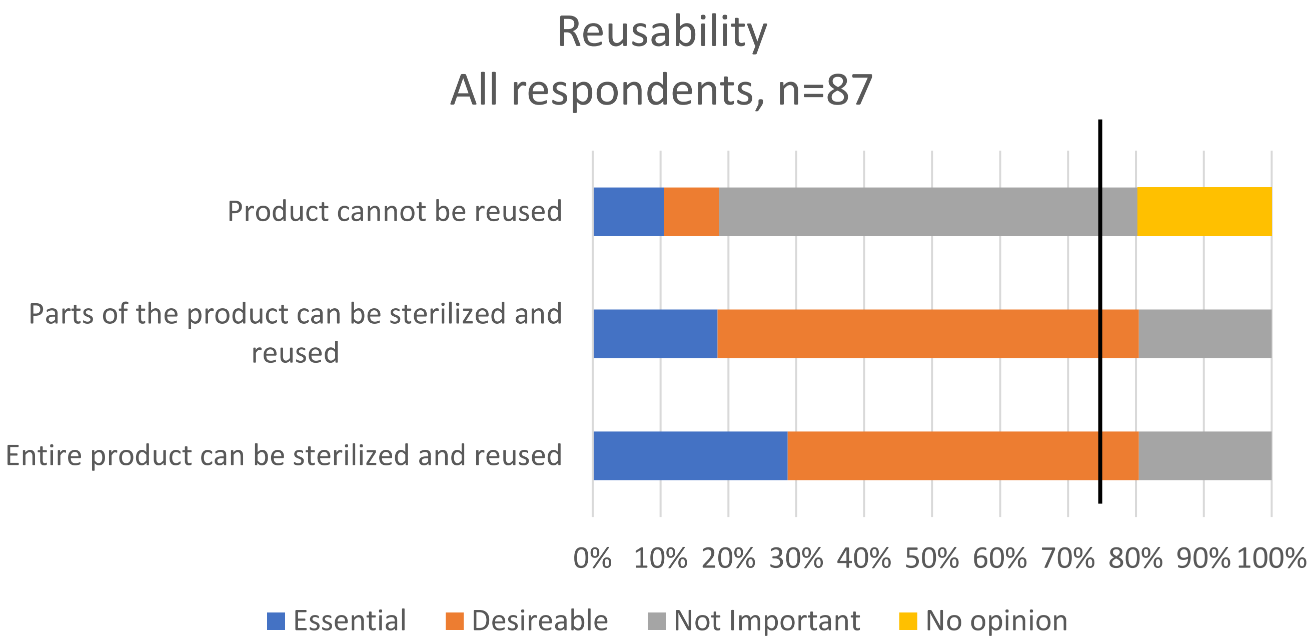

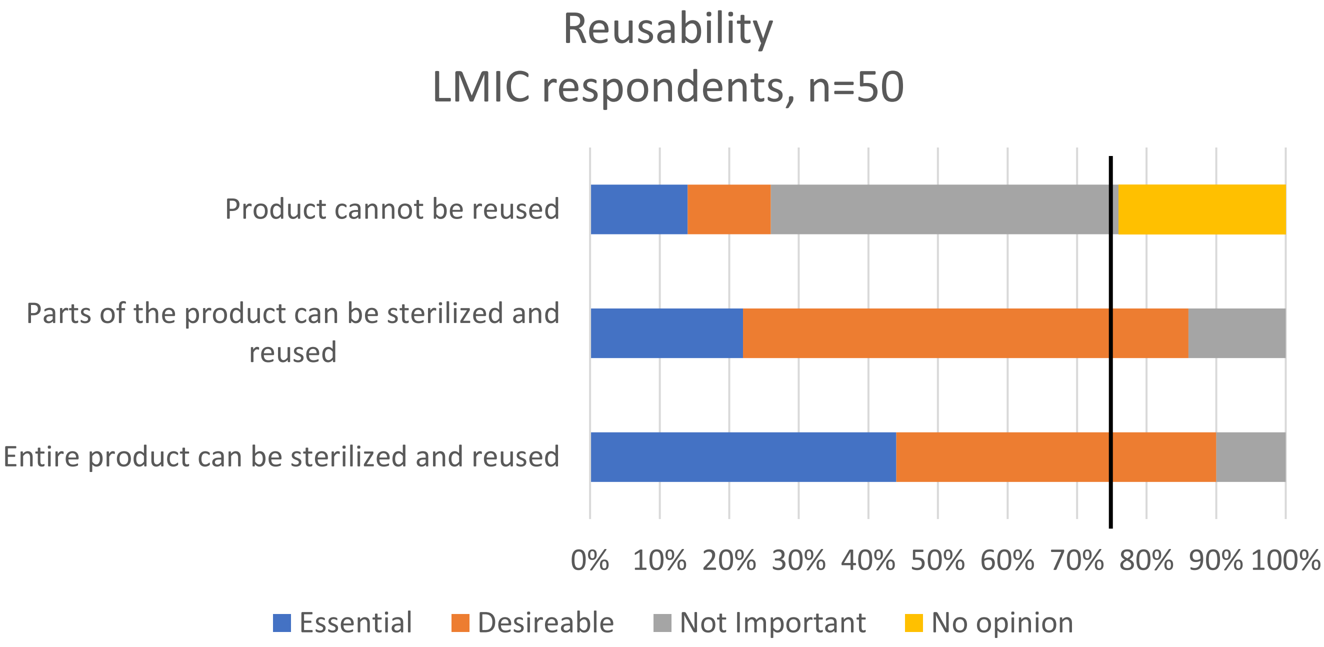

Storage

- For low-resource settings, storage should be at ambient temperature.
- Ability to withstand high ambient temperatures is desired in low-resource settings.
- Cold chain storage is not desirable.

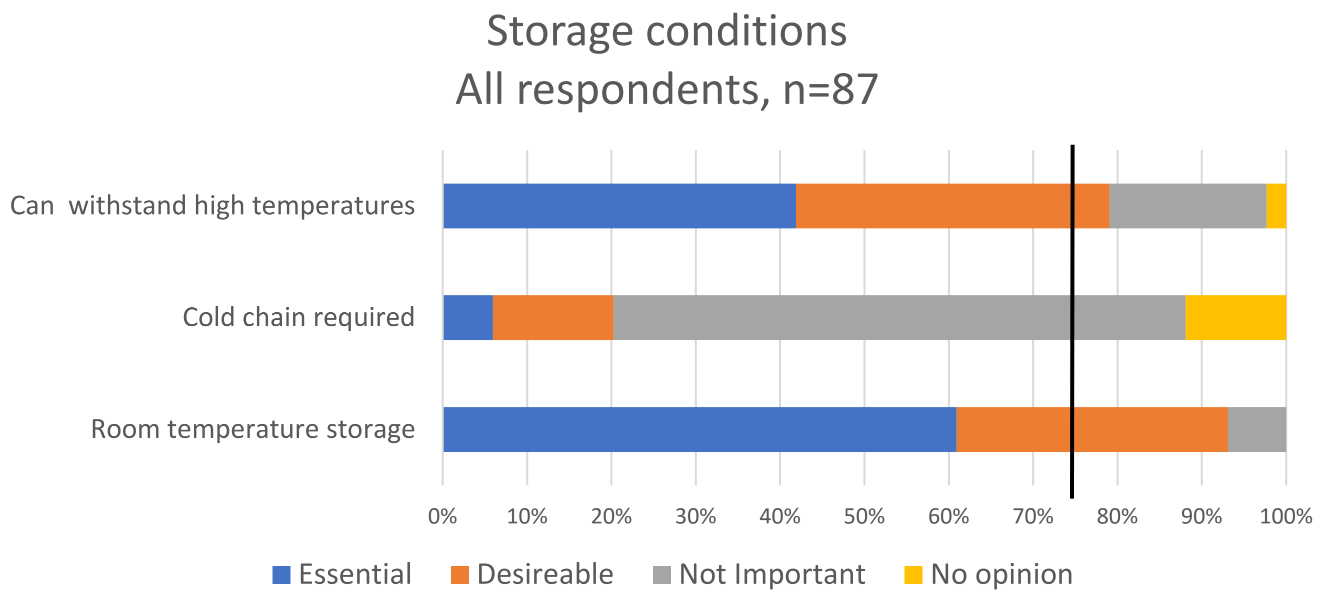

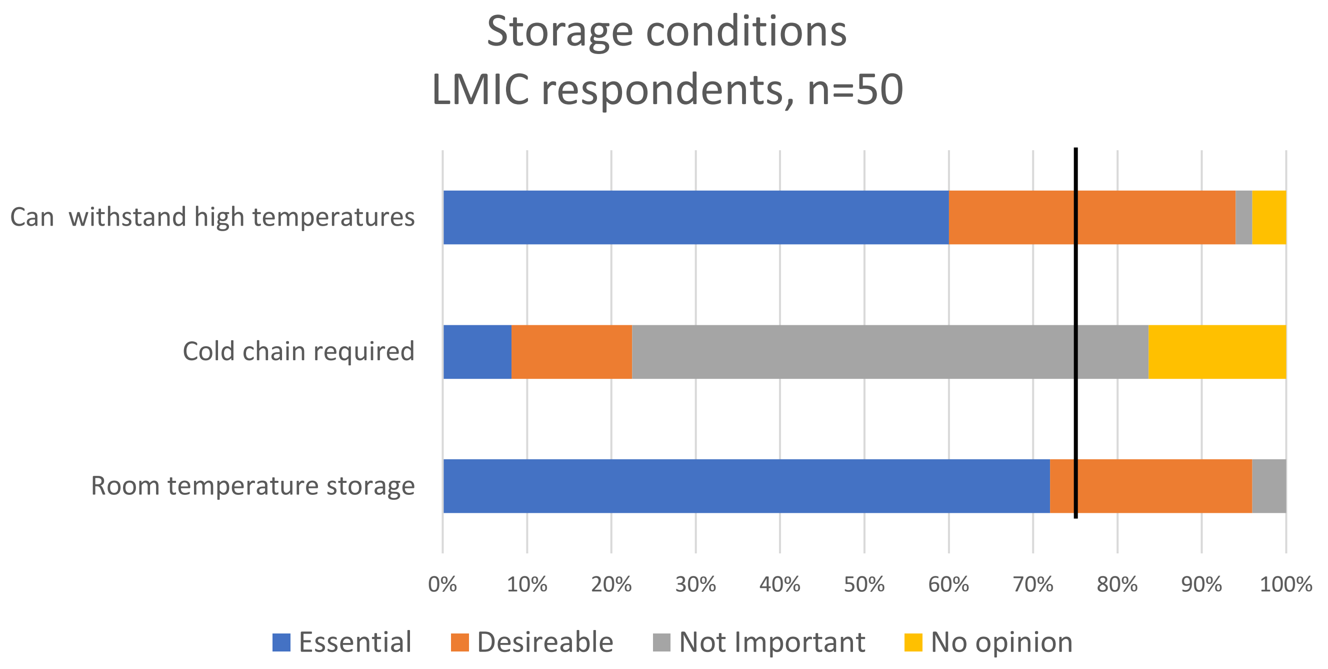

Cost

We asked respondents about whether cost was an important factor in deciding to adopt a tool. Not surprisingly, while low-cost products are preferred generally, this was even stronger among LMIC respondents.

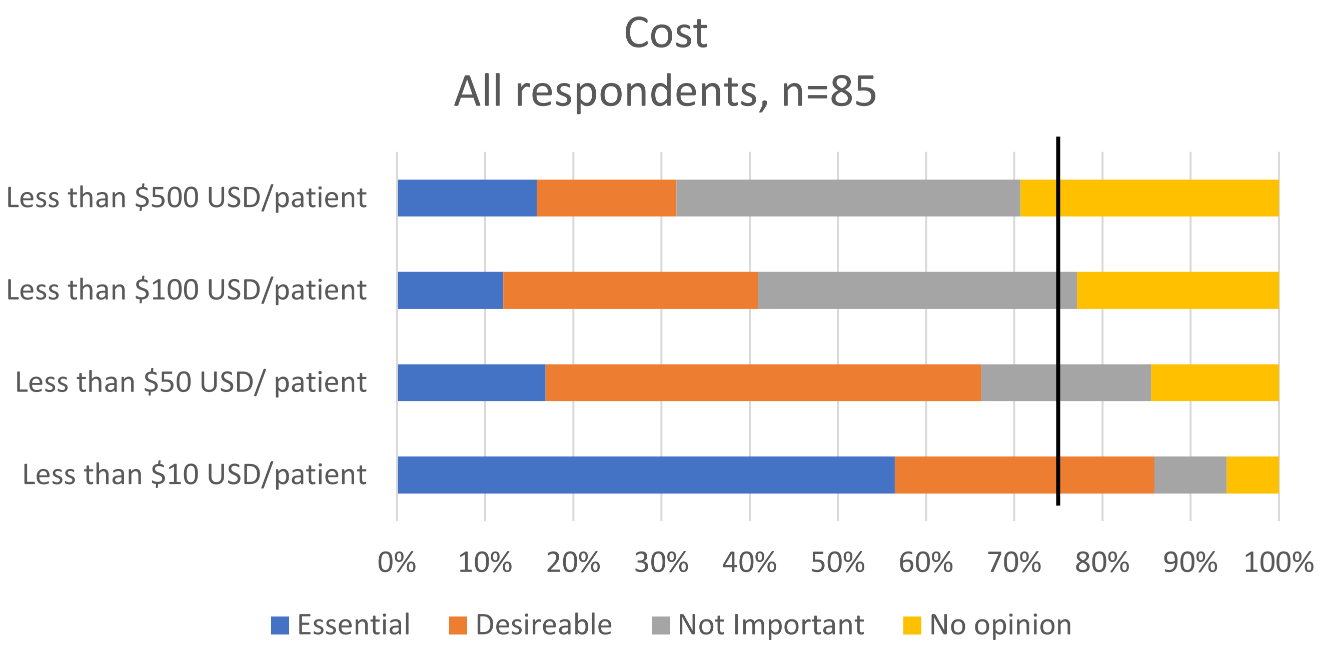

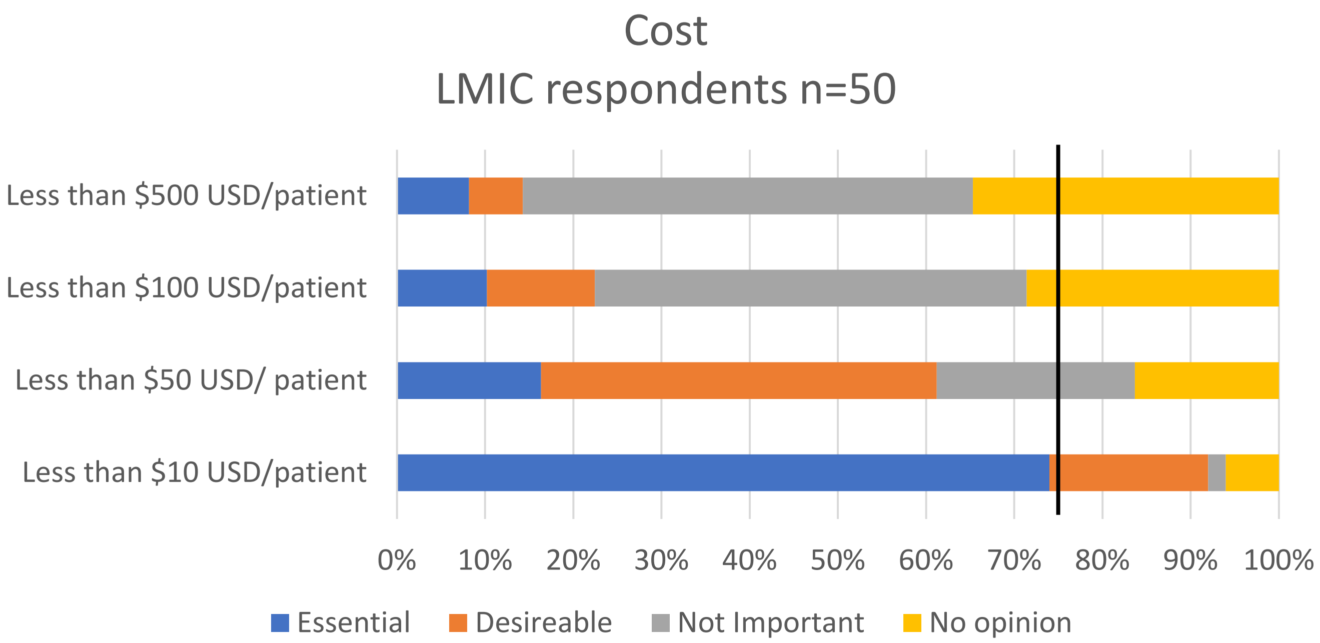

Regulatory approvals

- Tools with regulatory approval for PPH are preferred.
- Tools with regulatory approval for other conditions may also be considered.

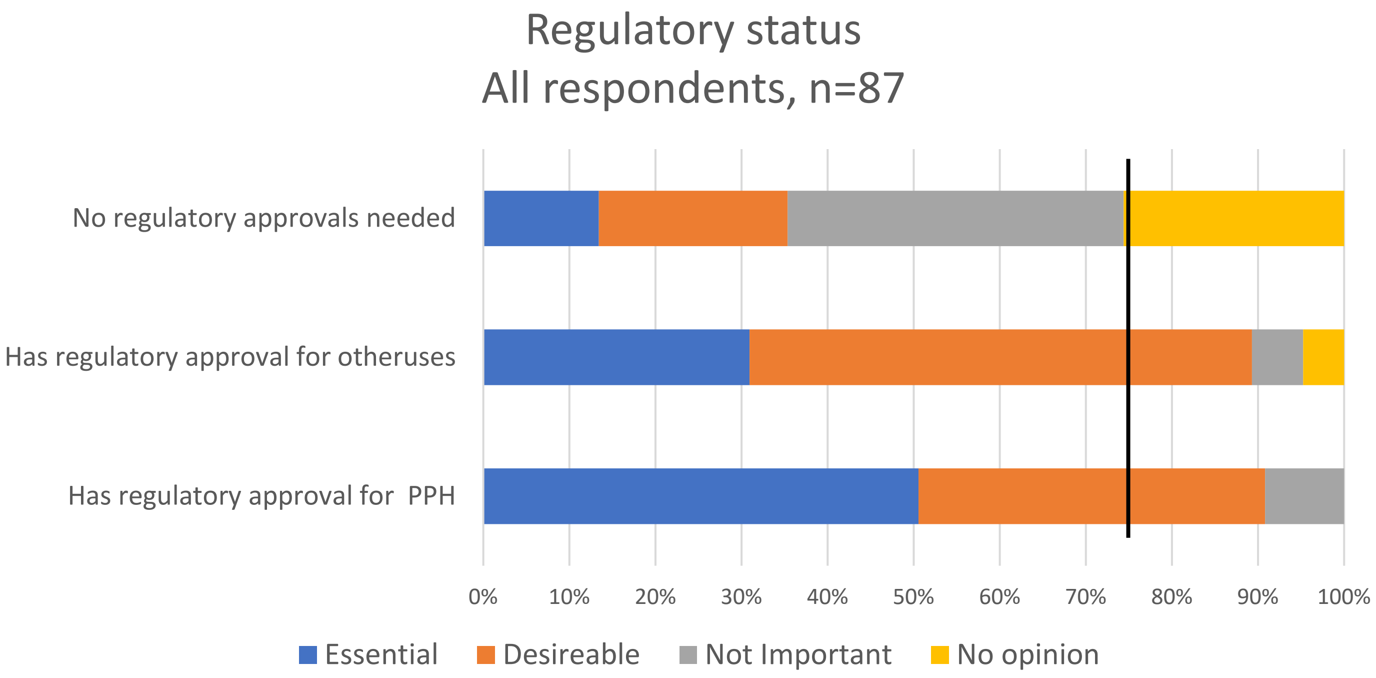

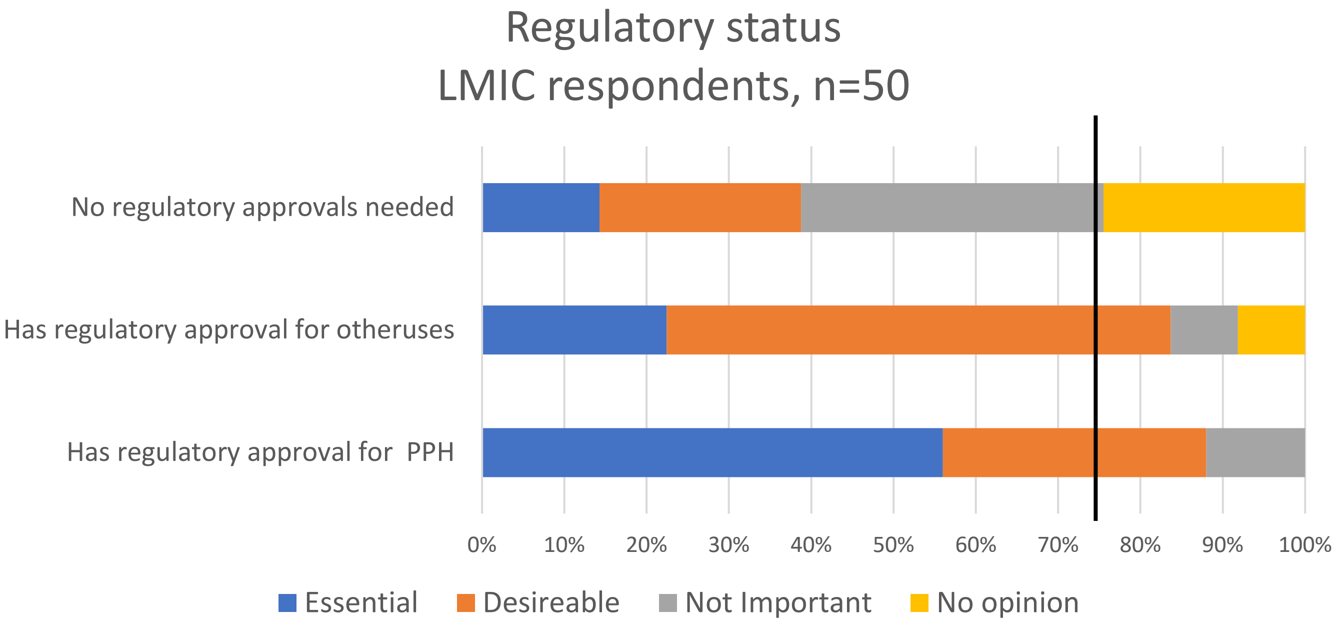

Limitations

- In an attempt to make the questions consistent, the terms Essential and Desired were applied through the rating of characteristics. However, in some instances, this terminology may have inhibited understanding for some respondents. This is demonstrated in slightly higher than average rates of non-response or “no opinion” to some questions. We addressed this by presenting responses as a proportion of all those responding, given that no question had a non-response rate greater than 10%.
- The survey presented a suite of emerging tools and then asked general questions about characteristics a tool should have. The heterogeneity of the tools in existence may have made rating characteristics difficult for some respondents if they were attempting to link characteristics to specific tools under development.
- The survey was conducted online using a forced choice format. The online nature may have limited who could access it. The forced choice format may have affected whether some respondents felt comfortable responding to all questions and completing the survey.
- We used 75% as the threshold for consensus based on other examples in the literature. This may have been too limiting as it produced few characteristics with consensus.
- This survey included a small sample size with limited demographic information and thus may not be generalizable. LMIC respondents and clinicians were over-represented compared to researchers and policy makers. Fewer than 20% of those approached by email completed the survey.
